# Supplementary material for: Synchronized temporal-spatial analysis via microscopy and phosphoproteomics (STAMP) of quiescence
Source: Sci Adv. 2025 Apr 25;11(17):eadt9712. doi: 10.1126/sciadv.adt9712 (PMC12024681; doi:10.1126/sciadv.adt9712)
Supplement: Supplementary file 1 — Figs. S1 to S8 Data S1 [file sciadv.adt9712_sm.pdf]

Science Advances
AAAS

Supplementary Materials for

Synchronized temporal-spatial analysis via microscopy and phosphoproteomics (STAMP) of quiescence

Mohammad Ovais Azizzanjani *et al.*

Corresponding author: Peter K. Jackson, pjackson@stanford.edu

Sci. Adv. **11**, eadt9712 (2025)
DOI: 10.1126/sciadv.adt9712

This PDF file includes:

Figs. S1 to S8
Data S1

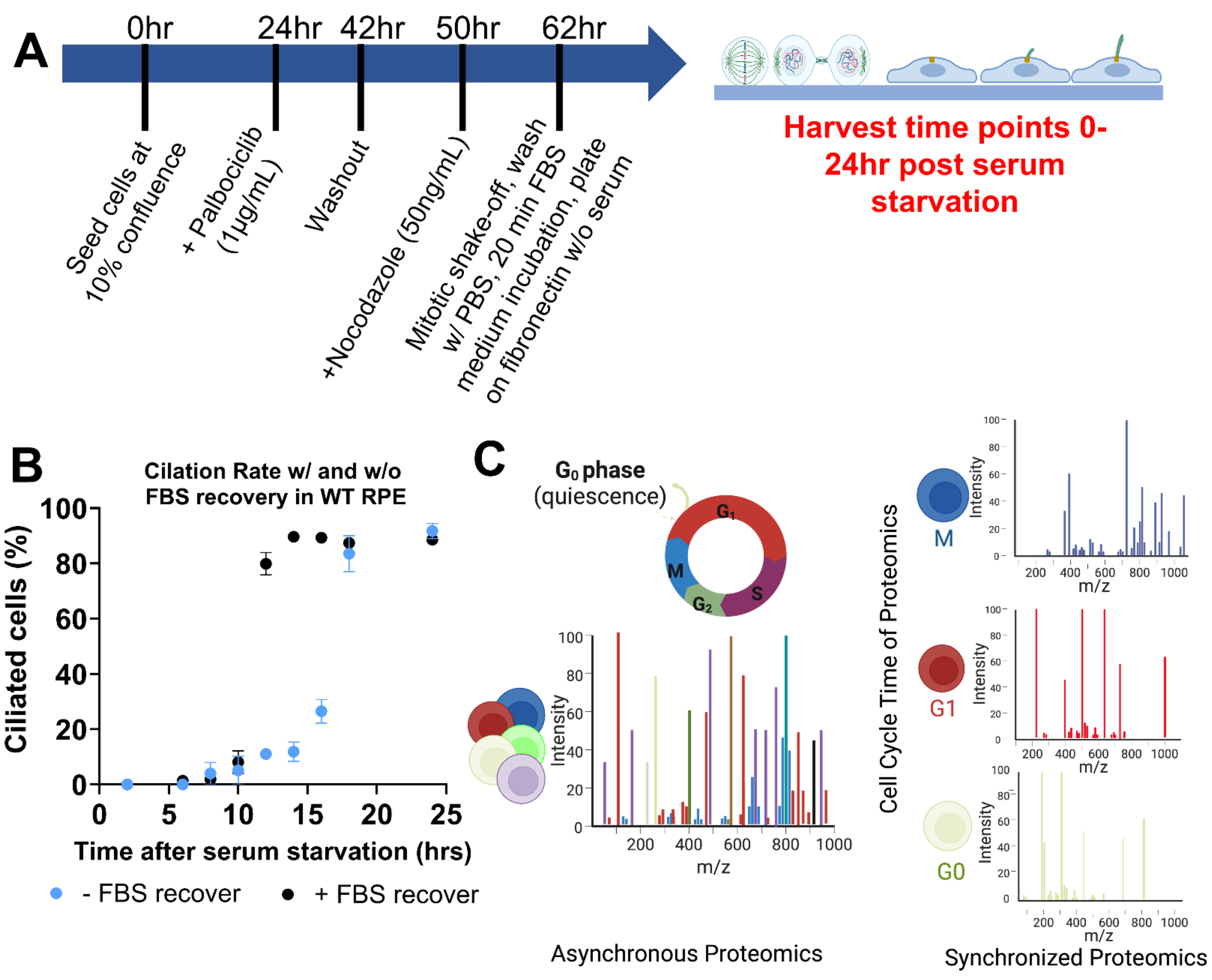

Fig. S1. Workflow of the STAMP synchronization protocol. (A) RPE and MEF cells were seeded at low density and were exposed to multiple different rounds of synchronization procedures before being harvested via mitotic shake-off. Schematic of the different stages captured by our synchronization protocol: metaphase onward through ciliogenesis and G_0 entry. (B) Quantification of ciliation of FBS-recovered versus non-FBS-recovered STAMP synchronized cells as a control for synchronization protocol. Experiments were performed in replicate, N=100 per replicate. Error bars = SEM. (C) Schematic illustrating the proof of principle for generating synchronized proteomics data, which incorporates the cellular time dimension and enriches for specific proteoforms that are either not detected or quantifiable in asynchronous proteomics.

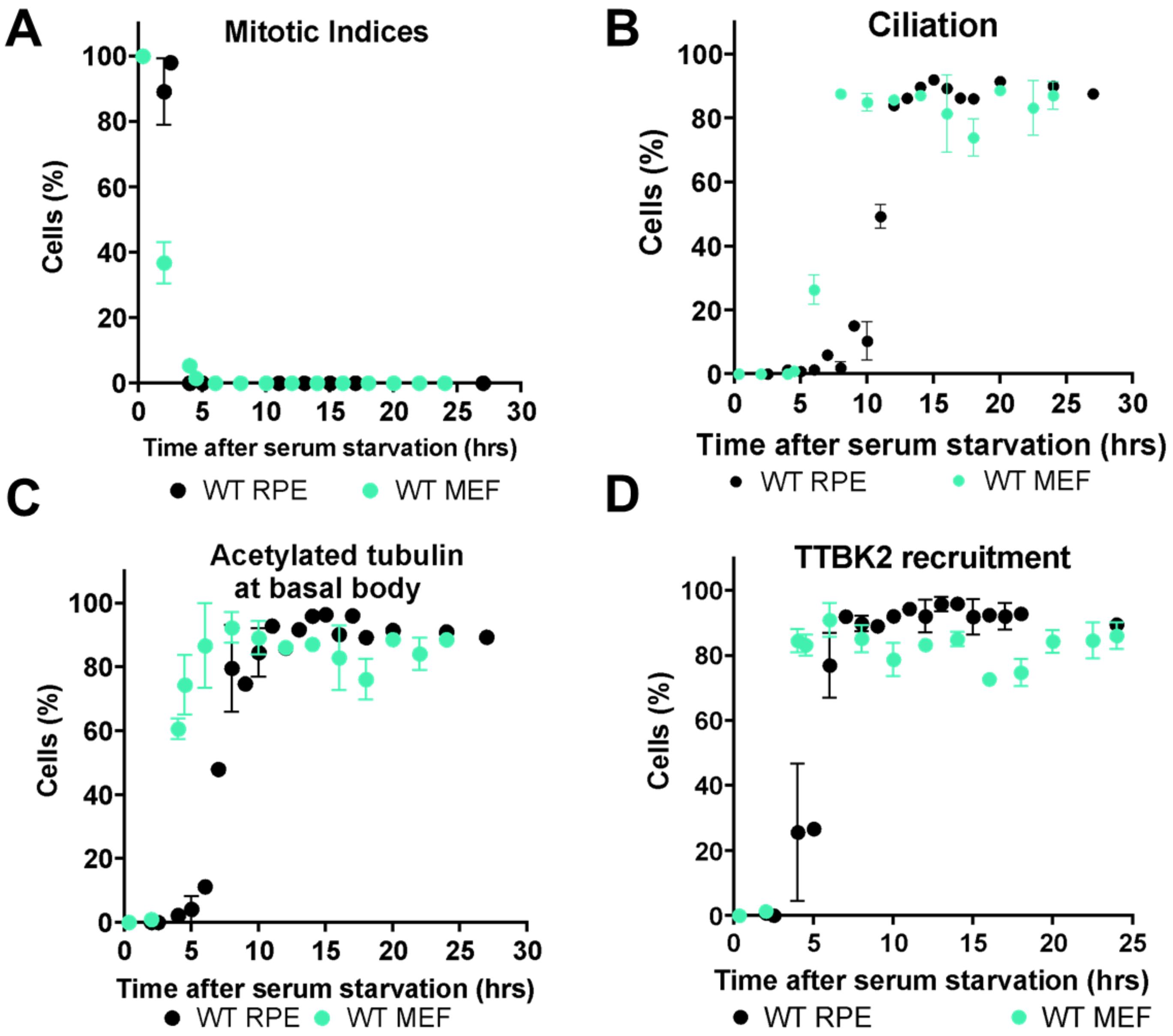

Fig. S2. Comparative STAMP of MEFs versus RPEs. Quantification of key markers of ciliogenesis/G_0 entry in RPEs versus MEFs via IF, including (A) mitotic indices, (B) ciliation, (C) acetylated tubulin at basal body, and (D) TTBK2 recruitment. Experiments were performed in replicate, N=100 per replicate. Error bars= SEM.

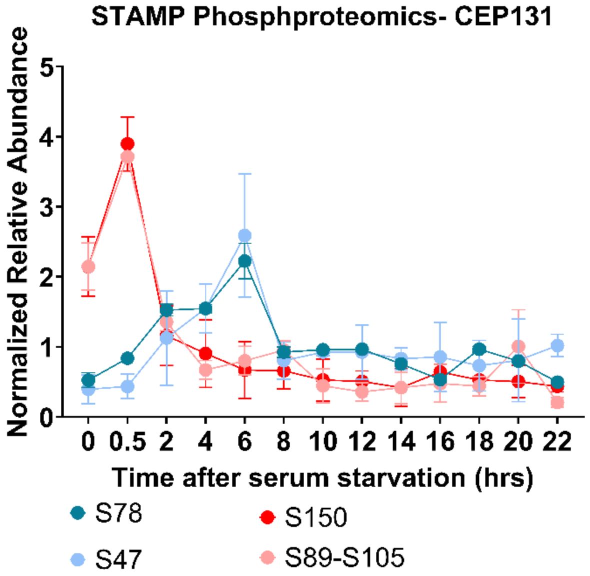

Fig. S3. STAMP phospho-proteomics identifies transient CEP131 phosphorylation sites. Plot of the normalized relative abundance of different CEP131 phosphorylations during G_0 progression. While proline-directed sites including [S150] and [S89-S105] are much more abundant during cell division, the N-terminal sites ([S47], and [S78]) are dynamically regulated during vesicle recruitment. Error bars = SD.

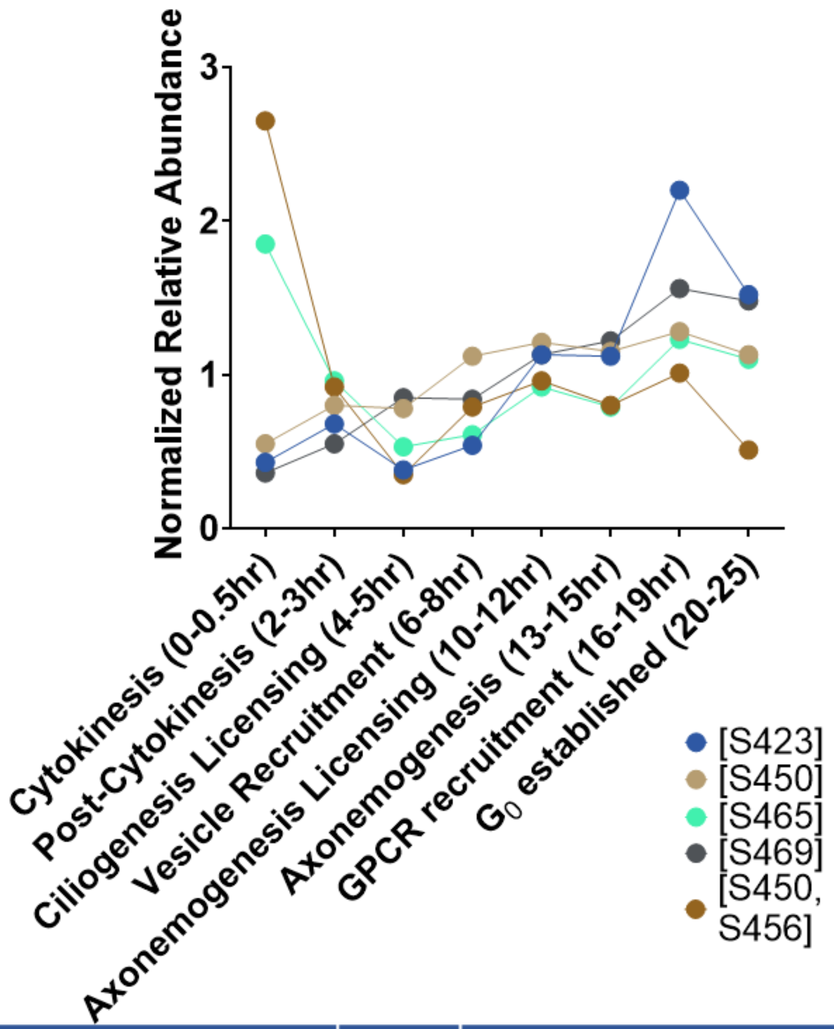

Site	Motif	Cluster	Predicted window of fxn	Putative Kinase
S423	GRAGPFSsSRCGASV	16	GPCR recruitment and loading	PKCA
S450	RIERNLQsPTQFQTP	2	gradual increase starting at 4,5hr	JNK
S450,S456	RIERNLQsPTQFQtP	7	high during cytokinesis	ERK
S465	RSSAIRRsGSTSPLG	7	high during cytokinesis	PAK
S469	IRRSGSTsPLGFARA	2	gradual increase starting at 4,5hr; Known to induce protein degradation	PBK

Fig. S4. Representative example of how to read phosphosignature maps using ULK1. Above, example plot of the average normalized intensity of 5 different phosphosites of ULK1 over time as generated via STAMP phosphoproteomics. Below, a chart indicating the phosphosite, its corresponding motif, putative kinases that are strong candidates for making that modification (based on the Cantley Kinase Metric), and predicted function.

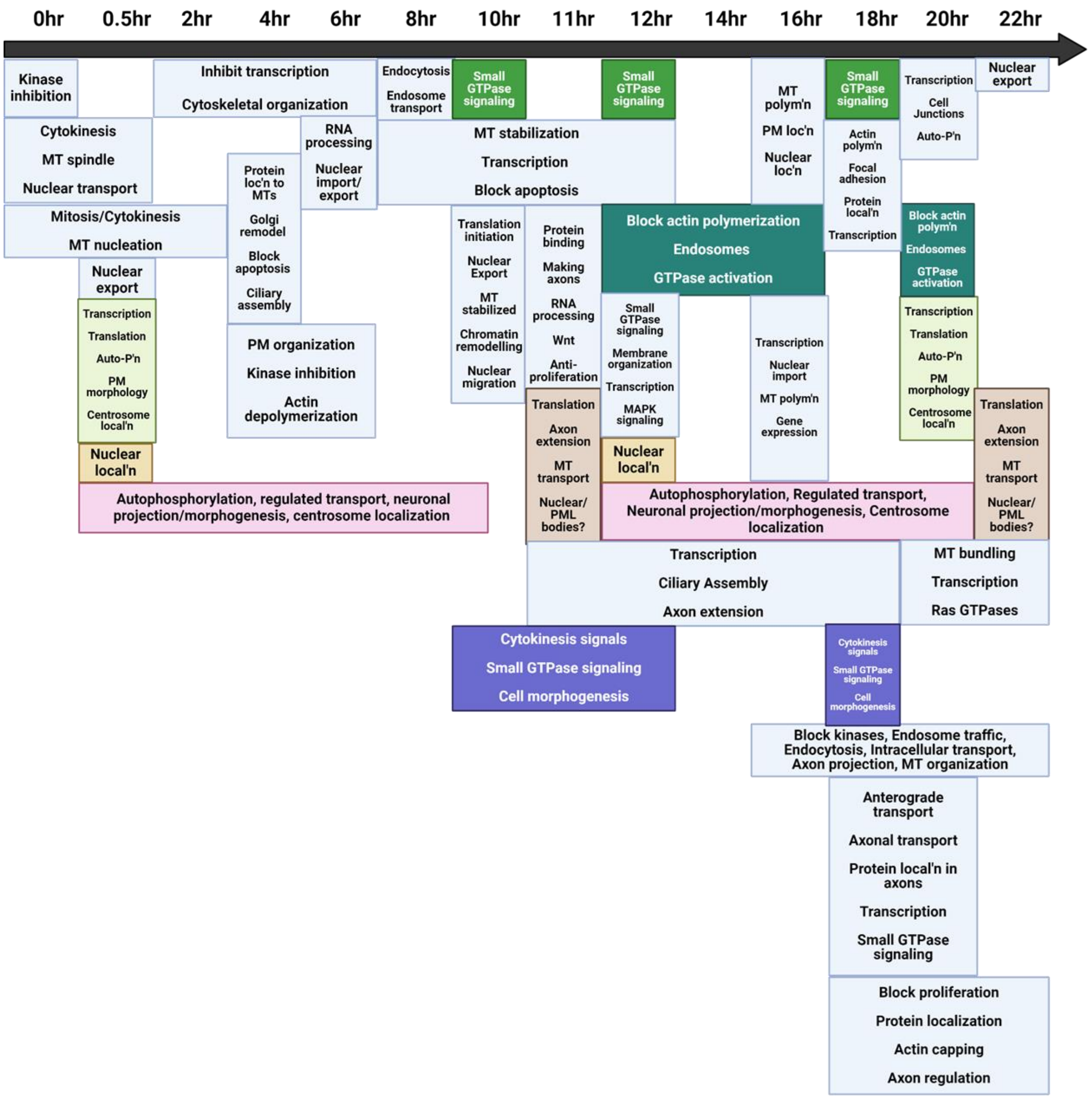

Fig. S5. Gene Ontology Mapping. Schematic summarizing the Gene Ontology analysis via EnrichR (https://maayanlab.cloud/Enrichr/). Individual clusters generated from phospho-proteomics (see Fig.5) were run through the software, compared with the GO Biological Process 2023 analysis, and plotted via Bar Graphs and Clustergram. The top 4-5 functions for each cluster were mapped to the timeline as an unbiased approach to observe what functions are active at different time periods during G_0 entry.

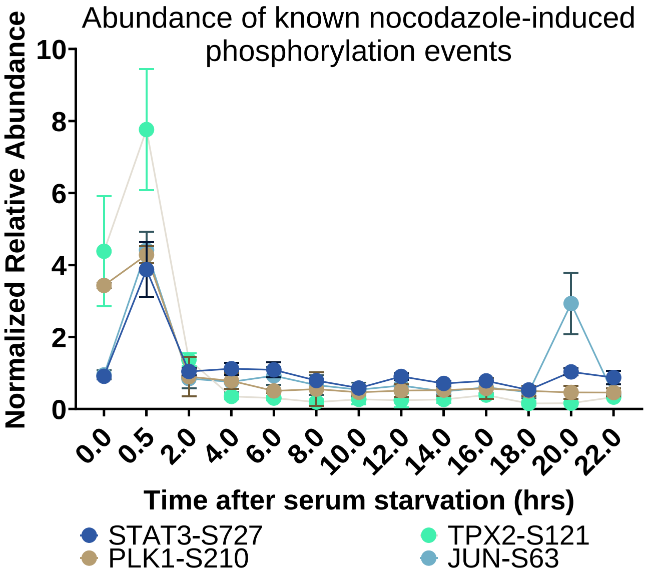

Fig. S6. Abundance of known nocodazole-induced phosphorylations. Plot of the intensities of 4 different phosphosites over time that previous groups have shown are signatures of nocodazole treatment, as generated from STAMP phosphoproteomics. Error bars = SD.

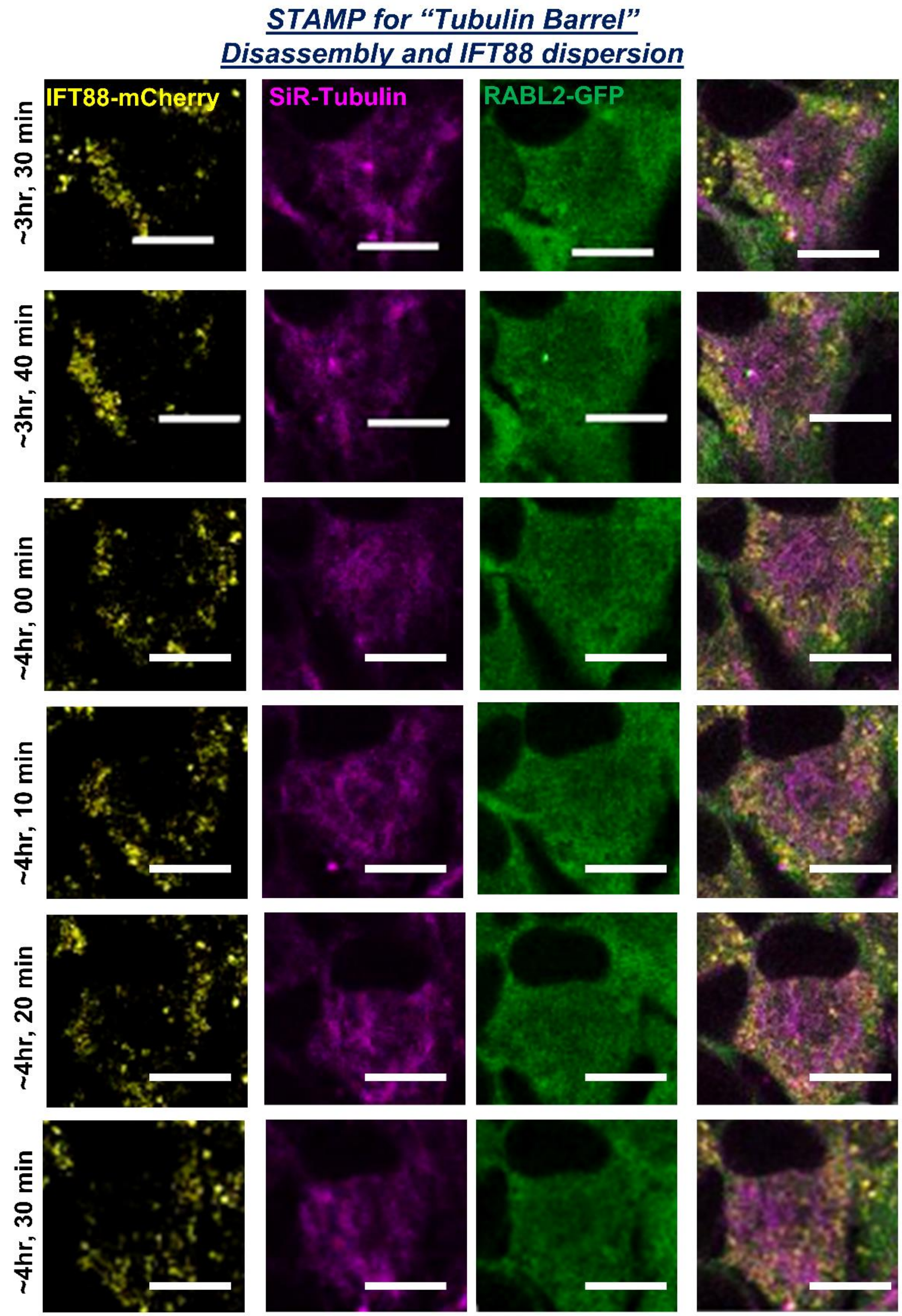

Fig. S7. STAMP for "Tubulin Barrel" Disassembly and IFT88 dispersion. Representative timelapse images reflecting the disassembly of the tubulin barrel, as tracked via live cell imaging at 40x magnification via confocal microscopy (z-stacks, every 2.5min). RABL2-LAP (Green) + IFT88-mCherry (yellow) cells were synchronized via STAMP and stained for SiR-Tubulin (magenta). Scale bar = 20μm.

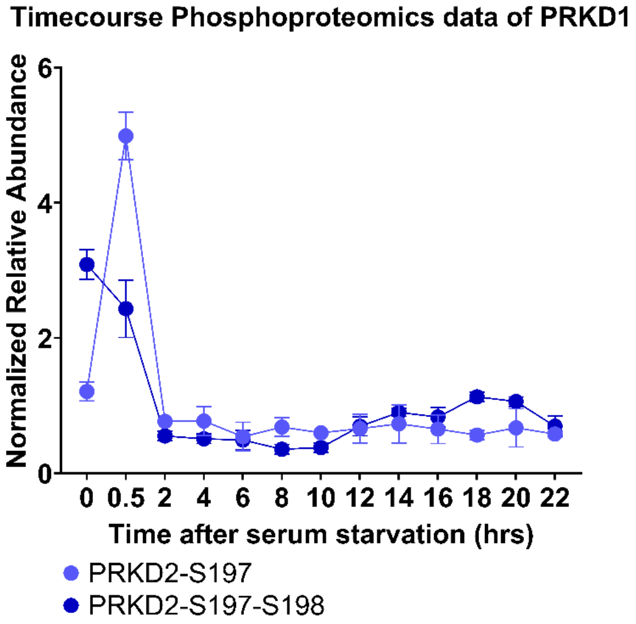

Fig. S8. Abundance of identified phosphorylation sites on PRKD2. Plot of the intensities of monophosphorylated [S197] and doubly phosphorylated [S197, S198] over time based on STAMP phosphoproteomics of WT RPE. Error bars = SD.

Synchronized Temporal-spatial Analysis via Microscopy and Phospho-proteomics (STAMP) of Quiescence

Data S1.

Collision Activated Dissociation (CAD) MS2 Scans of each plotted individual phosphorylation sites discussed in the paper

AMBRA1_S1205

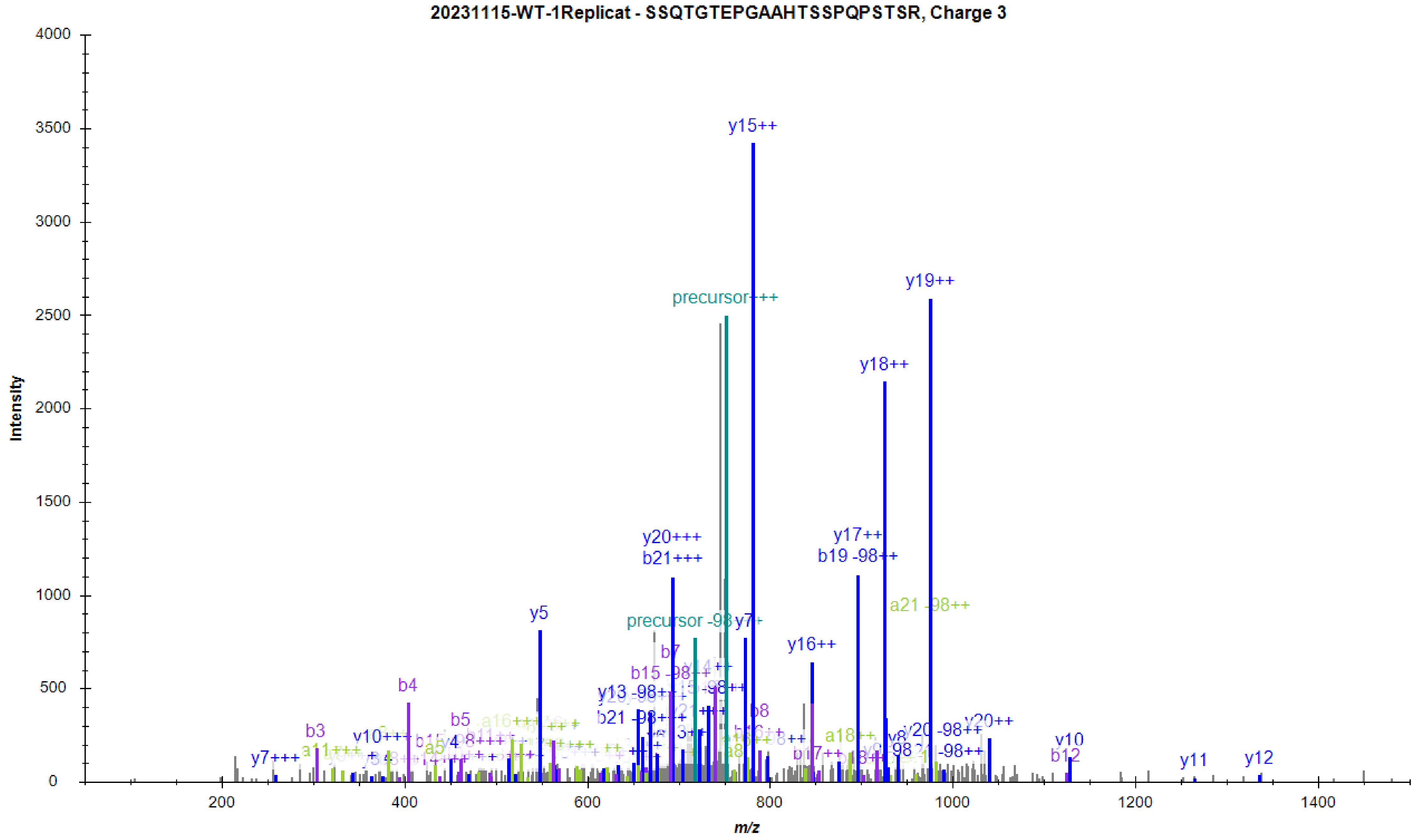

AMBRA1_S1227

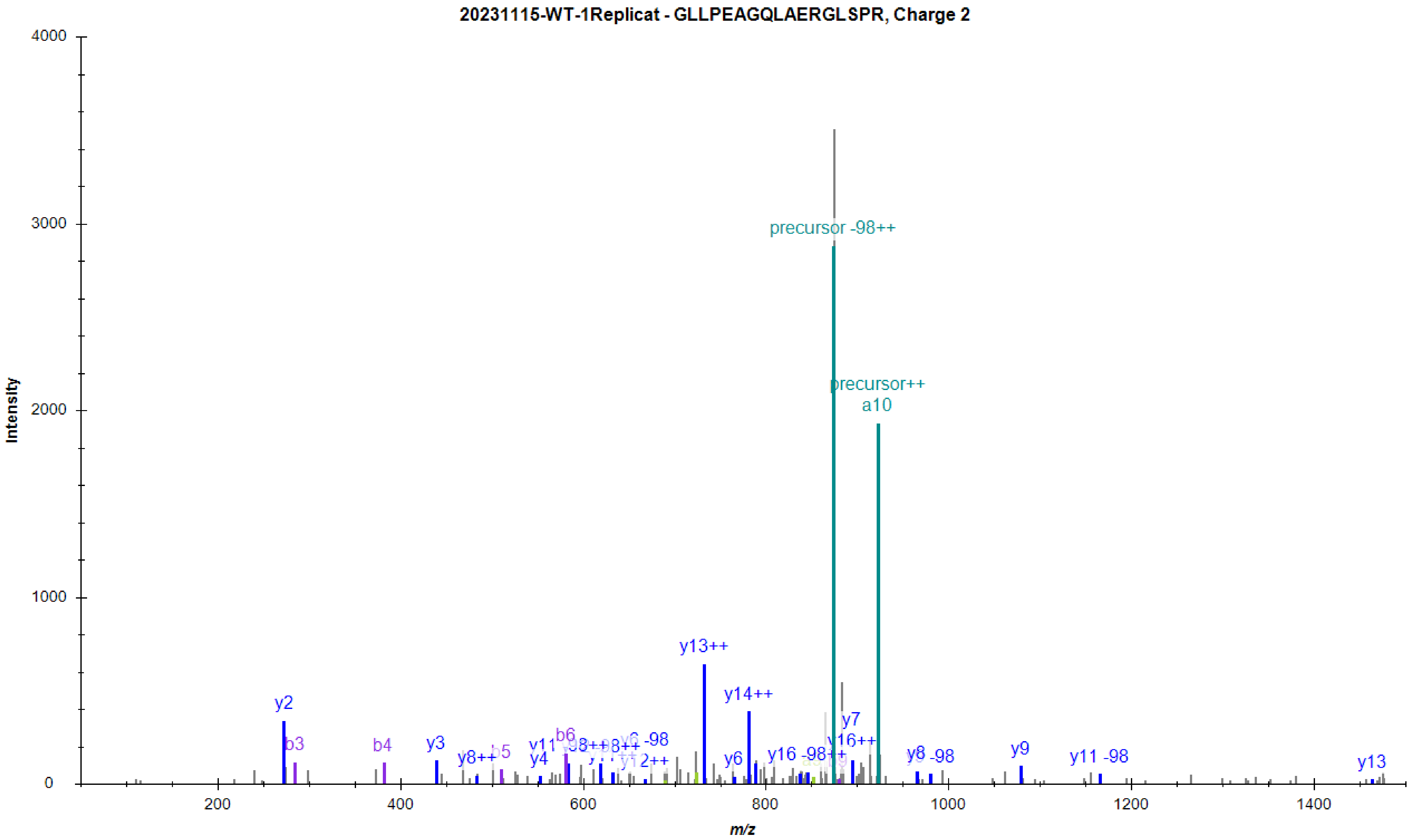

ATG16L1_S269

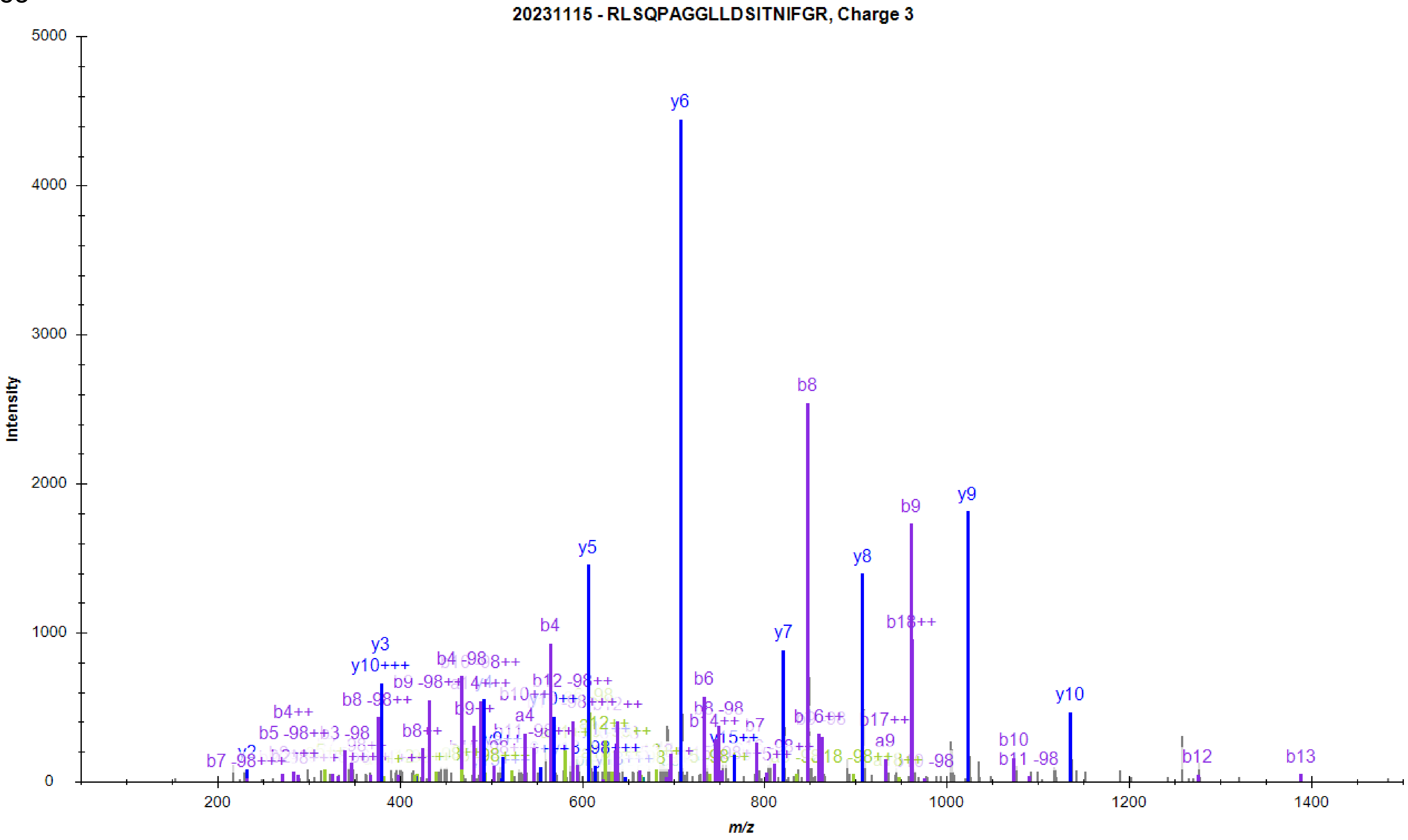

ATG16L1_S278

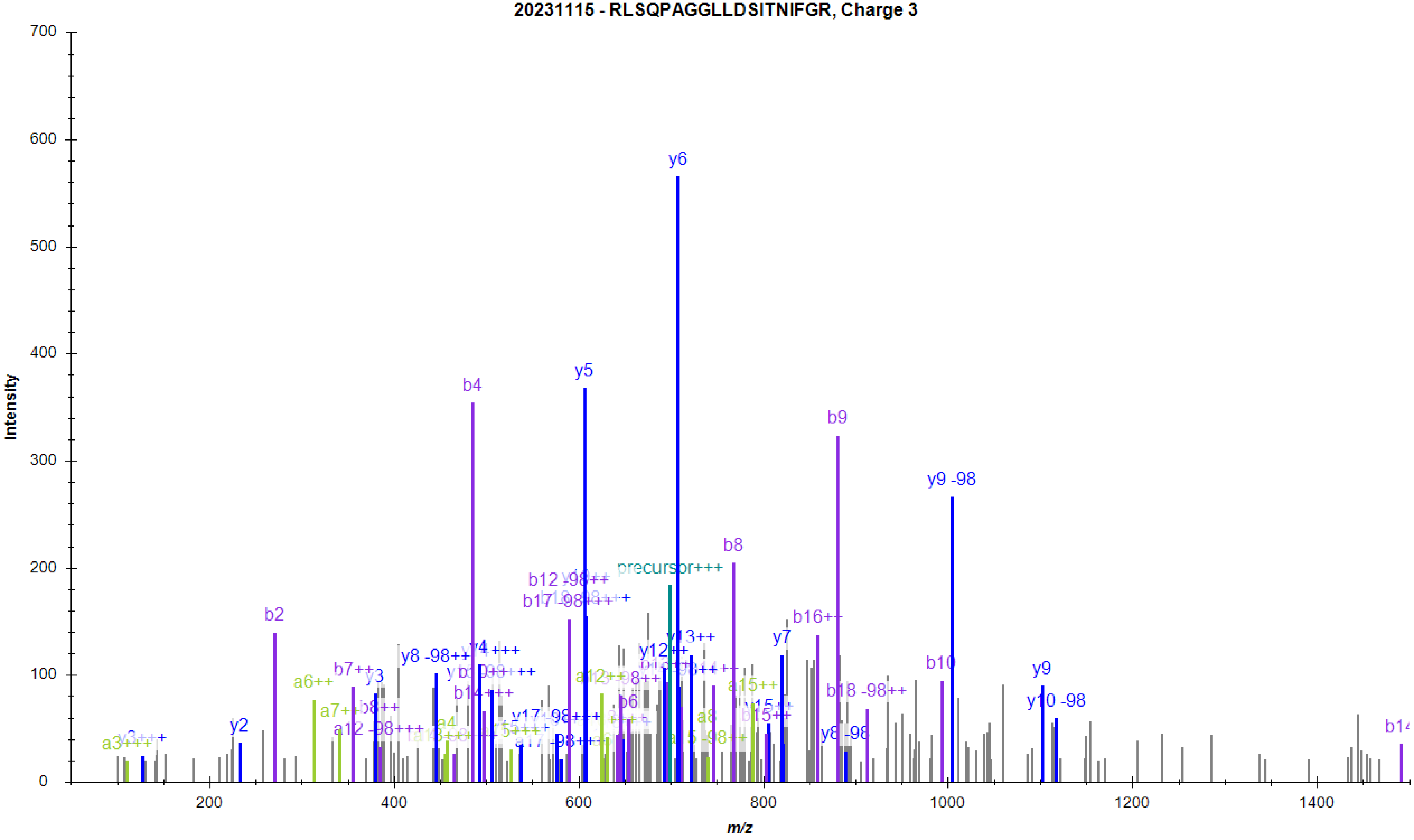

Bcl-10-S138

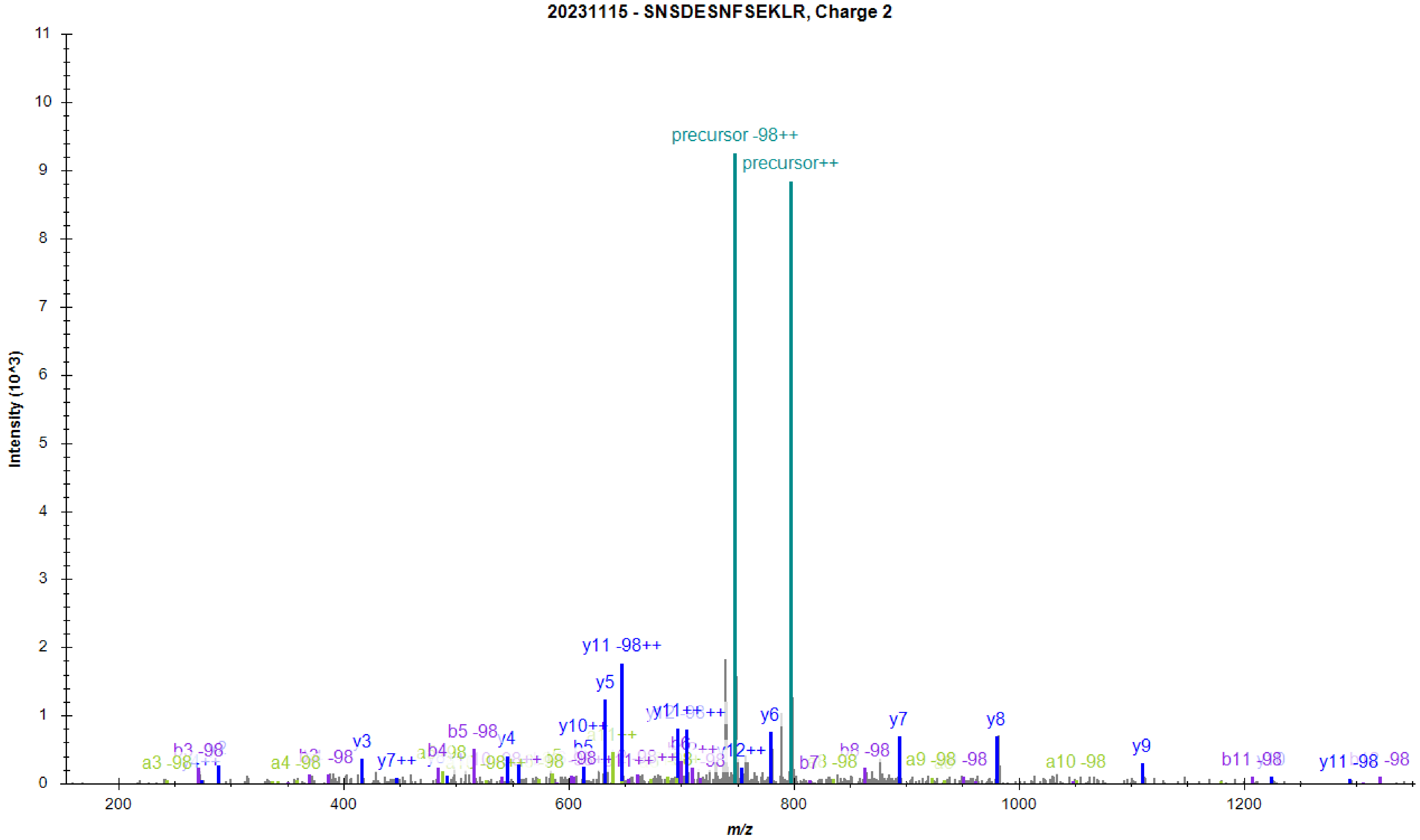
20231115 - SNSDESNFSEKLR, Charge 2
Intensity (10^3)
m/z
precursor -98++
precursor++
y11 -98++
y5
y6
y7
y8
y9
y3
y4
y7++
y10++
b5 -98
b3 -98
a3 -98
a4 -98
b8 -98
a9 -98
a10 -98
b11 -98
y11 -98

CCP110-S372

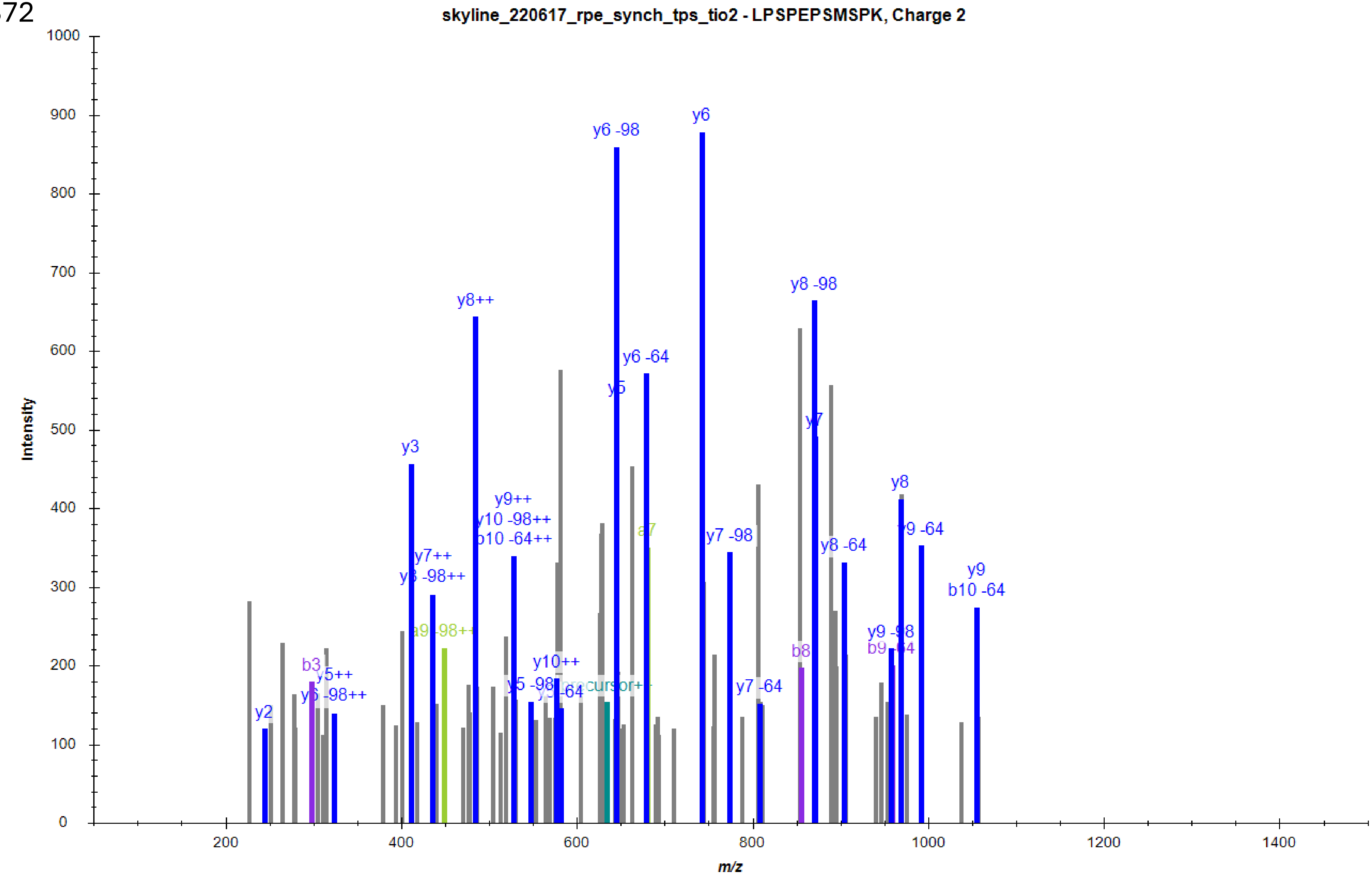
skyline_220617_rpe_synch_tps_tio2 - LPSPEPSMSPK, Charge 2
Intensity
m/z
y6
y6 -98
y8++
y8 -98
y6 -64
y3
y9++
y10 -98++
b10 -64++
y7++
y7 -98
y8 -64
y8
y9
b10 -64
y10++
y7 -64
b8
b3
y5++
y2
a7

CEP131-S78

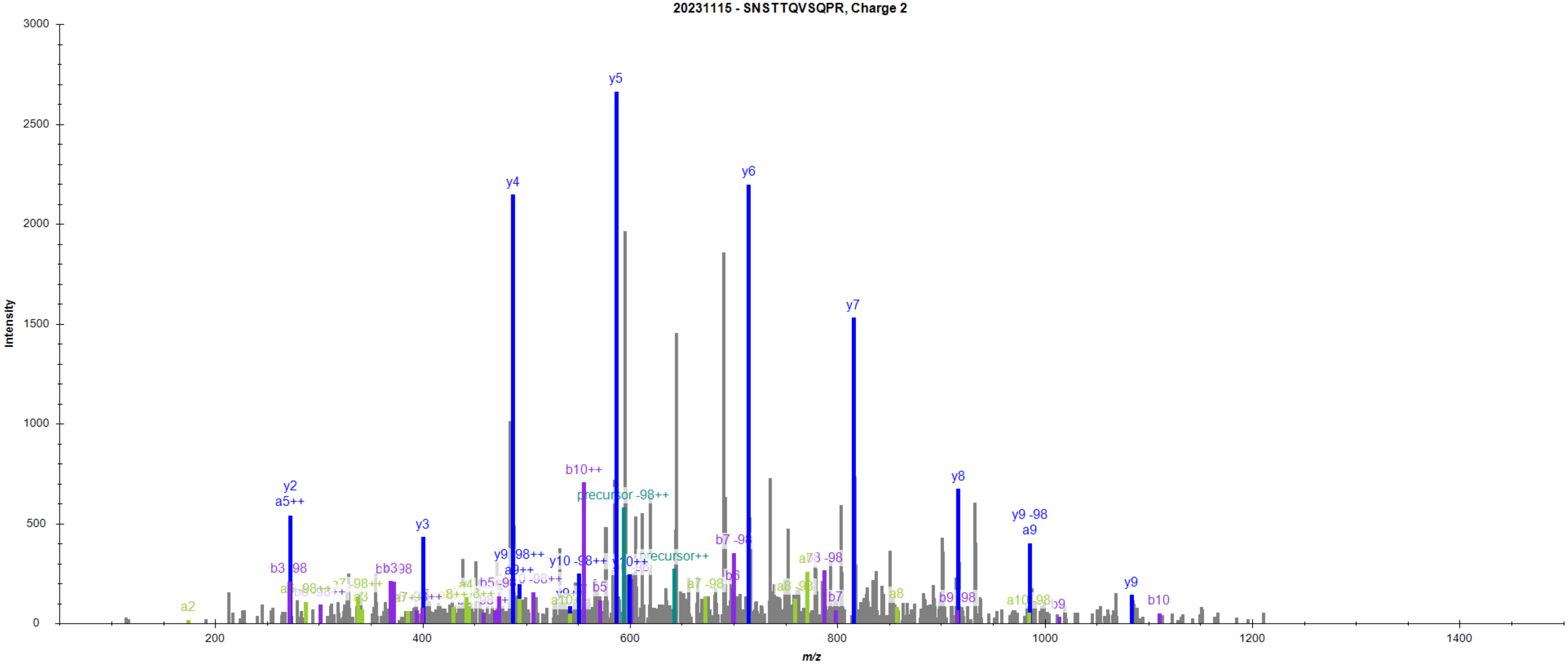

20231115 - SNSTTQVSQPR, Charge 2
Intensity
m/z
y5
y6
y4
y7
y8
y9 -98
a9
y9
b10
b10++
precursor -98++
precursor++
y2
a5++
y3
b7 -98
a2

CEP131-S47

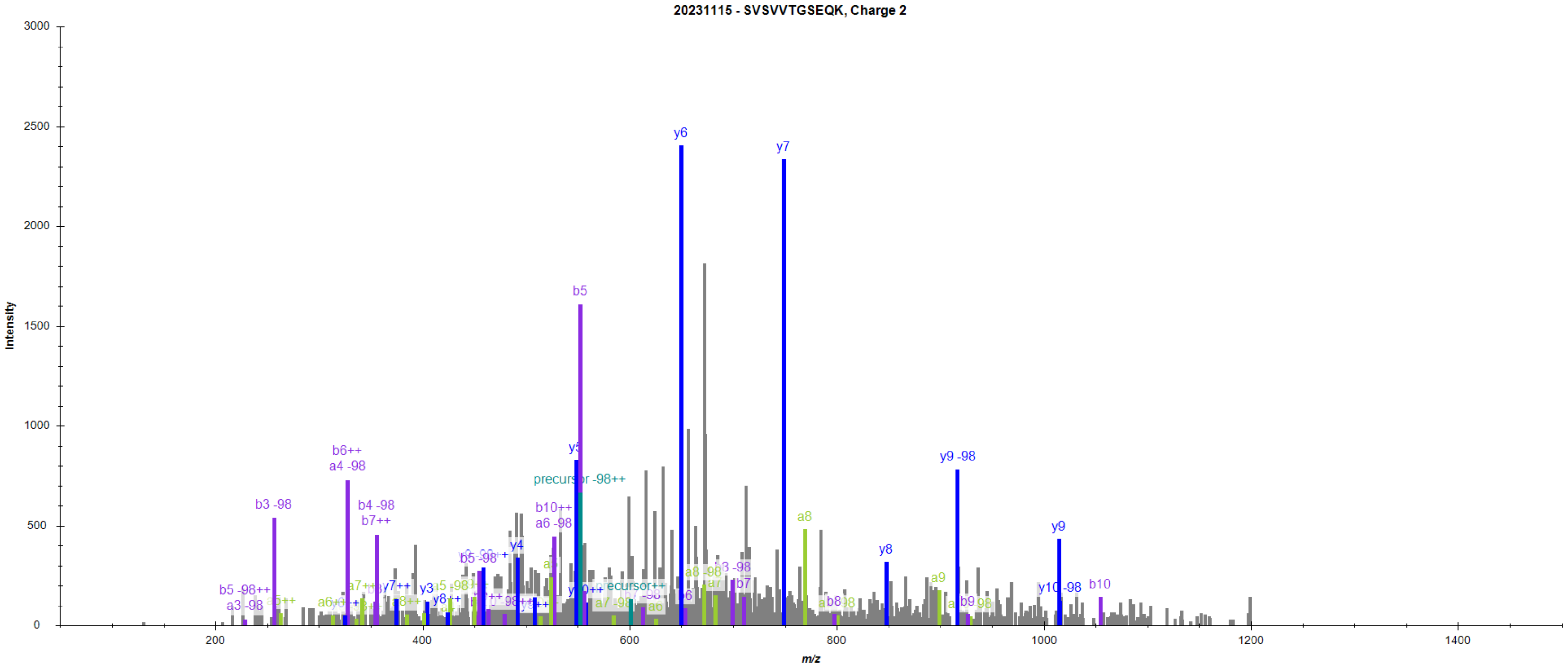

20231115 - SVSVVTGSEQK, Charge 2
Intensity
m/z
y6
y7
b5
y5
precursor -98++
y9 -98
y9
y8
a8
b10
y10 -98
b3 -98
b6++
a4 -98
b4 -98
b7++
b10++
a6 -98
y4
a9
b9
b7
b8

CEP131-S150

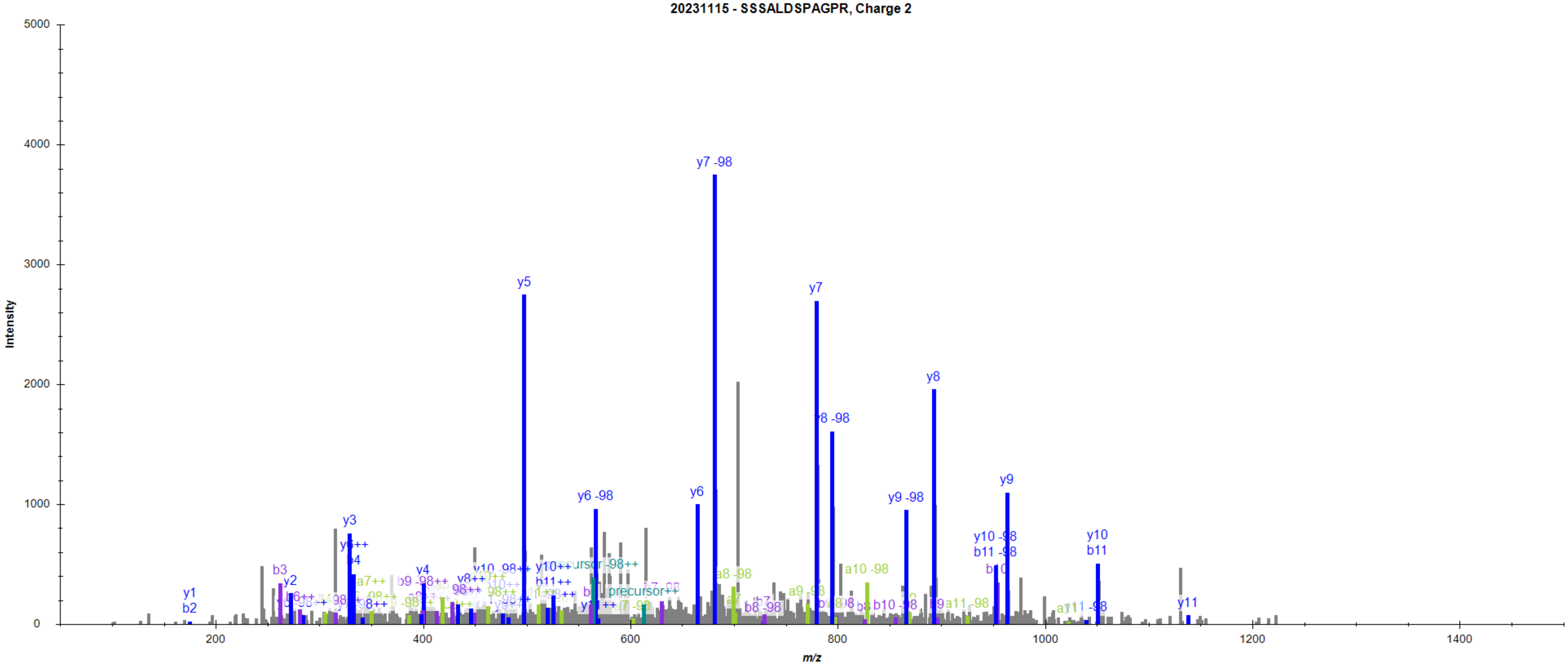
20231115 - SSSALDSPAGPR, Charge 2
Intensity
m/z
0
1000
2000
3000
4000
5000
200
400
600
800
1000
1200
1400
y7 -98
y5
y7
y8
y8 -98
y6 -98
y6
y9 -98
y9
y10 -98
b11 -98
y10
b11
y3
y4
b3
y2
y1
b2
y11
a10 -98
a8 -98
a9 -98
a11 -98
b10 -98
b8 -98
y10++
b11++
precursor++
a7++
b9 -98++
v8++

ELYS_S1541

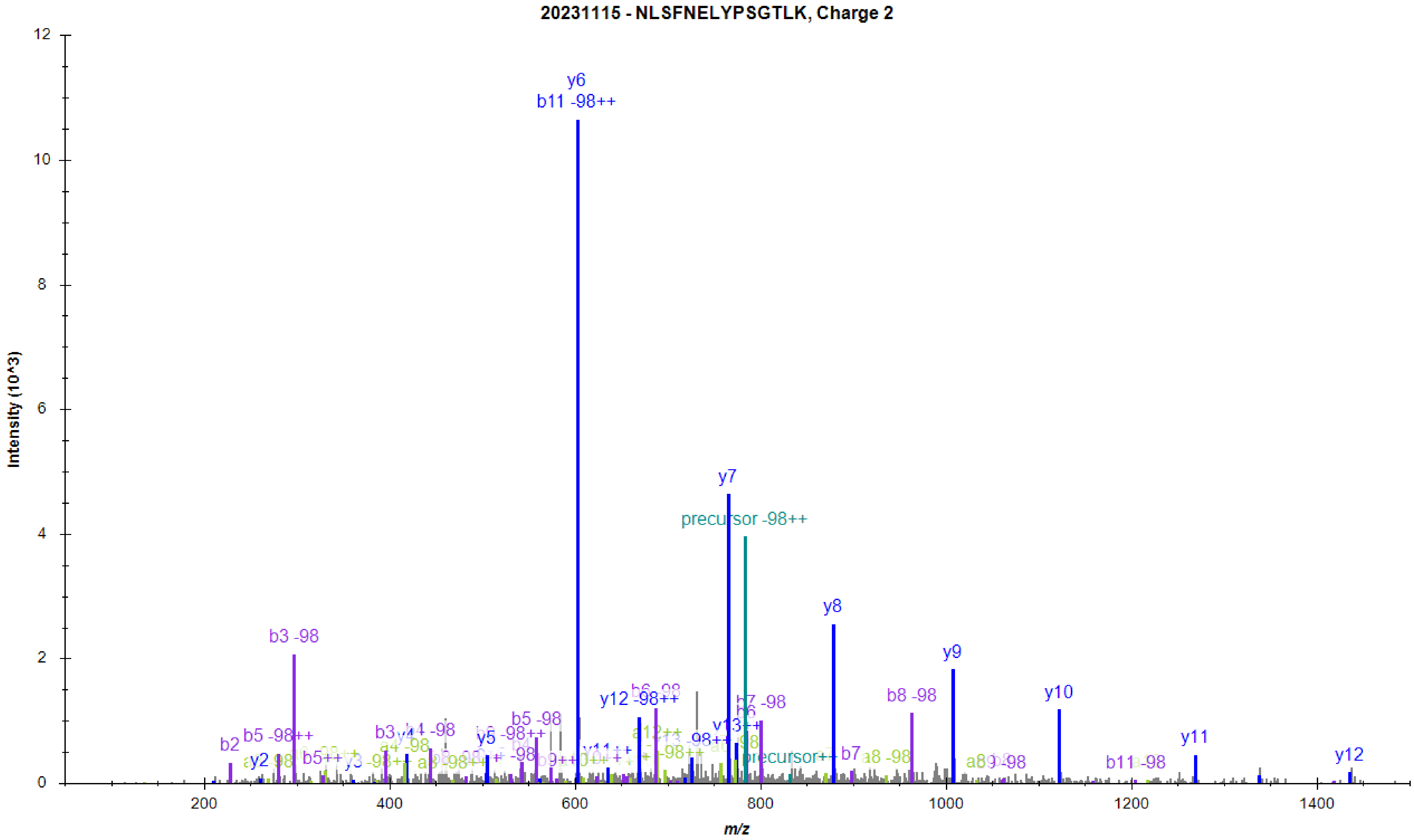
20231115 - NLSFNELYPSGTLK, Charge 2
Intensity (10^3)
m/z
y6
b11 -98++
y7
precursor -98++
y8
y9
y10
y11
y12
b3 -98
b8 -98
b2
y2
y5
b5 -98
y12 -98++
b7
b11 -98

ELYS_S2212

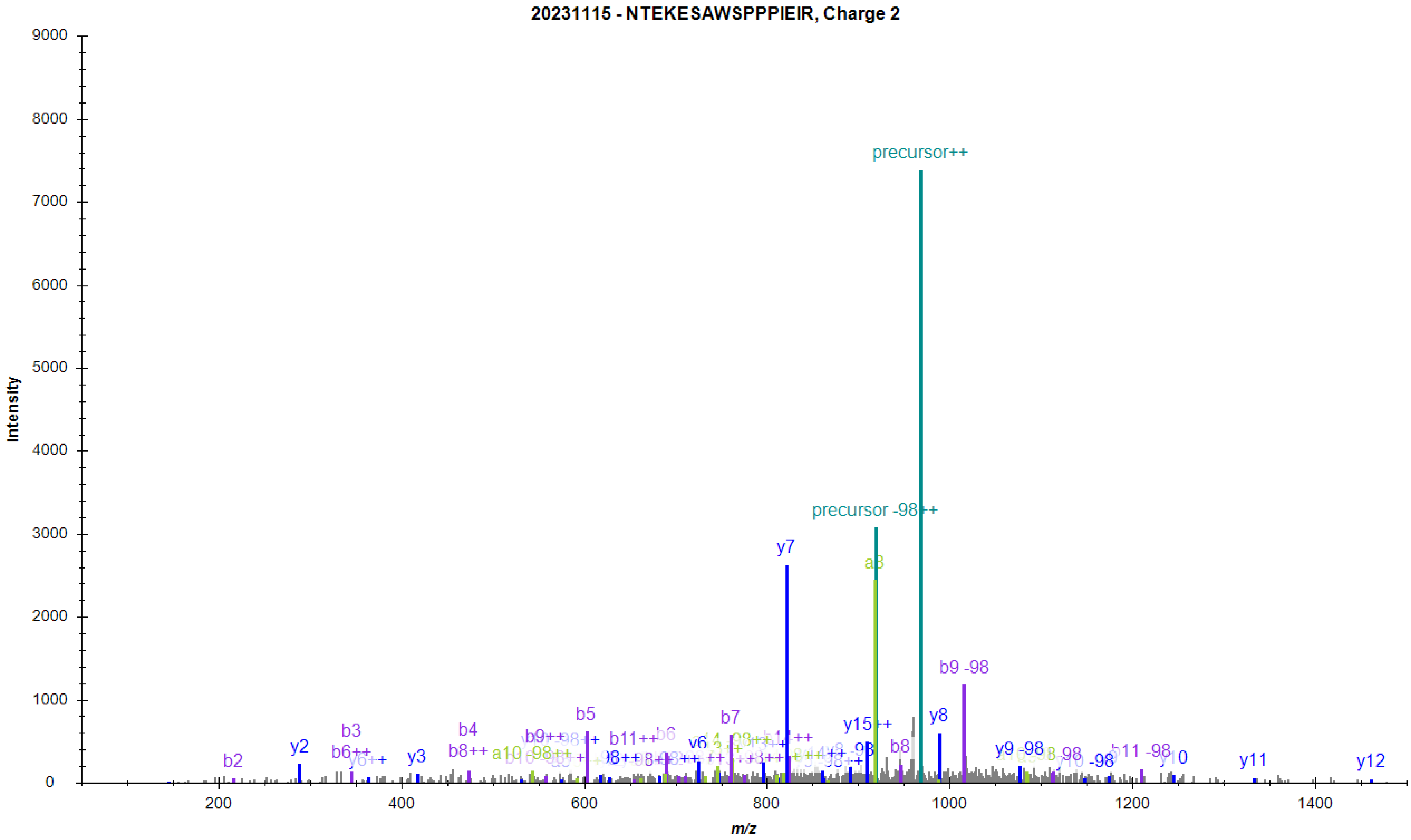
20231115 - NTEKESAWSPPPIEIR, Charge 2
Intensity
m/z
precursor++
precursor -98++
y7
b9 -98
y8
b5
b7
b3
b4
y2
y3
b2
y11
y12
0
1000
2000
3000
4000
5000
6000
7000
8000
9000
200
400
600
800
1000
1200
1400

ELYS_S2222-S2226

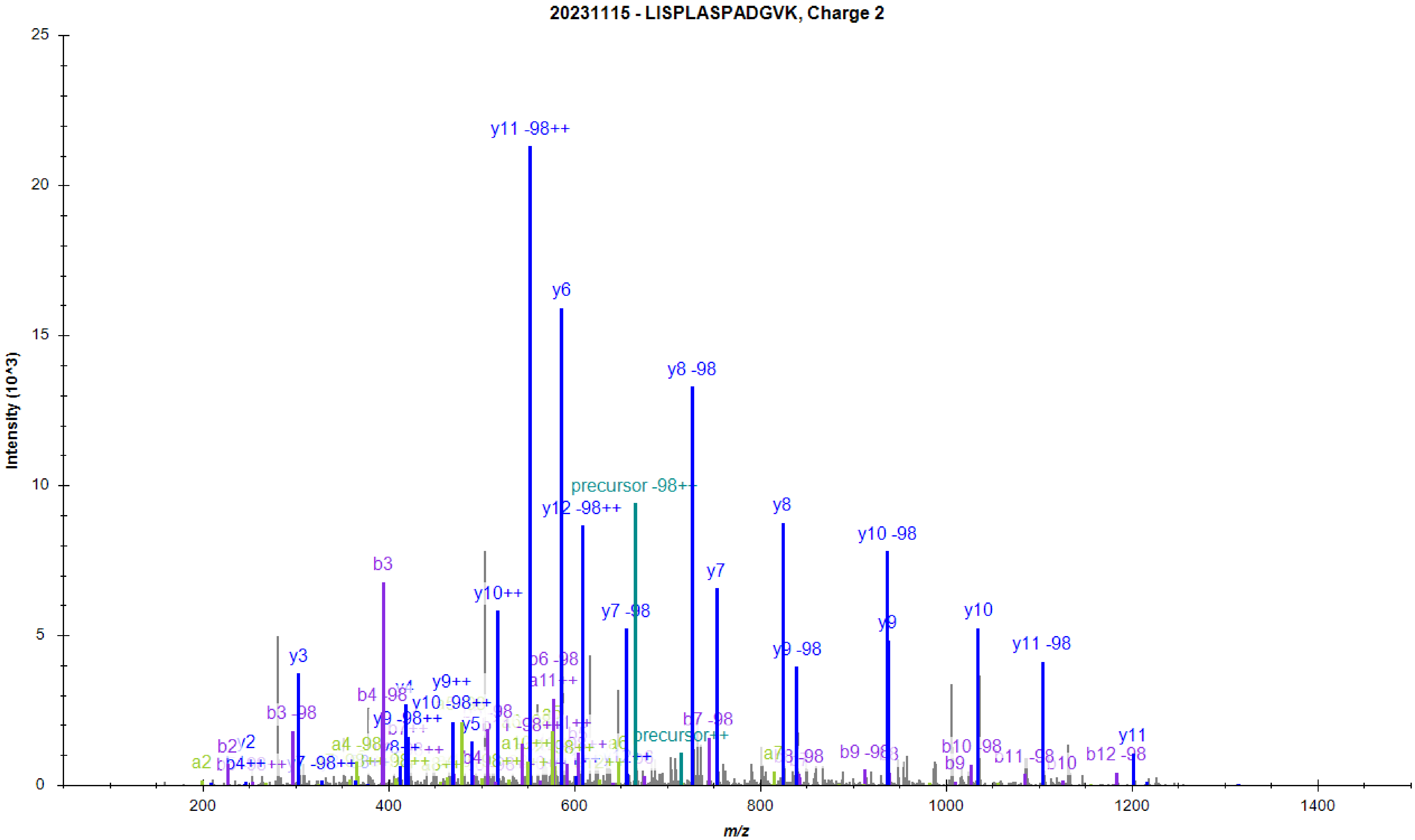

ELYS_T1808

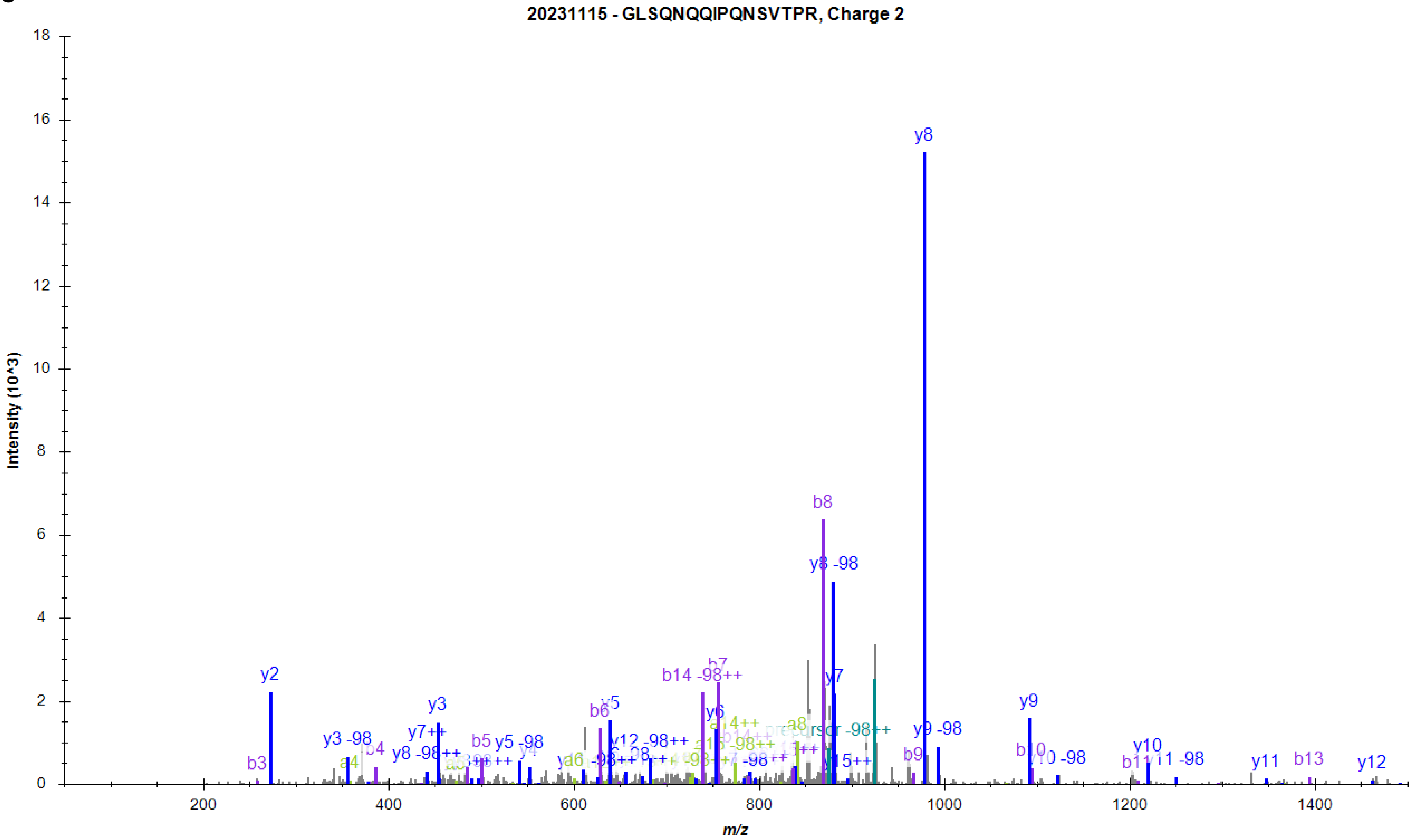
20231115 - GLSQNQQIPQNSVTPR, Charge 2
Intensity (10^3)
m/z
0
2
4
6
8
10
12
14
16
18
200
400
600
800
1000
1200
1400
y8
b8
y8 -98
y2
y3
y7
y9
b14 -98++
y3 -98
b3
b4
y7++
y8 -98++
b5
y5 -98
b6
y5
y6
y12 -98++
y9 -98
b9
y10
y10 -98
b11
y11 -98
y11
b13
y12
a4
a8
precursor -98++
y15++

FLCN-S62

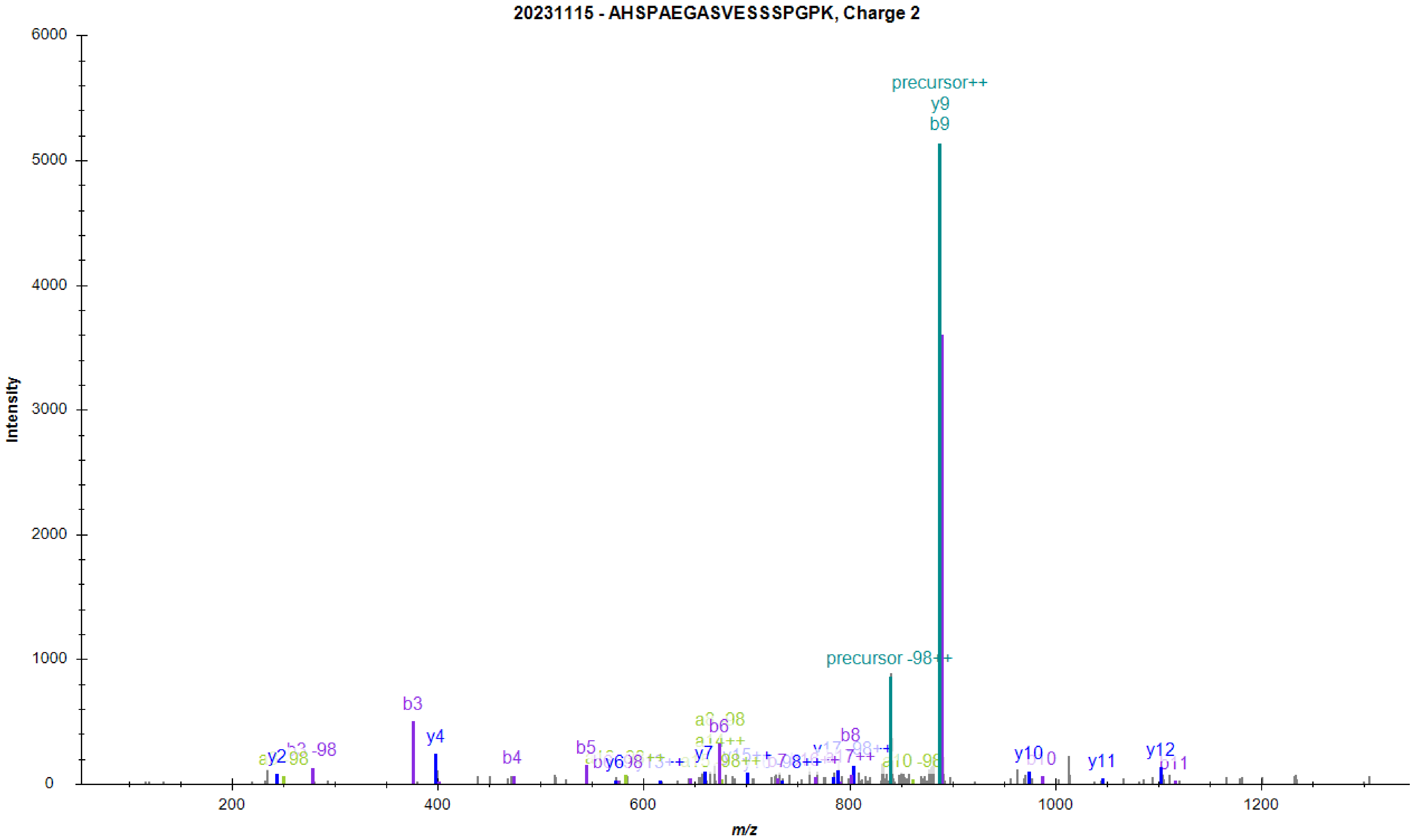
20231115 - AHSPAEGASVESSSPGPK, Charge 2
Intensity
m/z
6000
5000
4000
3000
2000
1000
0
200
400
600
800
1000
1200
precursor++
y9
b9
precursor -98++
b3
y4
b4
b5
y2
y7
b6
b8
y10
y11
y12

IFT22-S137

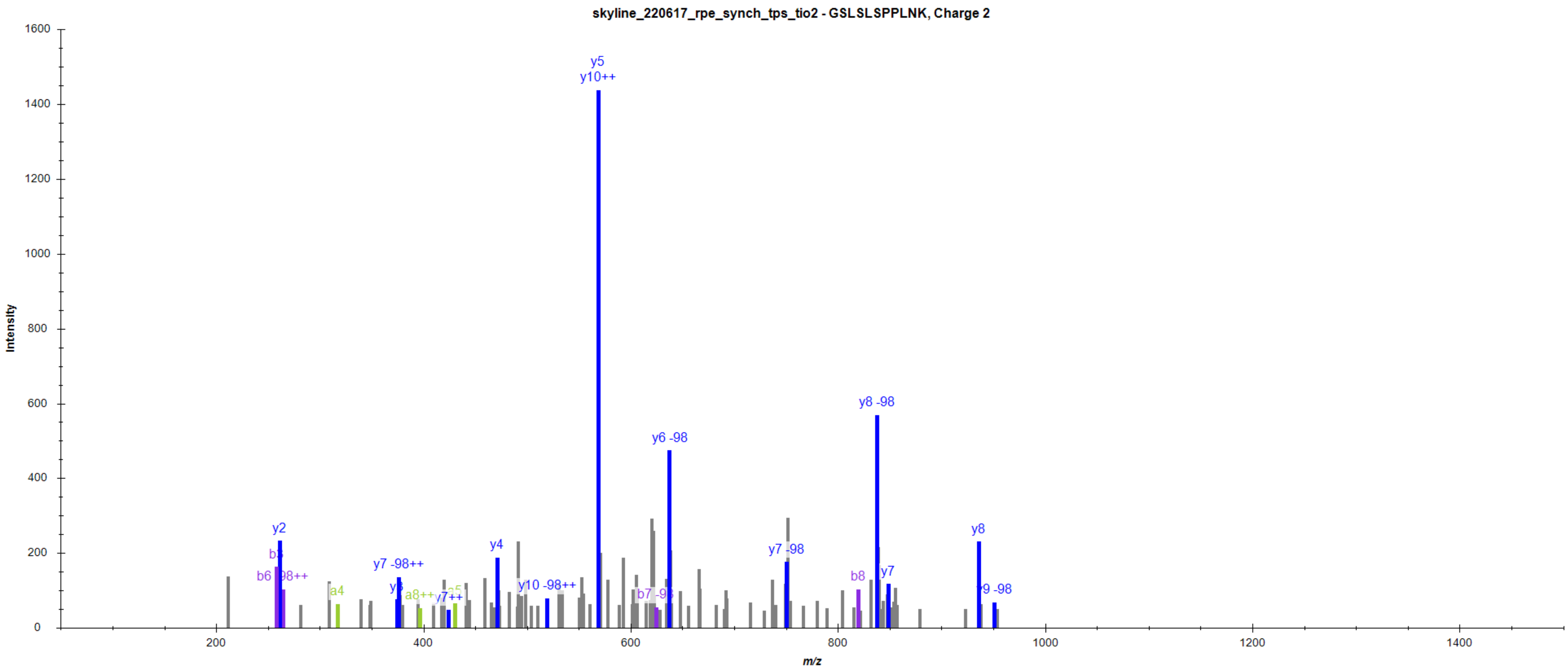

skyline_220617_rpe_synch_tps_tio2 - GSLSLSPPLNK, Charge 2
Intensity
m/z
y5
y10++
y6 -98
y8 -98
y2
y4
y7 -98++
y10 -98++
y7 -98
y8
b8
y7
a4
a8++
y7++

IFT43-S78

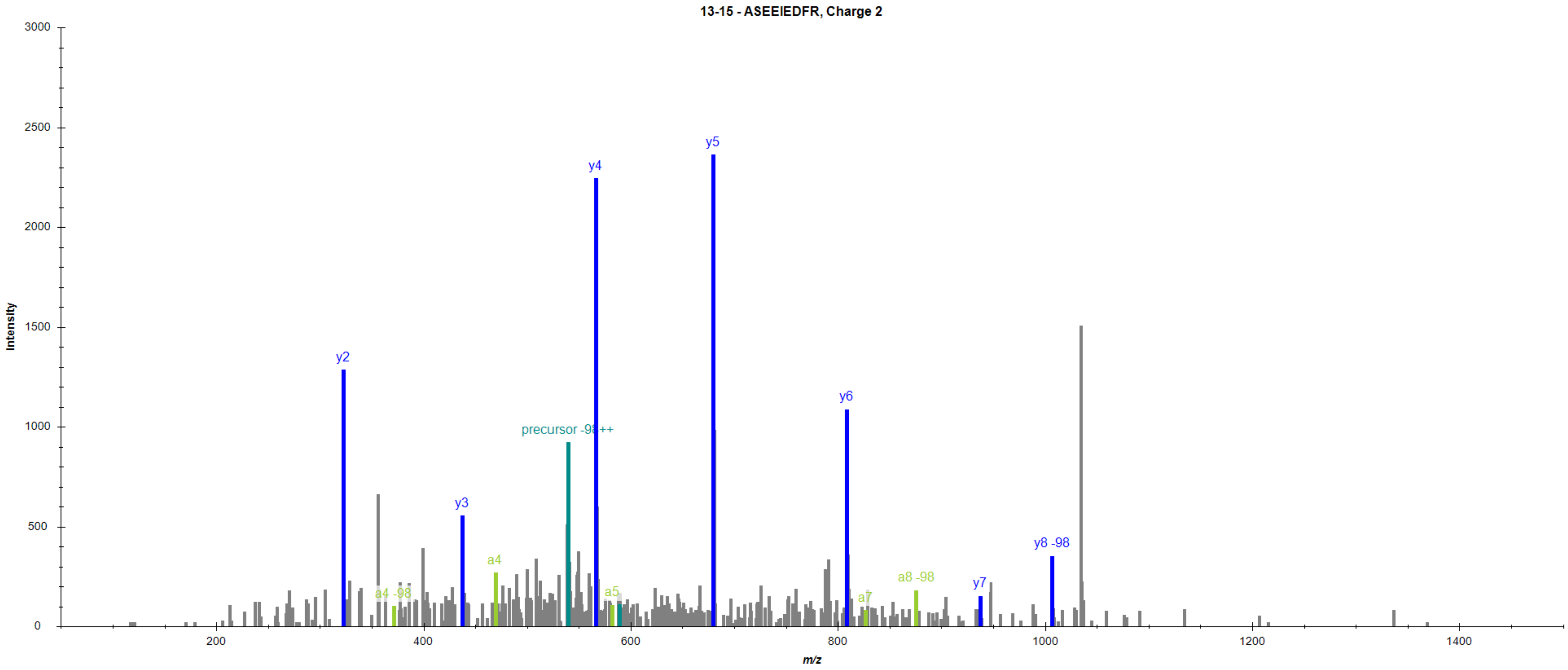

13-15 - ASEEIEDFR, Charge 2
Intensity
m/z
y2
y3
y4
y5
y6
y7
y8 -98
a4
a4 -98
a5
a7
a8 -98
precursor -98++
0
500
1000
1500
2000
2500
3000
200
400
600
800
1000
1200
1400

LAMTOR1_S27

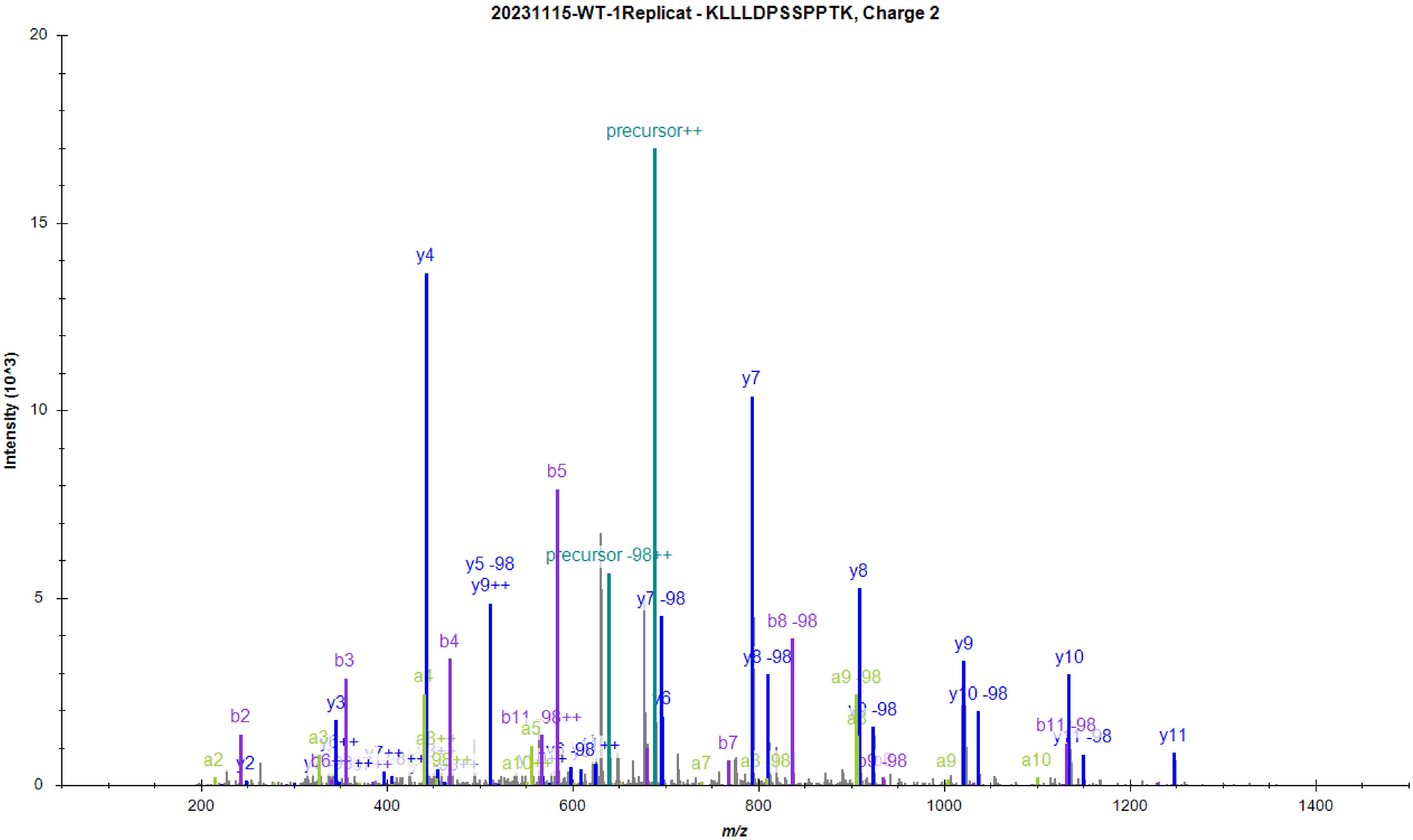
20231115-WT-1Replicat - KLLLDPSSPPTK, Charge 2
Intensity (10^3)
20
15
10
5
0
m/z
200
400
600
800
1000
1200
1400
precursor++
y4
y7
b5
precursor -98++
y5 -98
y9++
y7 -98
b8 -98
y8
y8 -98
y9
y10
y10 -98
a9 -98
b4
b3
a4
y3
b2
y2
a2
a5
b7
a7
a9
a10
y11
b11 -98
y11 -98
b11 -98++

NUP35_S73

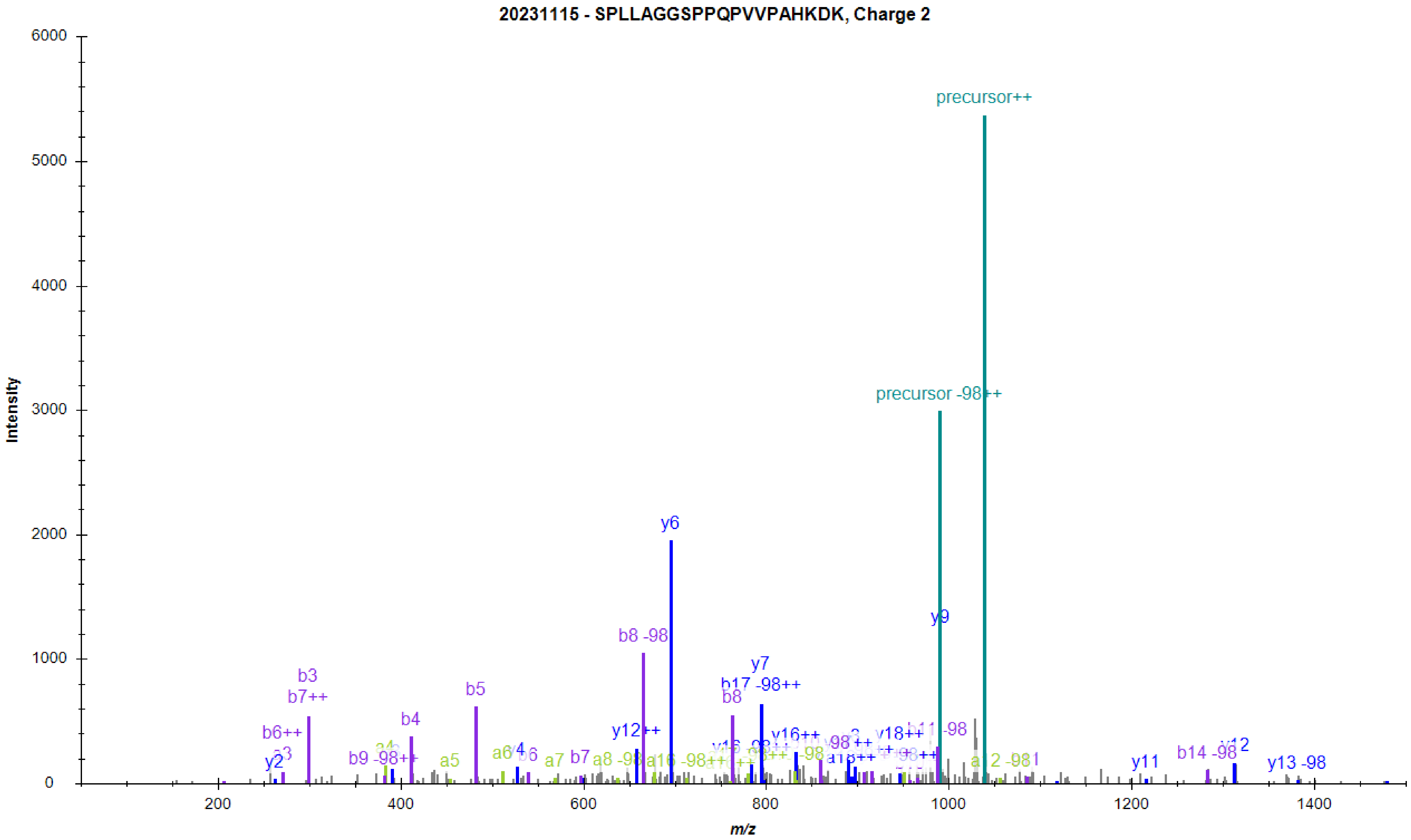

20231115 - SPLLAGGSPPQPVVPAHKDK, Charge 2
Intensity
m/z
0
1000
2000
3000
4000
5000
6000
200
400
600
800
1000
1200
1400
precursor++
precursor -98++
y6
y9
b8 -98
y7
b17 -98++
b8
b3
b7++
b5
b4
b6++
y2
b9 -98++
a5
a7
b7
y12++
y16++
y18++
y11
b14 -98
y13 -98

NUP35_S100_T106

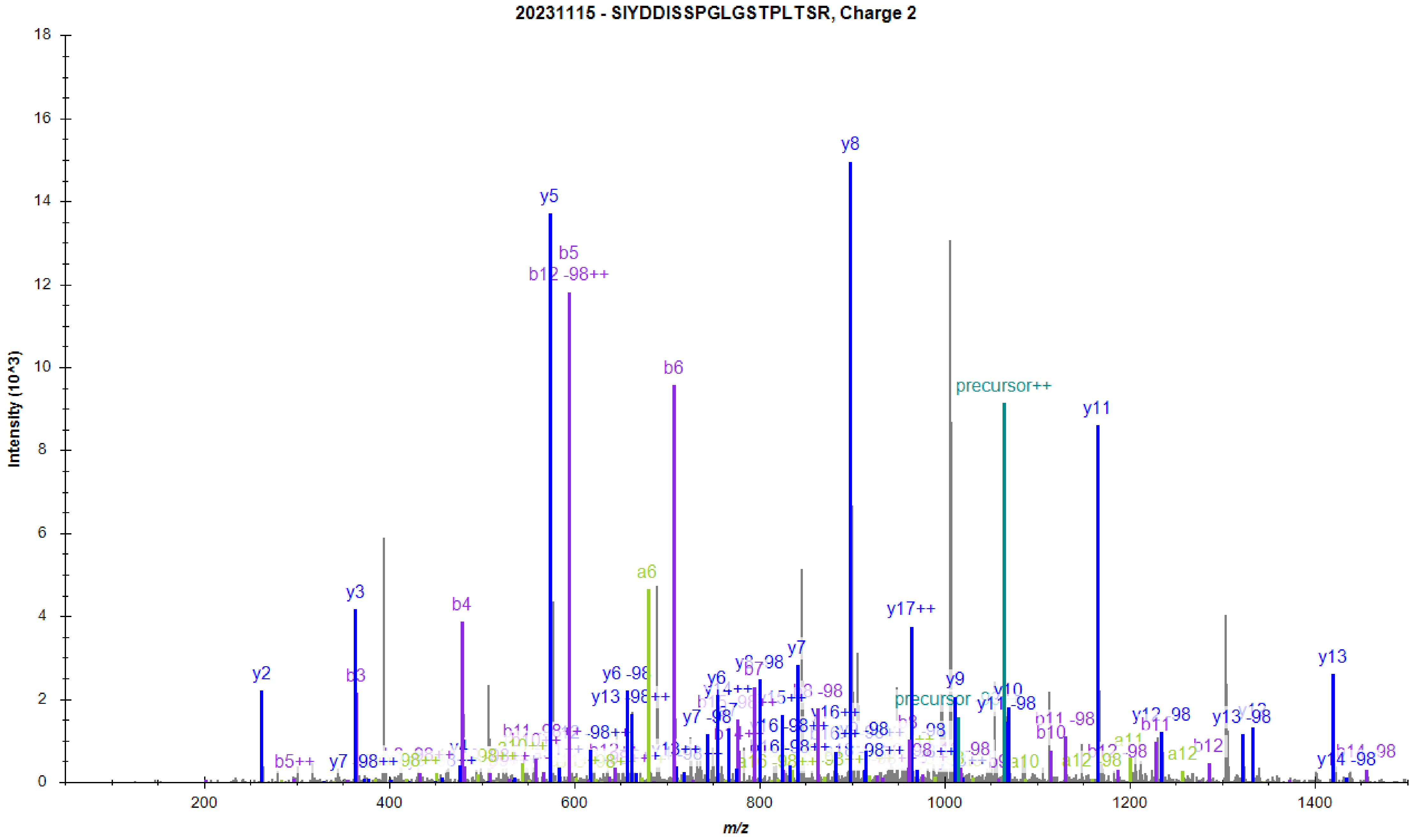

NUP50_T246

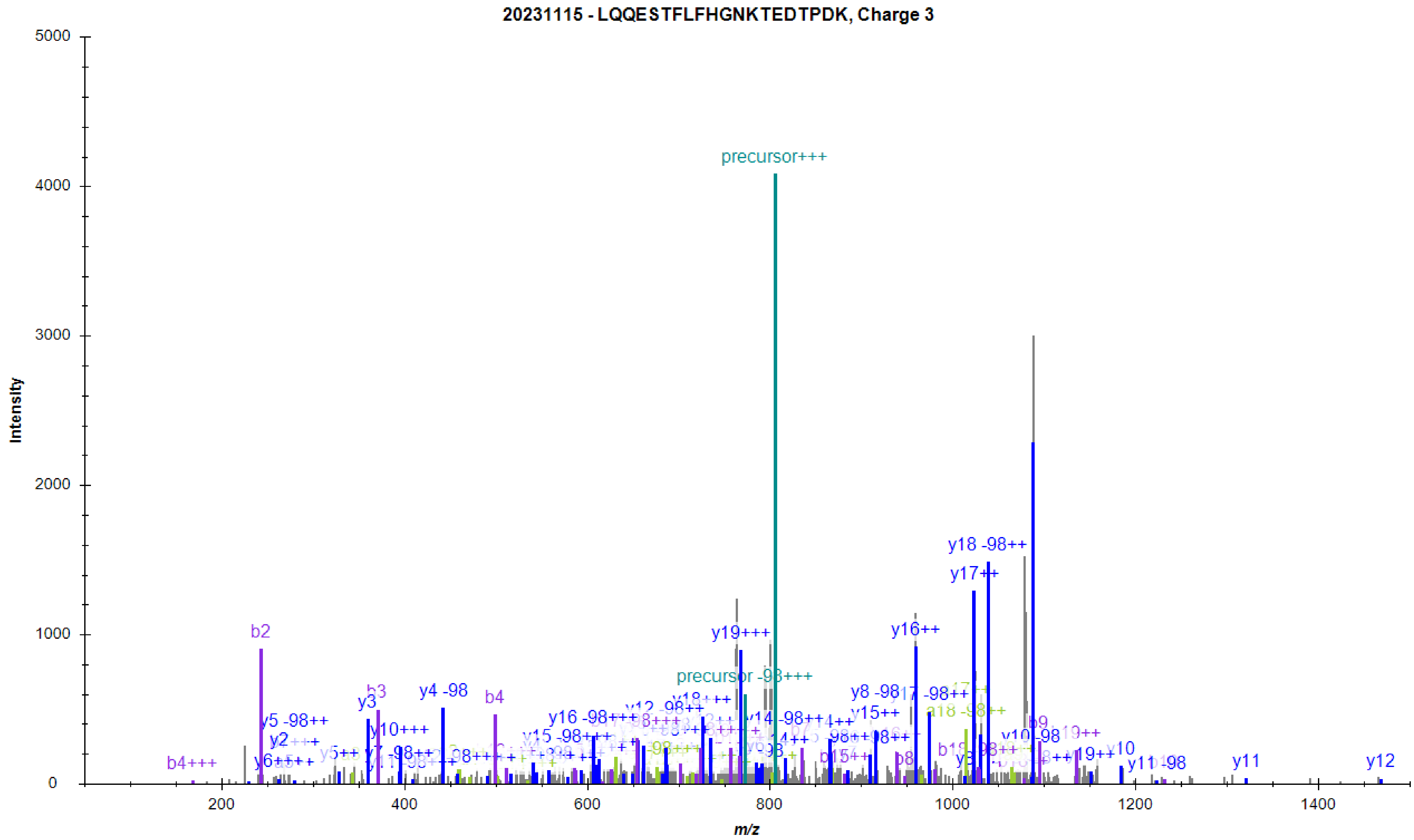
20231115 - LQQESTFLFHGNKTEDTPDK, Charge 3
Intensity
m/z
precursor+++
precursor -98+++
y18 -98++
y17++
y16++
y19+++
b2
b3
y3
y4 -98
b4
b4+++
y5 -98++
y2
y10+++
y16 -98+++
y15 -98+++
y15++
y8 -98
b9
y10
y11
y12
a18 -98++
b15++
b8

NUP98_S1060_1070

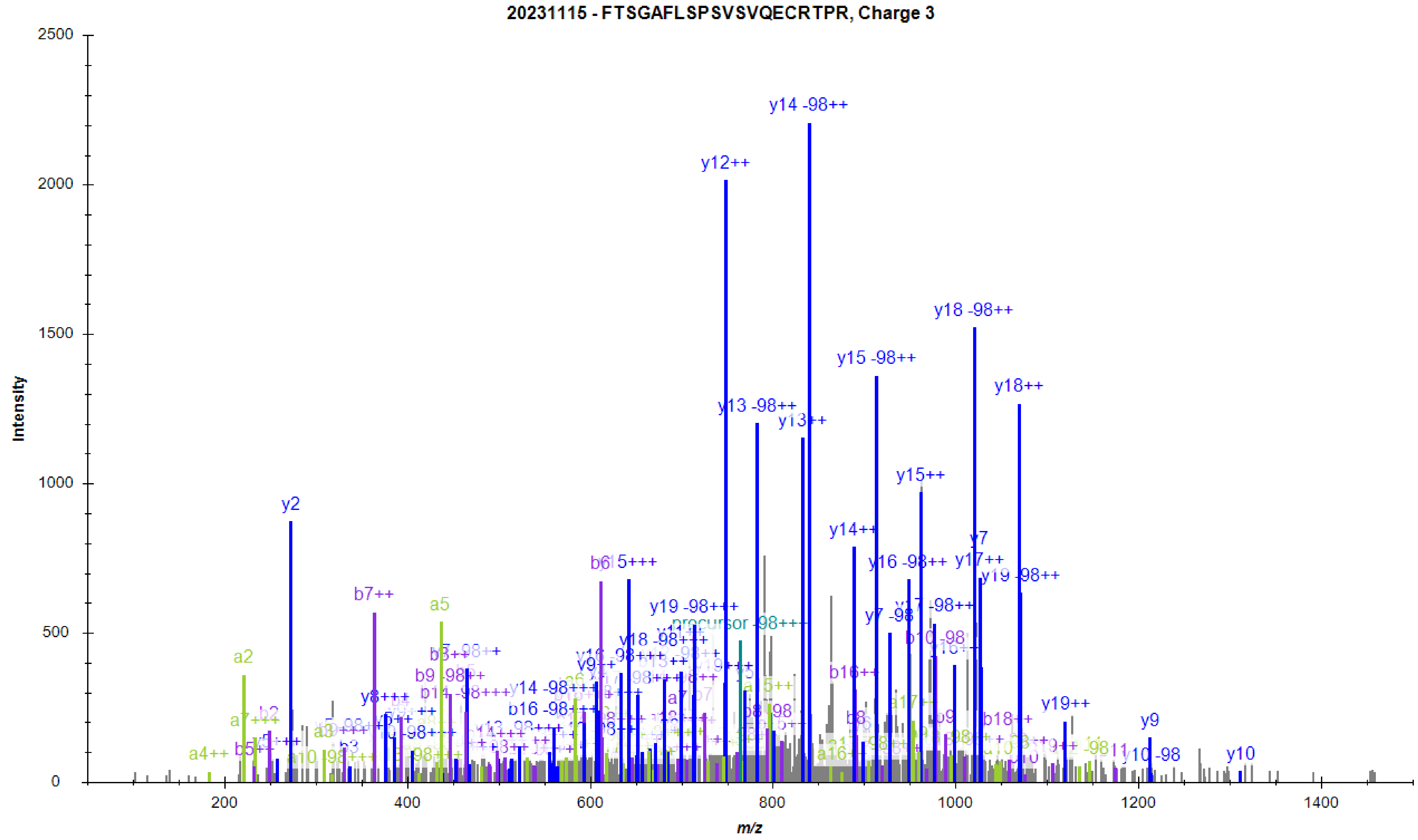

NUP98_T546_T553

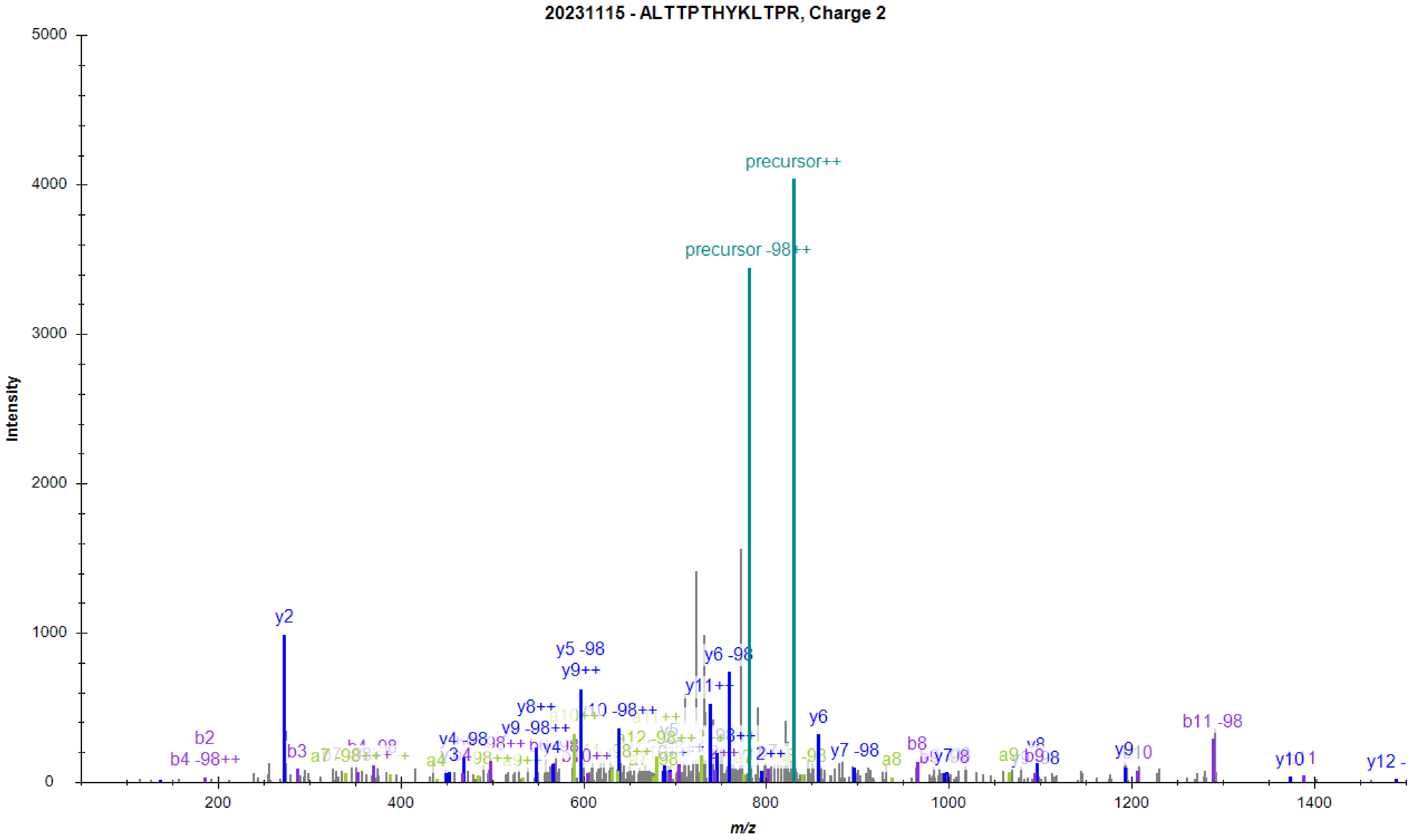

NUP107_S11

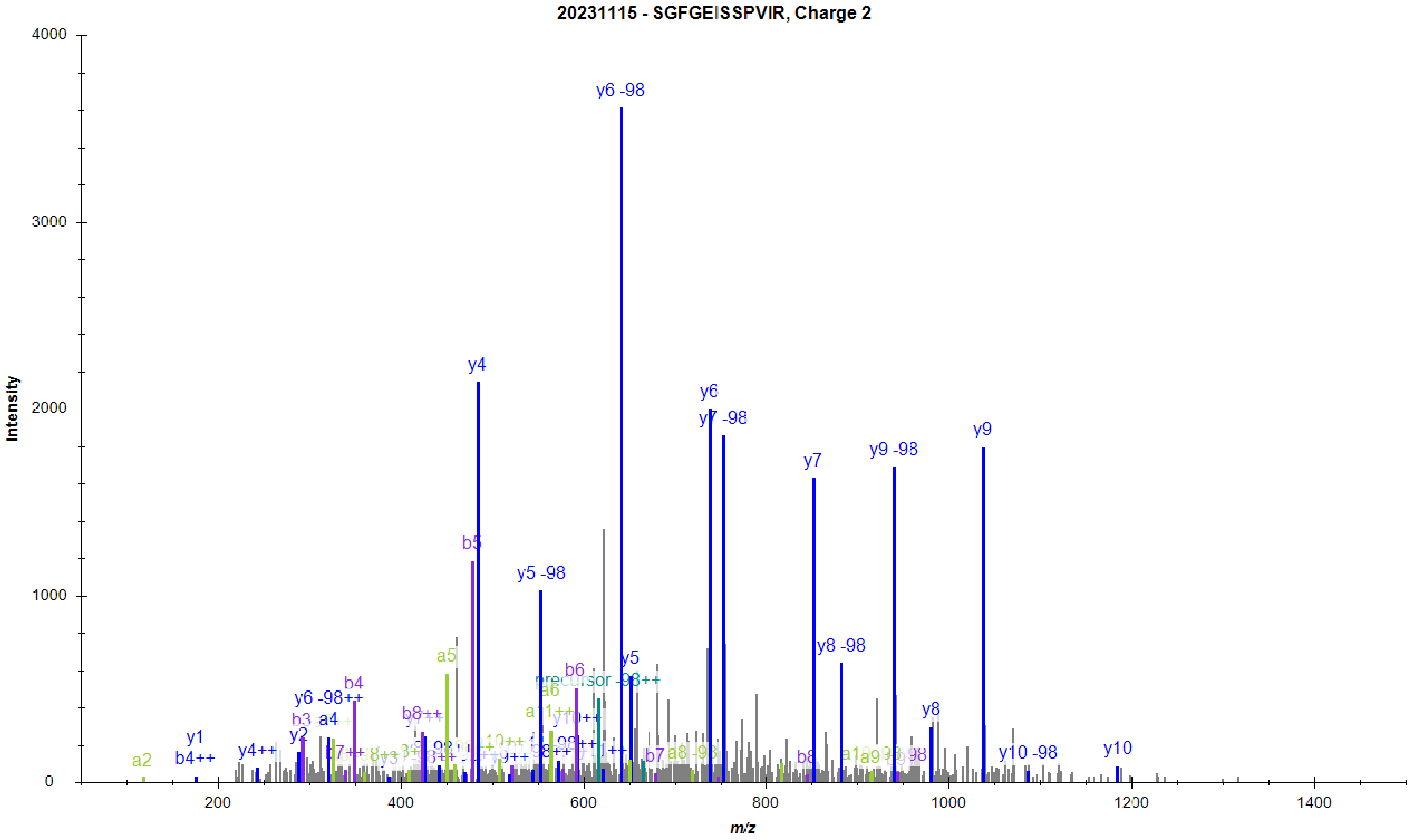
20231115 - SGFGEISSPVIR, Charge 2
Intensity
m/z
4000
3000
2000
1000
0
200
400
600
800
1000
1200
1400
y6 -98
y4
y6
y7 -98
y9
y9 -98
y7
b5
y5 -98
y8 -98
a5
b6
y5
y8
b4
y6 -98++
a4
b3
y2
y1
b4++
y4++
a2
b7
y10 -98
y10

NUP107_S58_T64

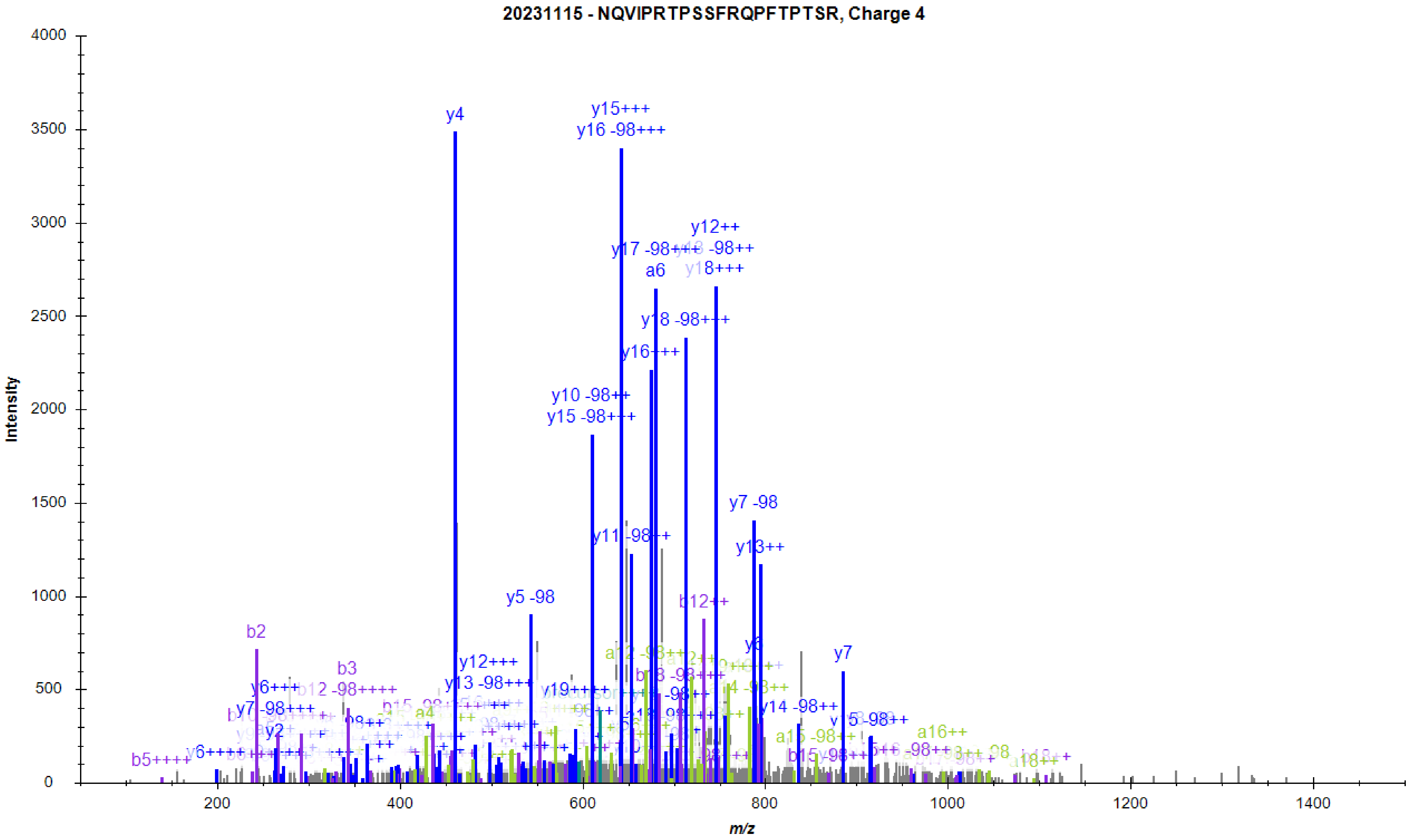

NUP107_S86

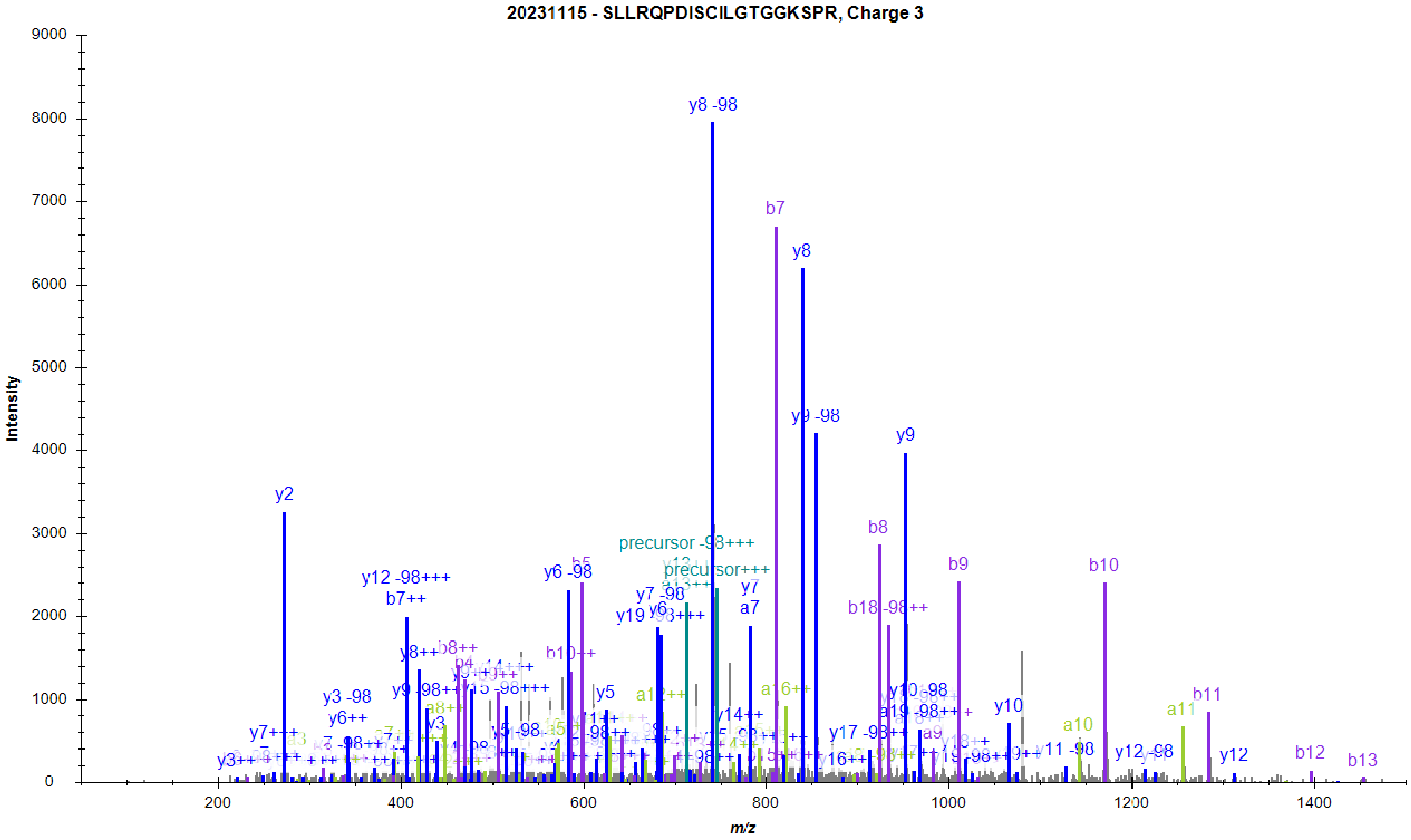
20231115 - SLLRQPDISCILGTGGKSPR, Charge 3
Intensity
m/z
0
1000
2000
3000
4000
5000
6000
7000
8000
9000
200
400
600
800
1000
1200
1400
y8 -98
b7
y8
y9 -98
y9
y2
b8
b9
b10
precursor -98+++
precursor+++
y12 -98+++
b7++
y6 -98
y7 -98
y7
a7
y19
b18 -98++
y10 -98
y10
a10
a11
b11
y12 -98
y12
b12
b13
y3 -98
y6++
y7+++
y5
y14++
a16++
y17 -98++
y16++
y11 -98

NUP133_S27

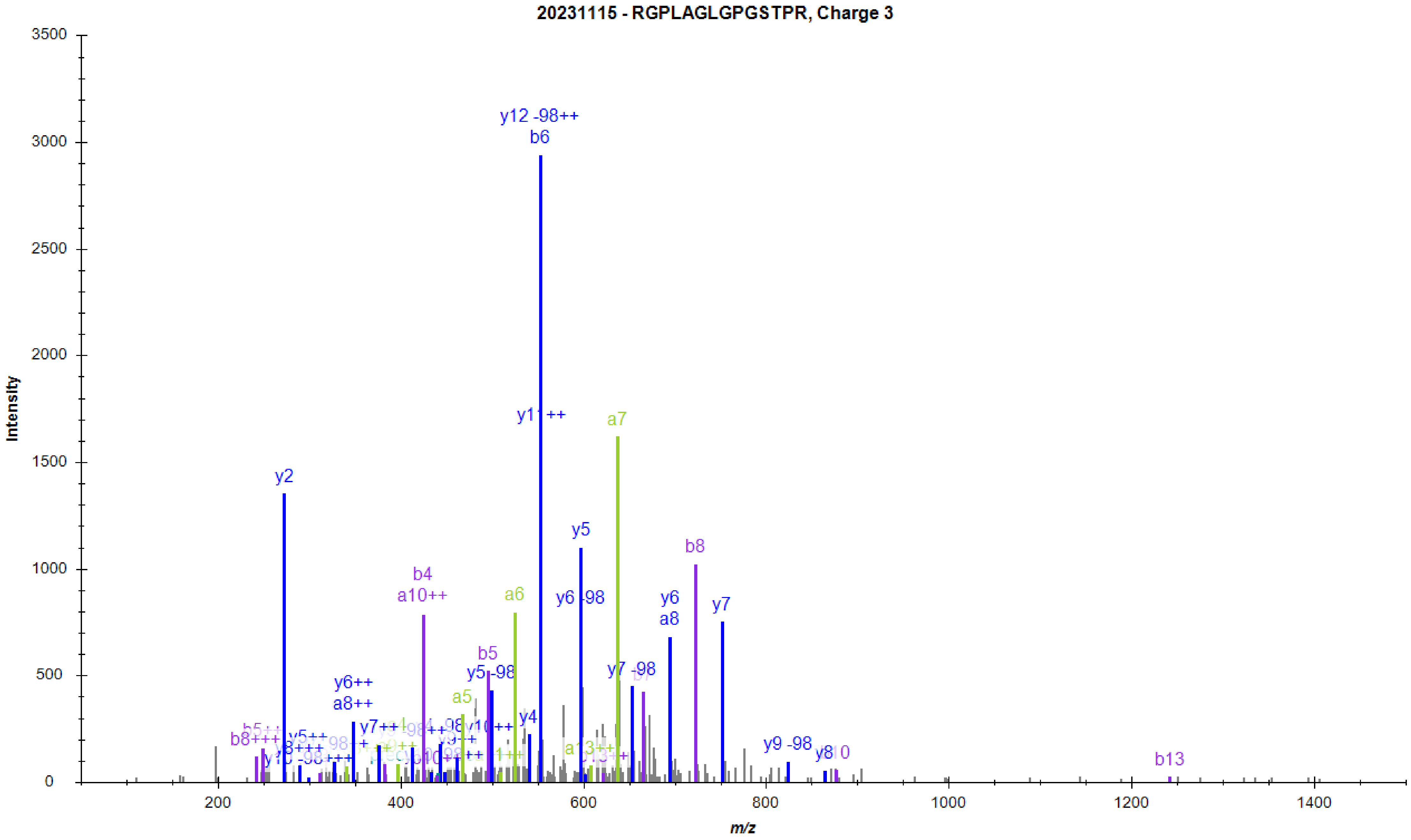
20231115 - RGPLAGLGPGSTPR, Charge 3
Intensity
m/z
y12 -98++
b6
y11++
a7
y2
y5
b8
b4
a10++
a6
y6 -98
y6
a8
y7
b5
y5 -98
y7 -98
y6++
a8++
a5
y4
y9 -98
b13

NUP133_S50_S45

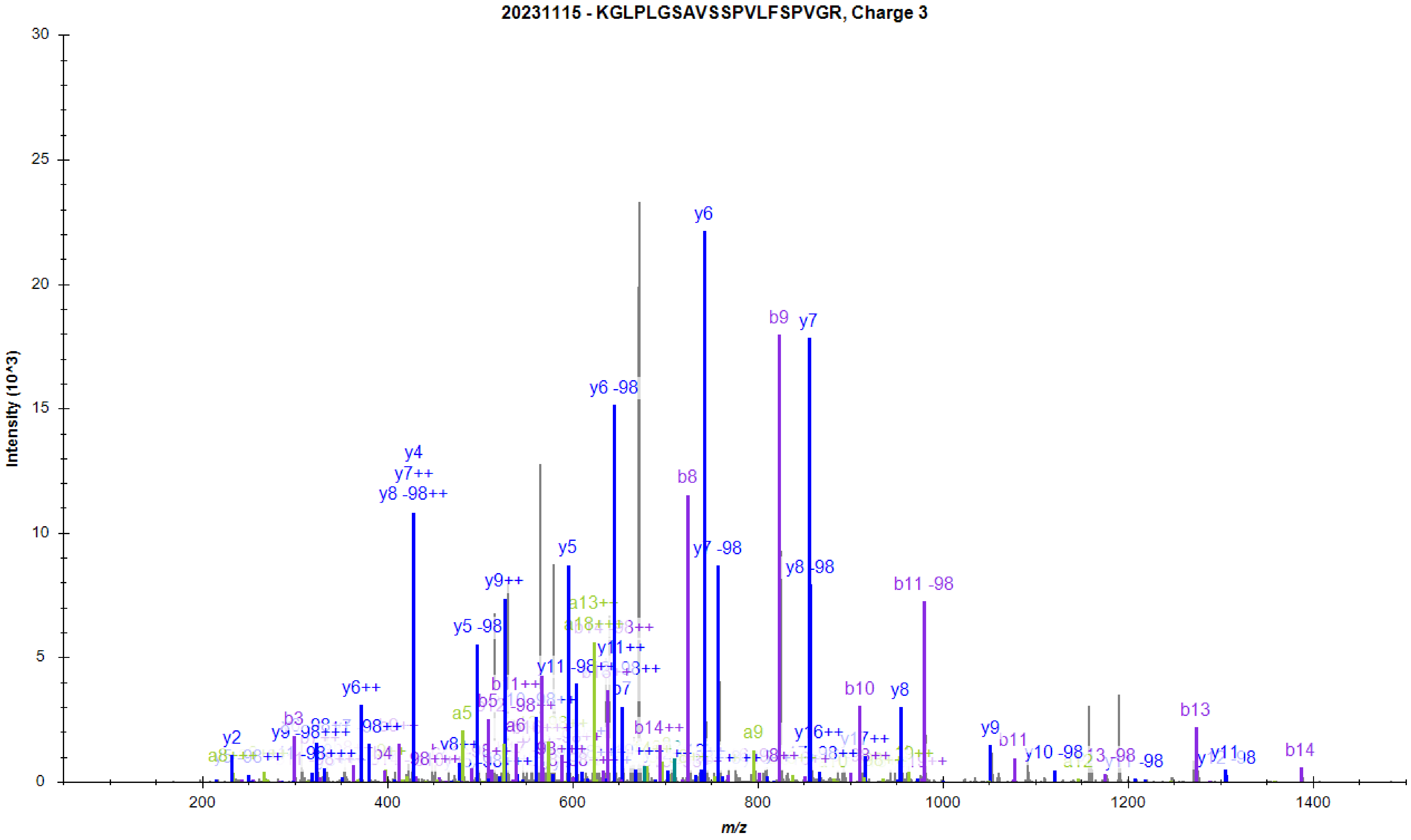

NUP133_S56_S60_T63

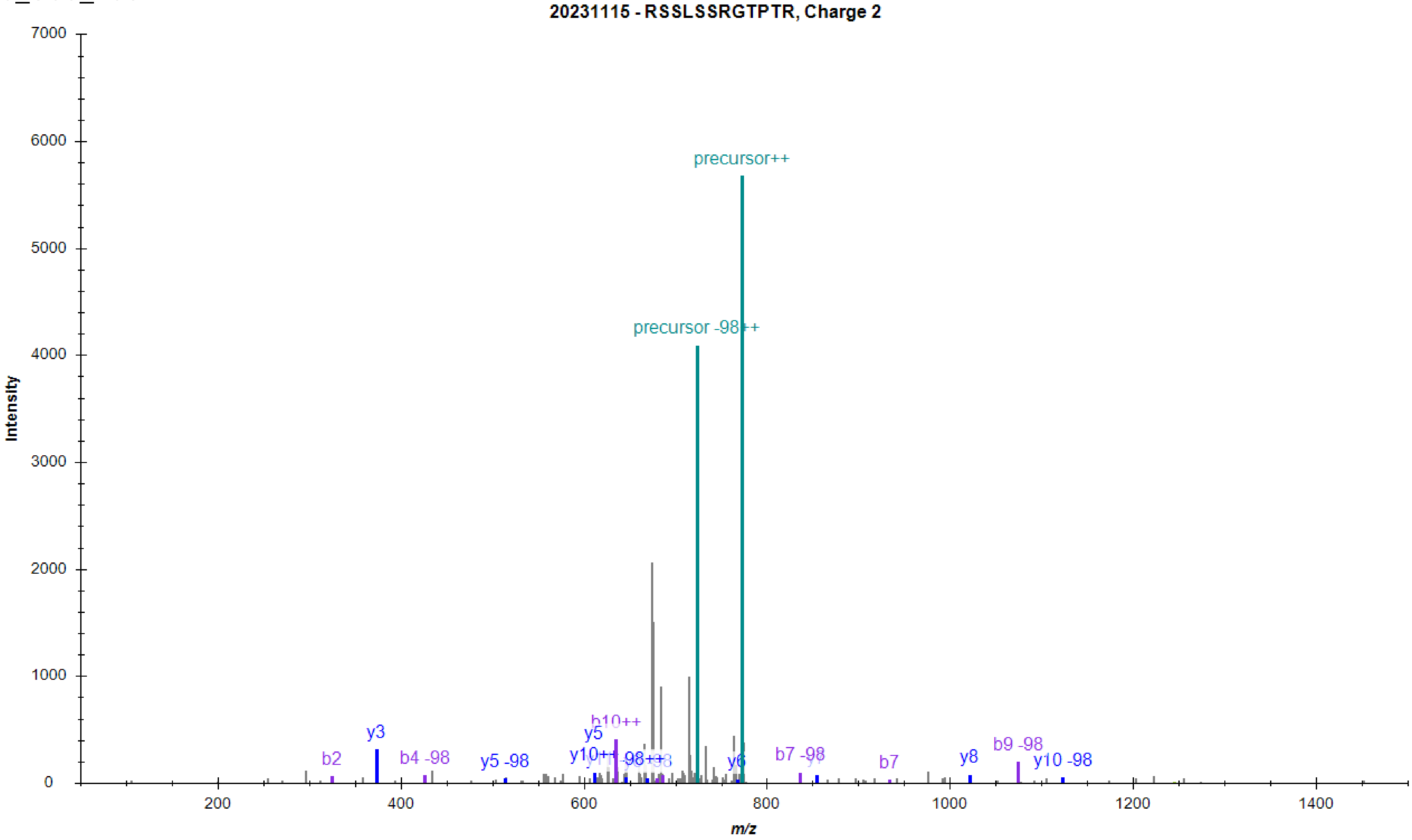

NUP133_T10

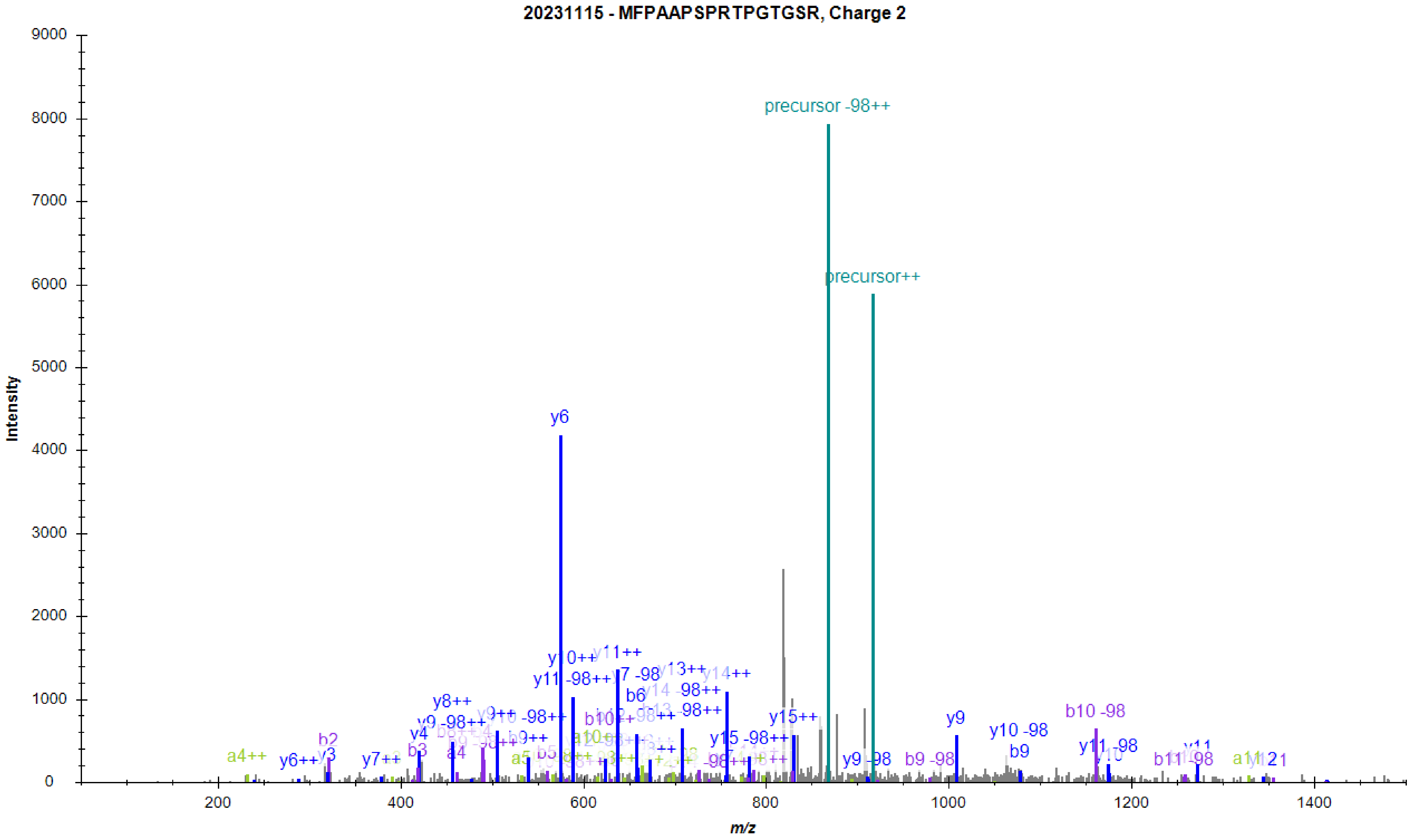
20231115 - MFPAAPSPRTPGTGSR, Charge 2
Intensity
m/z
precursor -98++
precursor++
y6
y10++
y11++
y11 -98++
y7 -98
y13++
y14++
b6
y14 -98++
y8++
y9 -98++
y9++
b10++
y13 -98++
y15++
y9
y10 -98
b10 -98
y4
b3
a4
b2
y3
a4++
y6++
y7++
y15 -98++
b9
y11 -98
y9 -98
b9 -98
b11 -98
a11
y12
y11

NUP153_S257

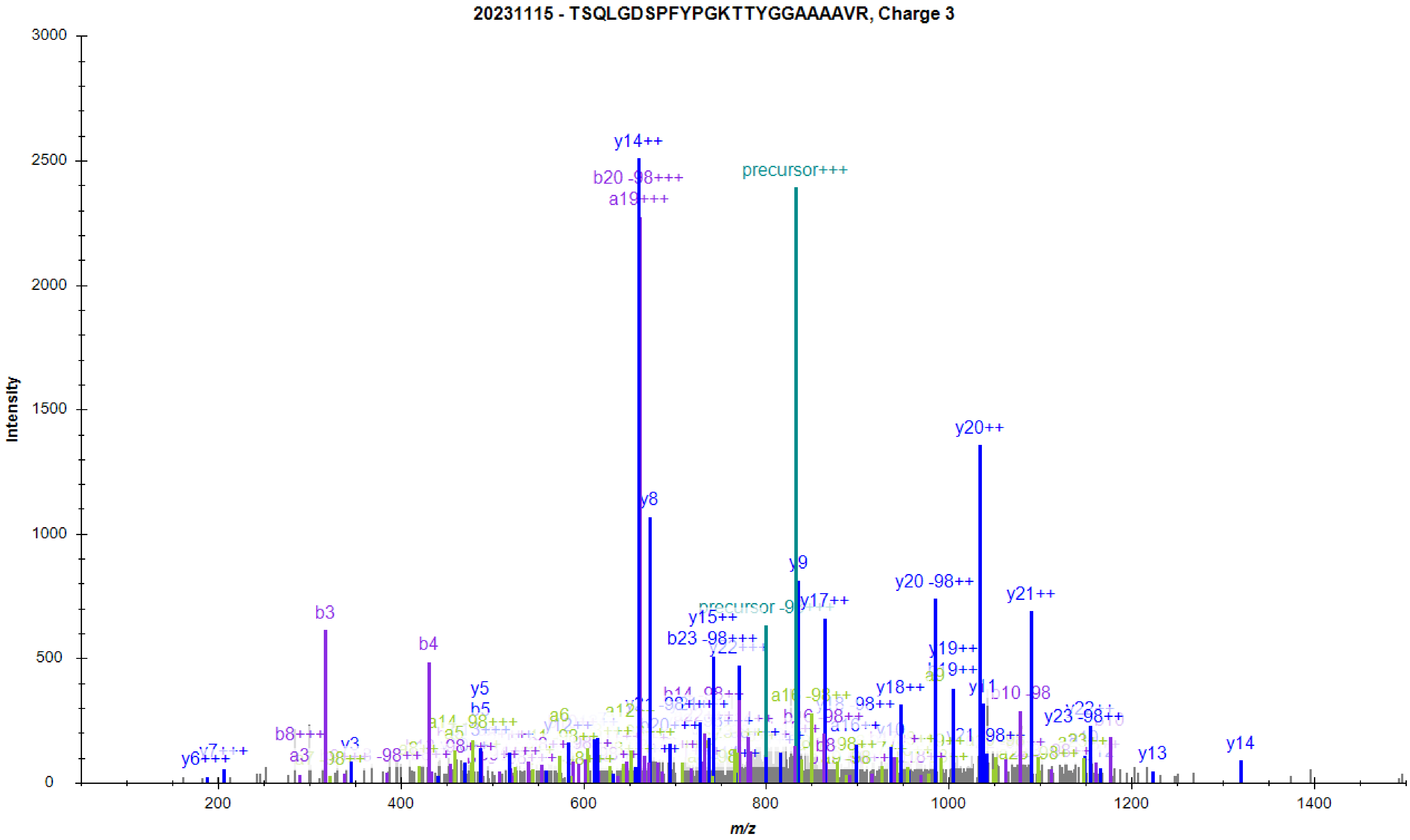

20231115 - TSQLGDSPFYPGKTTYGGAAAAVR, Charge 3
Intensity
m/z
3000
2500
2000
1500
1000
500
0
200
400
600
800
1000
1200
1400
y14++
b20 -98+++
a19+++
precursor+++
y20++
y8
y9
y17++
y20 -98++
y21++
b3
b4
y15++
b23 -98+++
y19++
y18++
y5
b5
b10 -98
y23 -98++
y13
y14
b8+++
a3
y6+++
a6

NUP153_S320

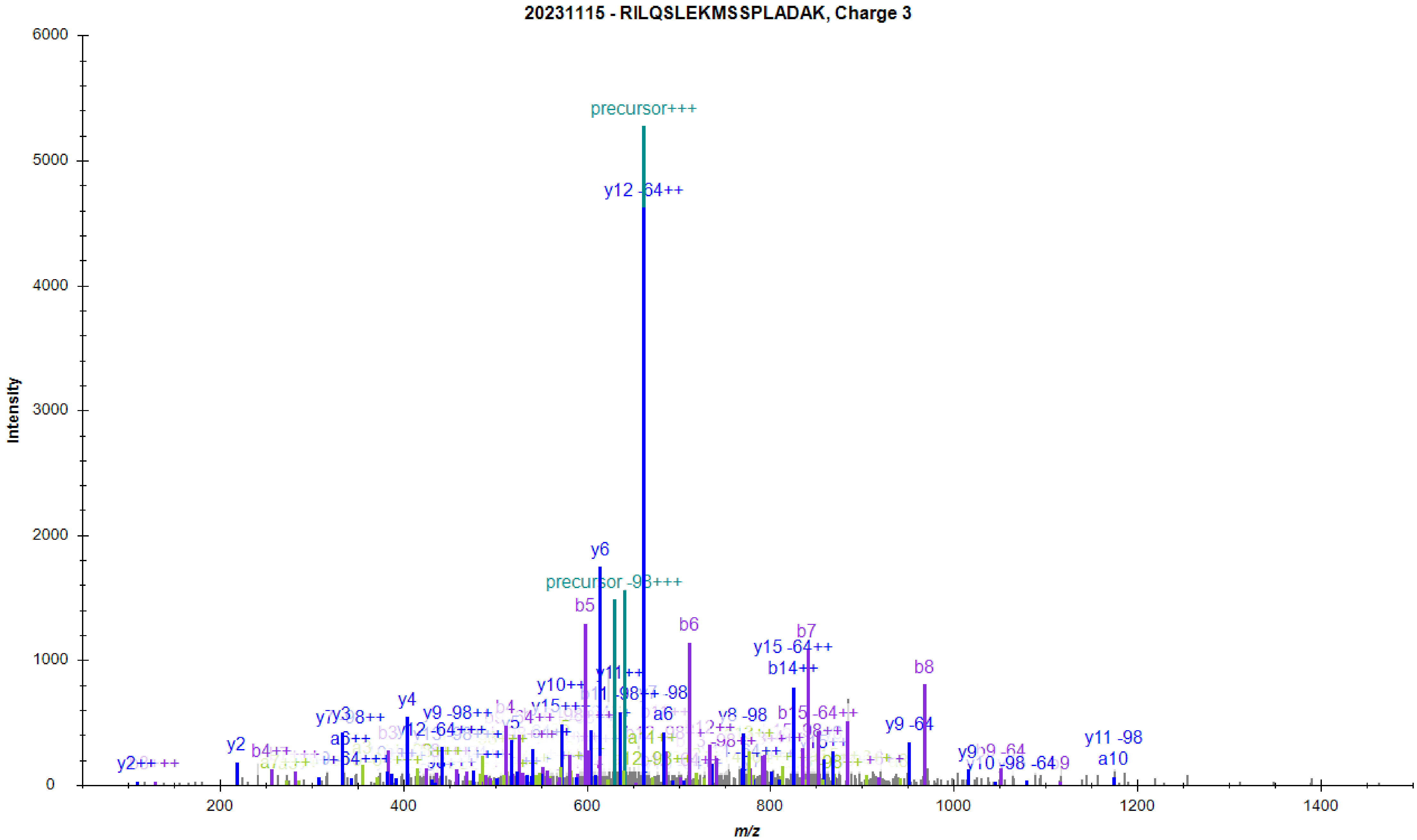
20231115 - RILQSLEKMSSPLADAK, Charge 3
Intensity
m/z
0
1000
2000
3000
4000
5000
6000
200
400
600
800
1000
1200
1400
precursor+++
y12 -64++
y6
precursor -98+++
b5
b6
b7
y15 -64++
b14++
b8
y11++
y10++
y4
y2
y2++
b4++
a6
y8 -98
b15 -64++
y9 -64
y11 -98
a10
y9 -98++

NUP153_S334_338

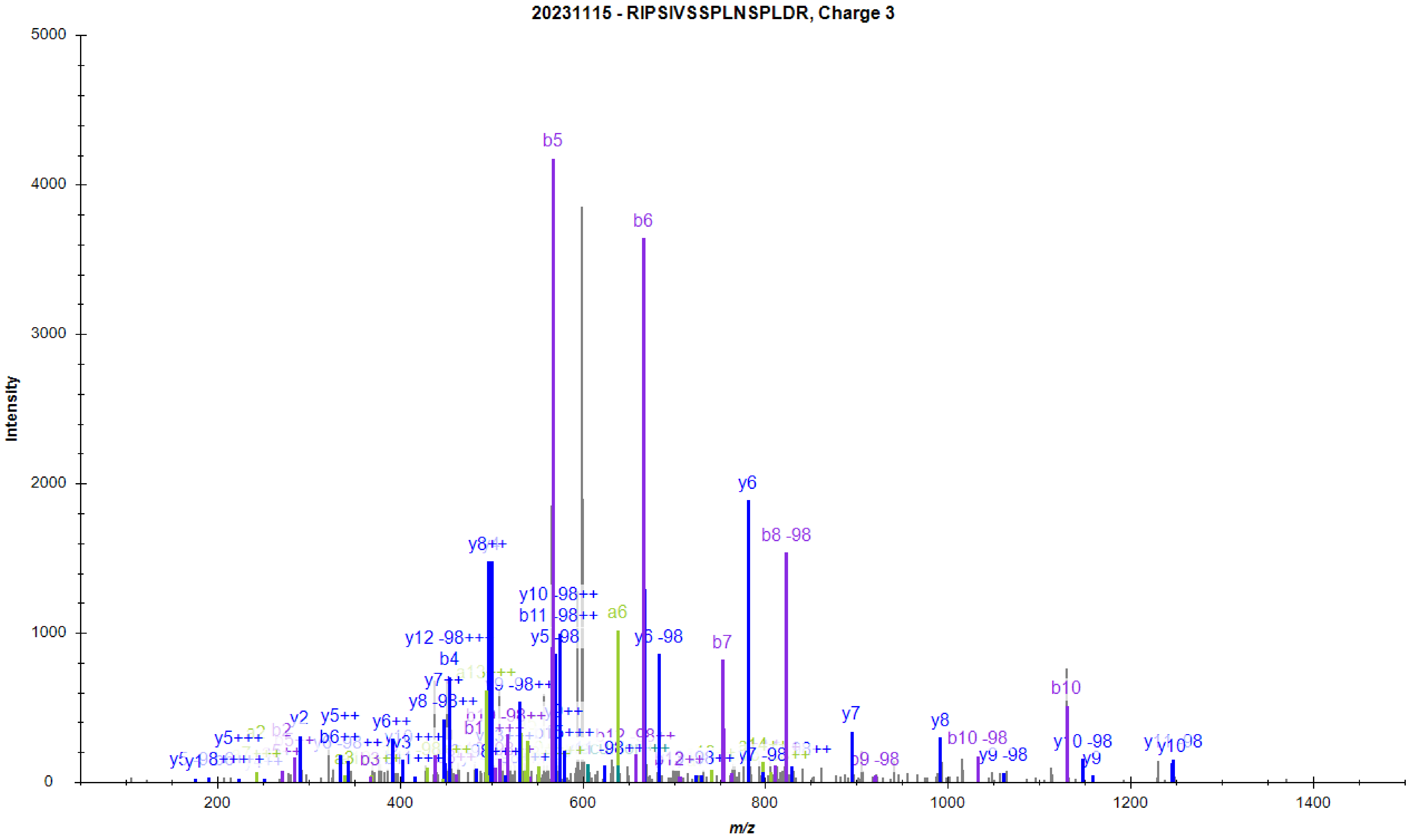

NUP153_S369

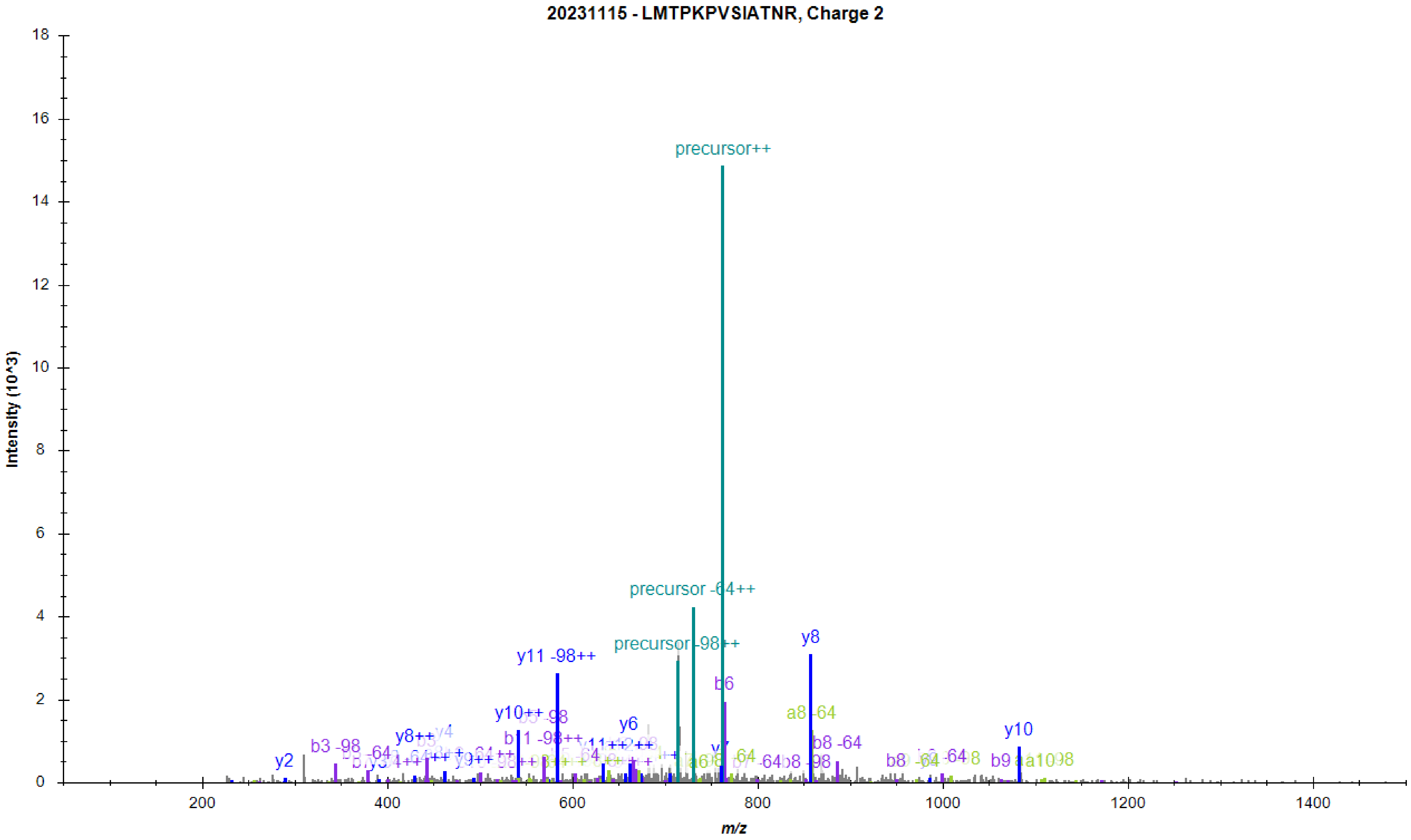
20231115 - LMTPKPVSIATNR, Charge 2
Intensity (10^3)
m/z
precursor++
precursor -64++
precursor -98++
y11 -98++
y10++
y8++
y8
y6
y2
y10
b6
b3 -98
a8 -64
b8 -64
b8 -98
b9

NUP153_S516

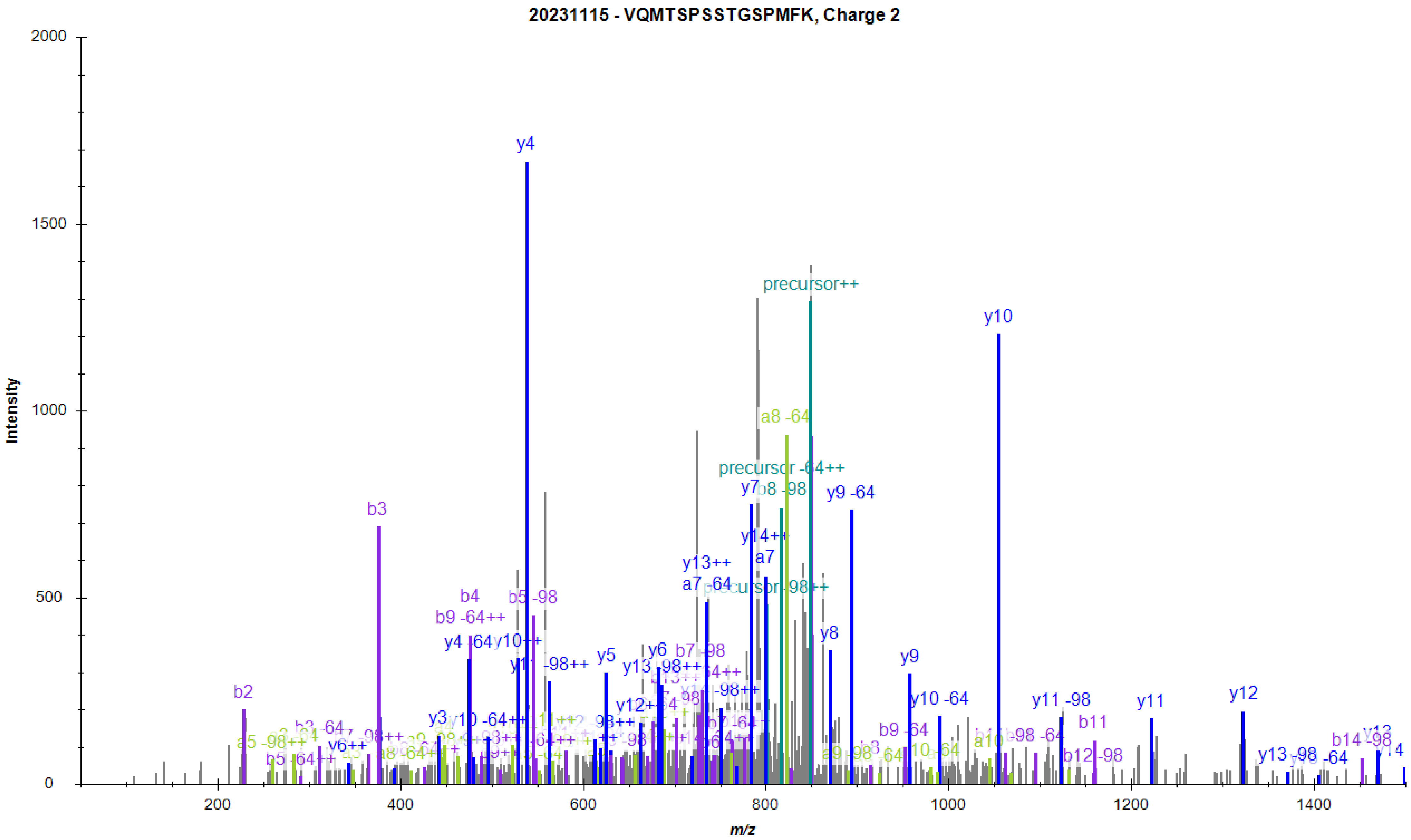

20231115 - VQMTSPSSTGSPMFK, Charge 2
Intensity
m/z
y4
y10
precursor++
a8 -64
precursor -64++
y7
b8 -98
y9 -64
b3
y14++
a7
y13++
a7 -64
b4
b5 -98
b9 -64++
y4 -64
y10++
y8
y5
y6
y9
y10 -64
y11 -98
y11
y12
b11
b2
y3
b9 -64
b12 -98
y13 -98
b14 -98

NUP153_S687

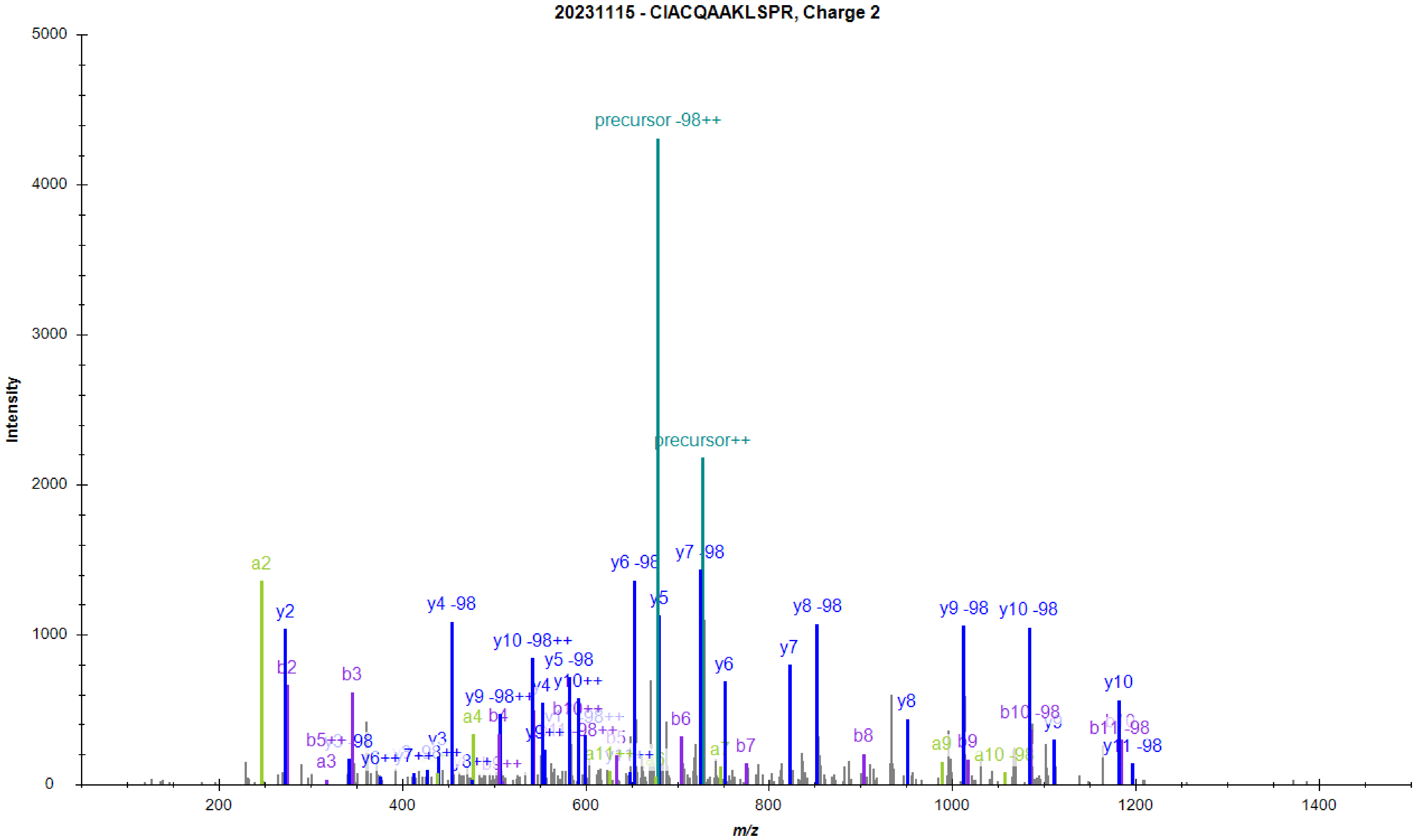
20231115 - CIACQAAKLSPR, Charge 2
Intensity
m/z
precursor -98++
precursor++
a2
y2
b2
b3
y4 -98
y10 -98++
y5 -98
y10++
y9 -98++
y6 -98
y5
y7 -98
y6
y7
y8 -98
y8
y9 -98
y10 -98
y10
b10 -98
b11 -98
y11 -98
b6
b7
b8
a9
b9
a10 -98
a4
b4
a3
y3
0
1000
2000
3000
4000
5000
200
400
600
800
1000
1200
1400

NUP153_T699_2

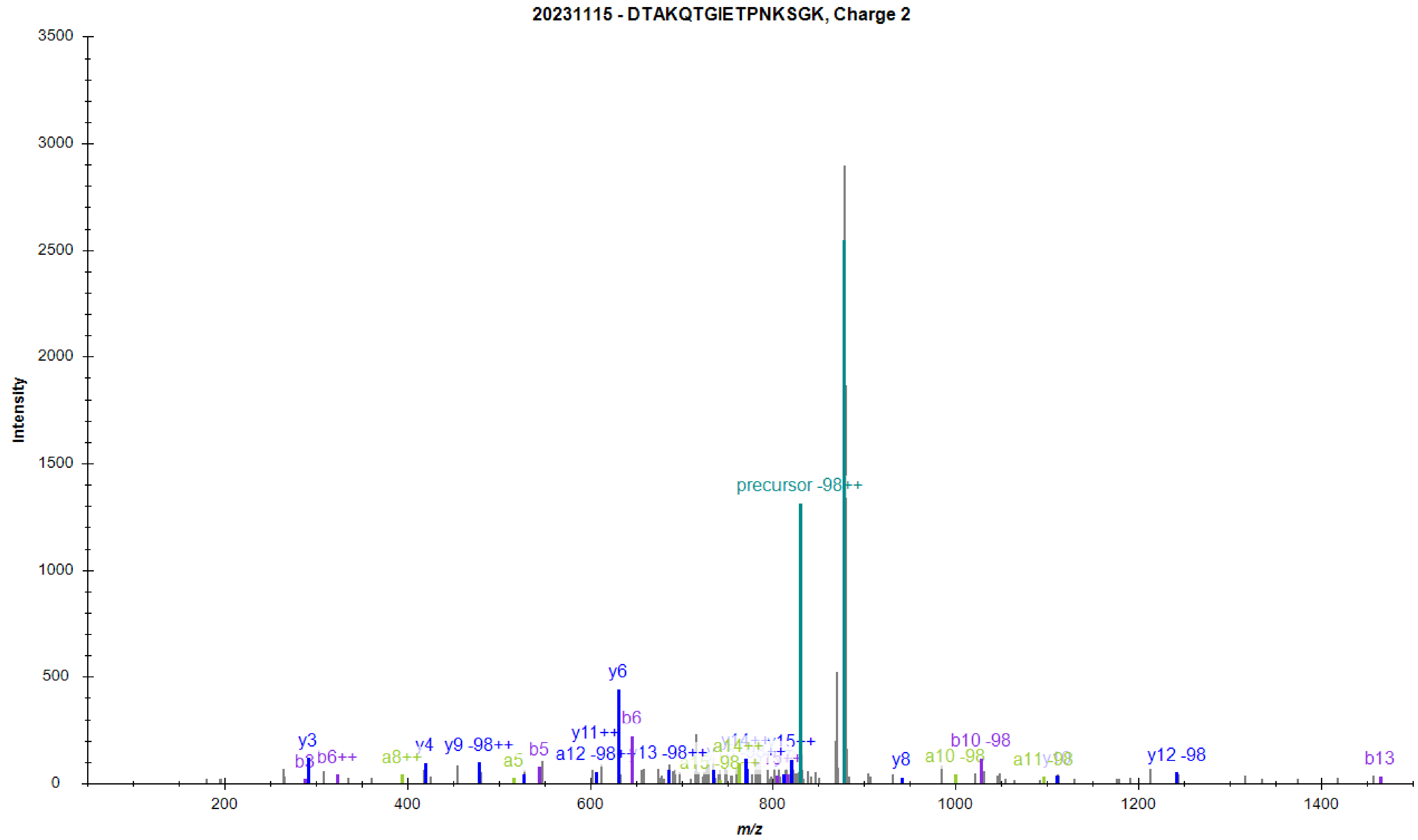

NUP153_T699_3

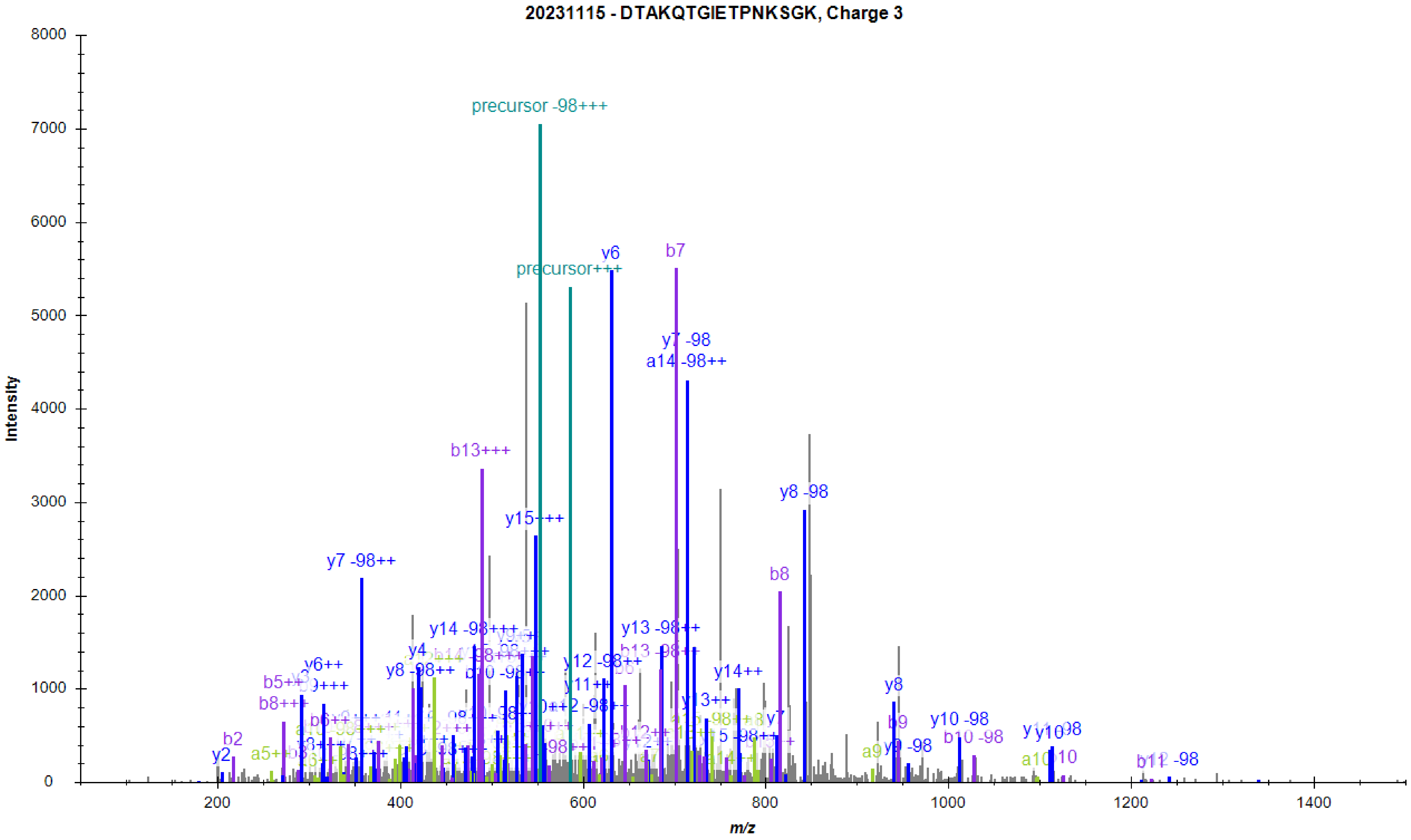

NUP153_T1463

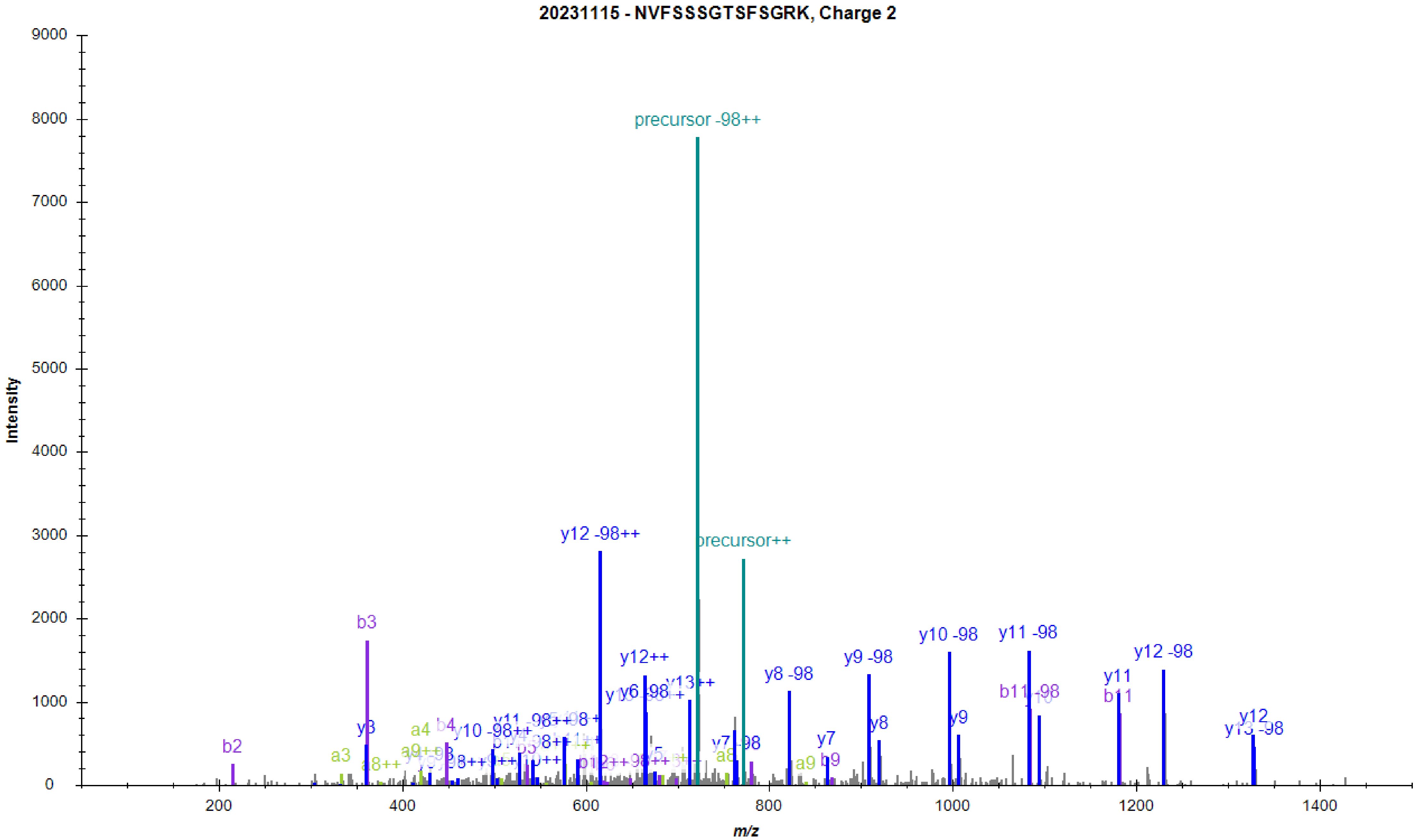

NUP155_S992

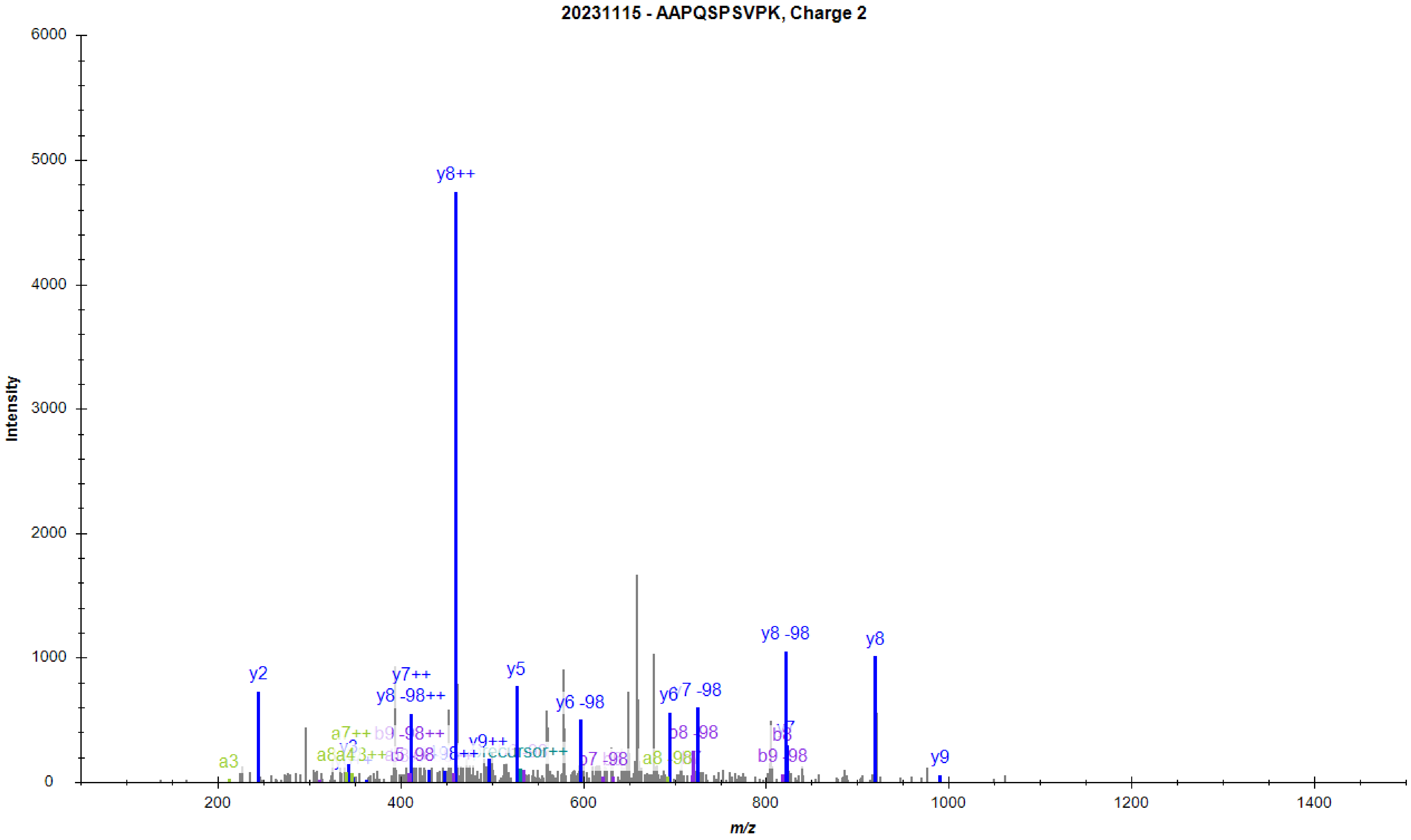
20231115 - AAPQSPSVPK, Charge 2
Intensity
m/z
6000
5000
4000
3000
2000
1000
0
200
400
600
800
1000
1200
1400
y8++
y2
y7++
y8 -98++
y5
y6 -98
y6
y7 -98
y8 -98
y8
y9
y9++
a3
b8 -98
b9 -98
a8

NUP188_S1709_S1717

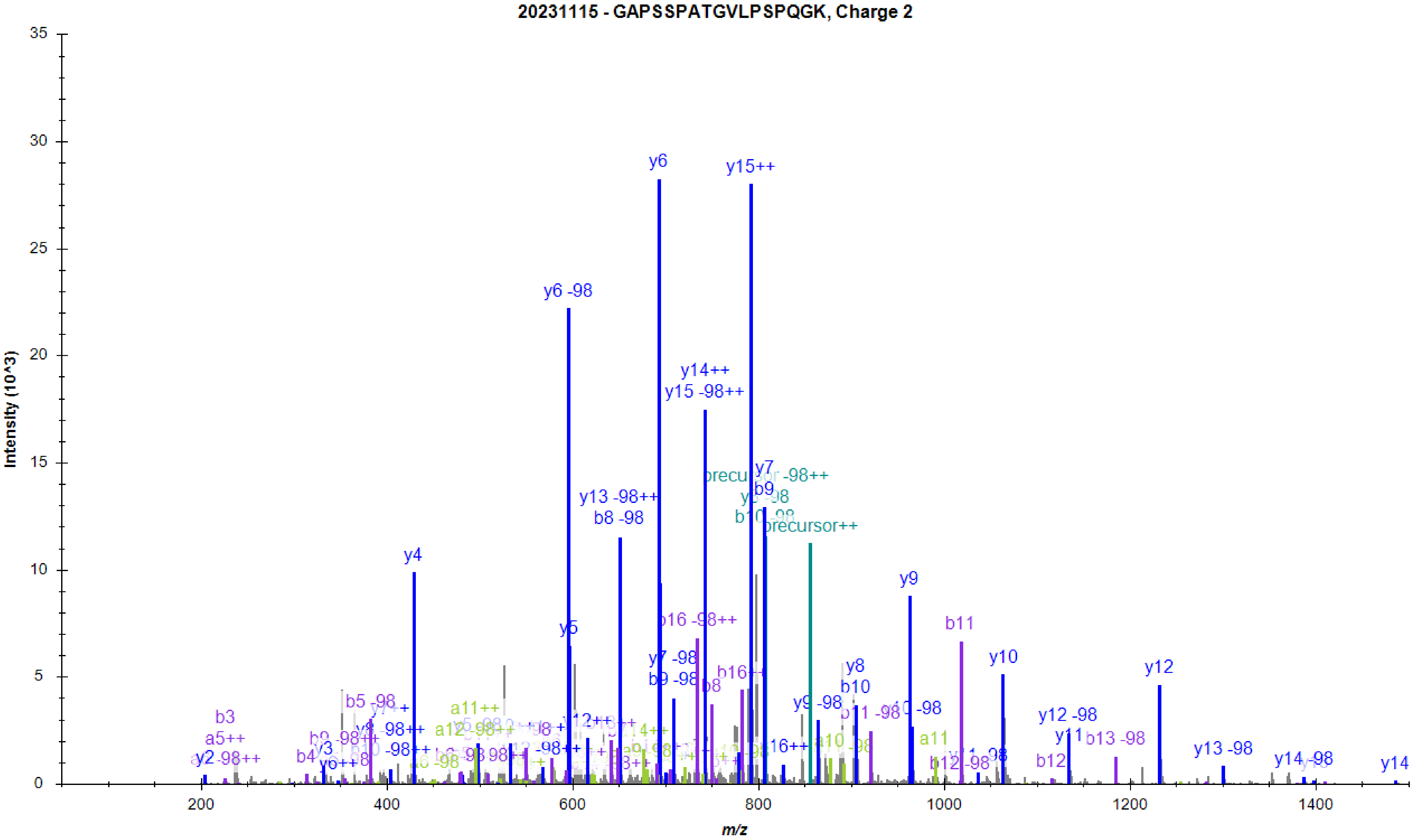

NUP214_T434

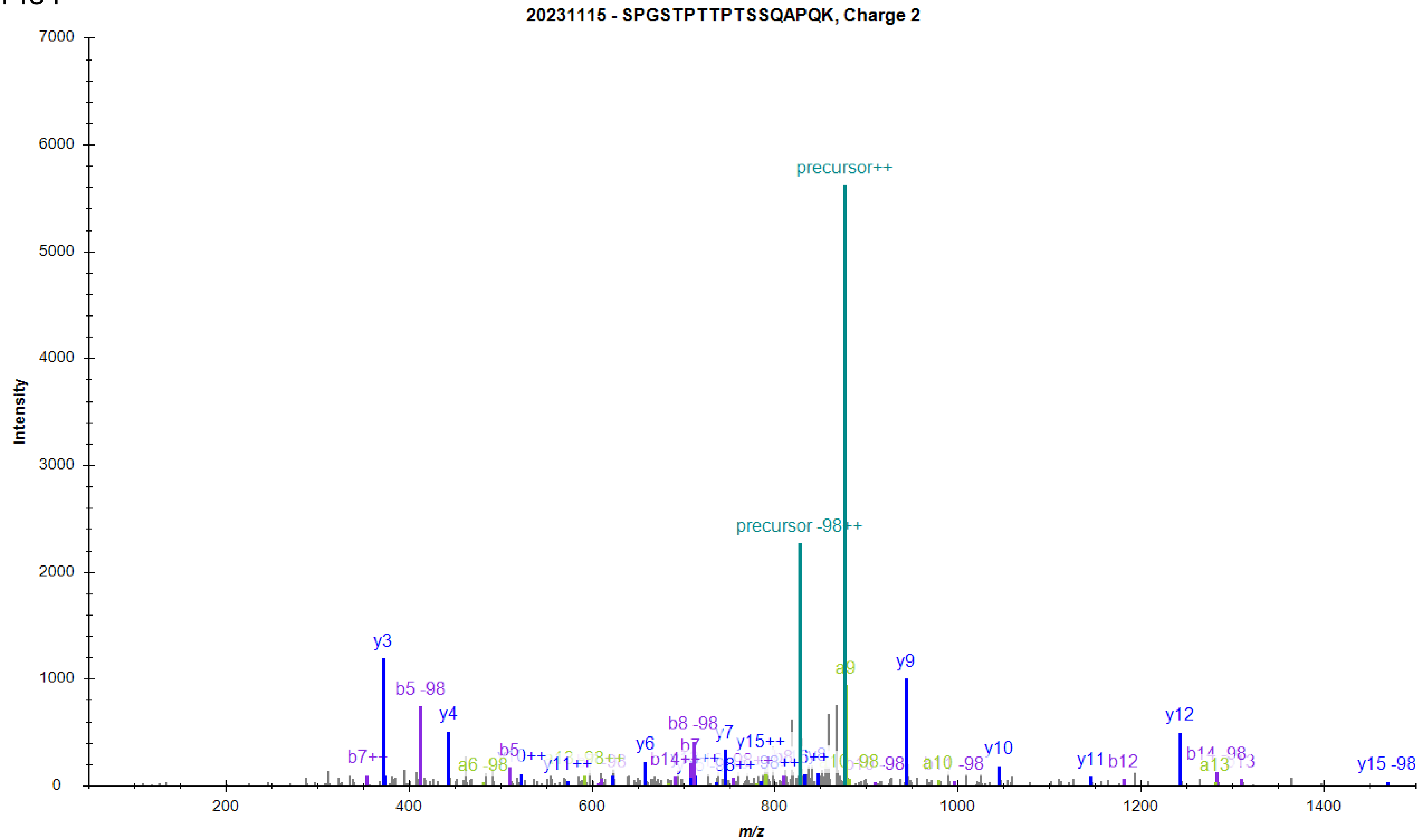
20231115 - SPGSTPTTPTSSQAPQK, Charge 2
Intensity
m/z
precursor++
precursor -98++
y3
b5 -98
y4
y9
a9
y12
y10
y11
b12
y15 -98
y6
y7
b8 -98
y15++
b7++
a6 -98
y11++
a13
a10
0
1000
2000
3000
4000
5000
6000
7000
200
400
600
800
1000
1200
1400

NUP214_T434_T437

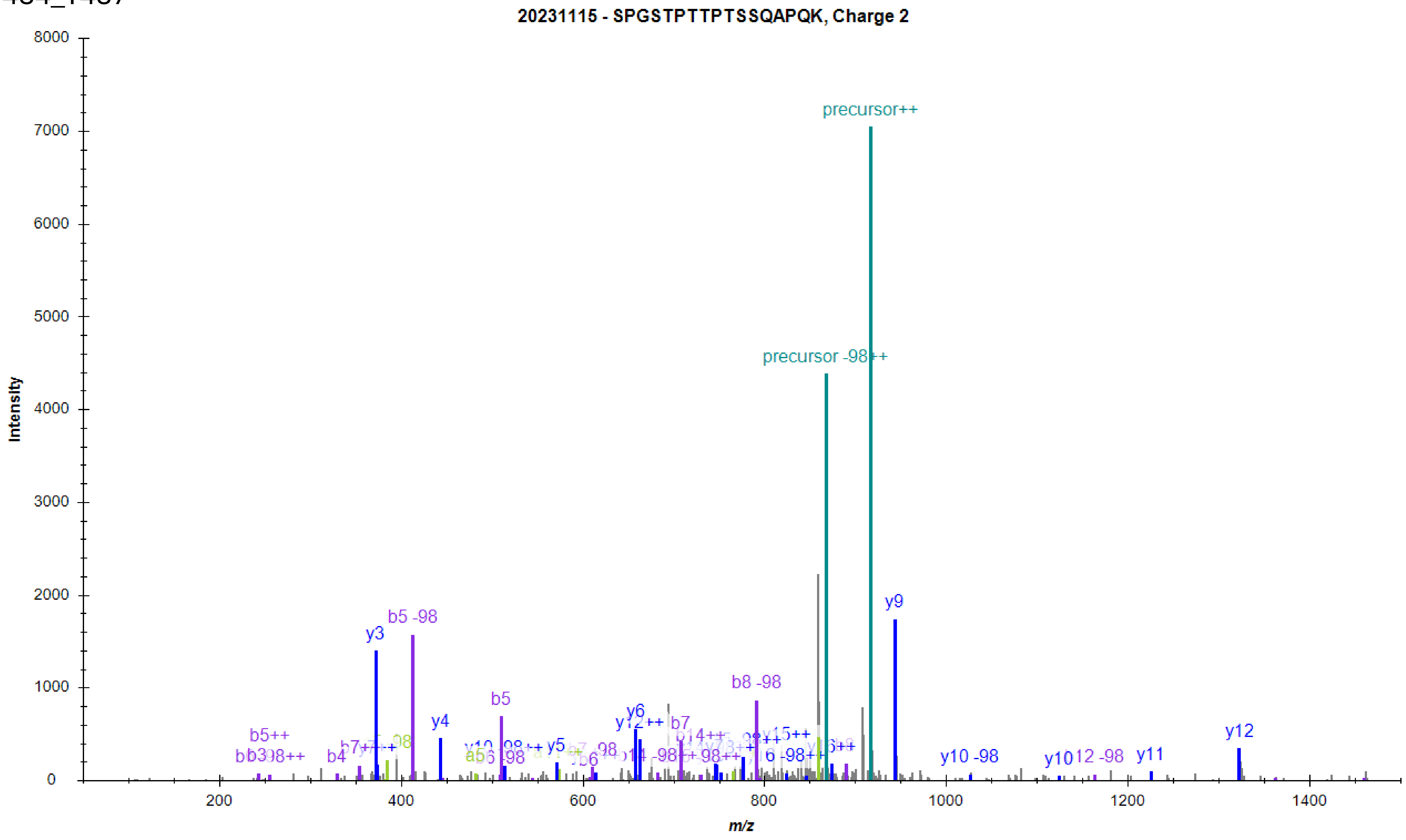

NUP214_T1134

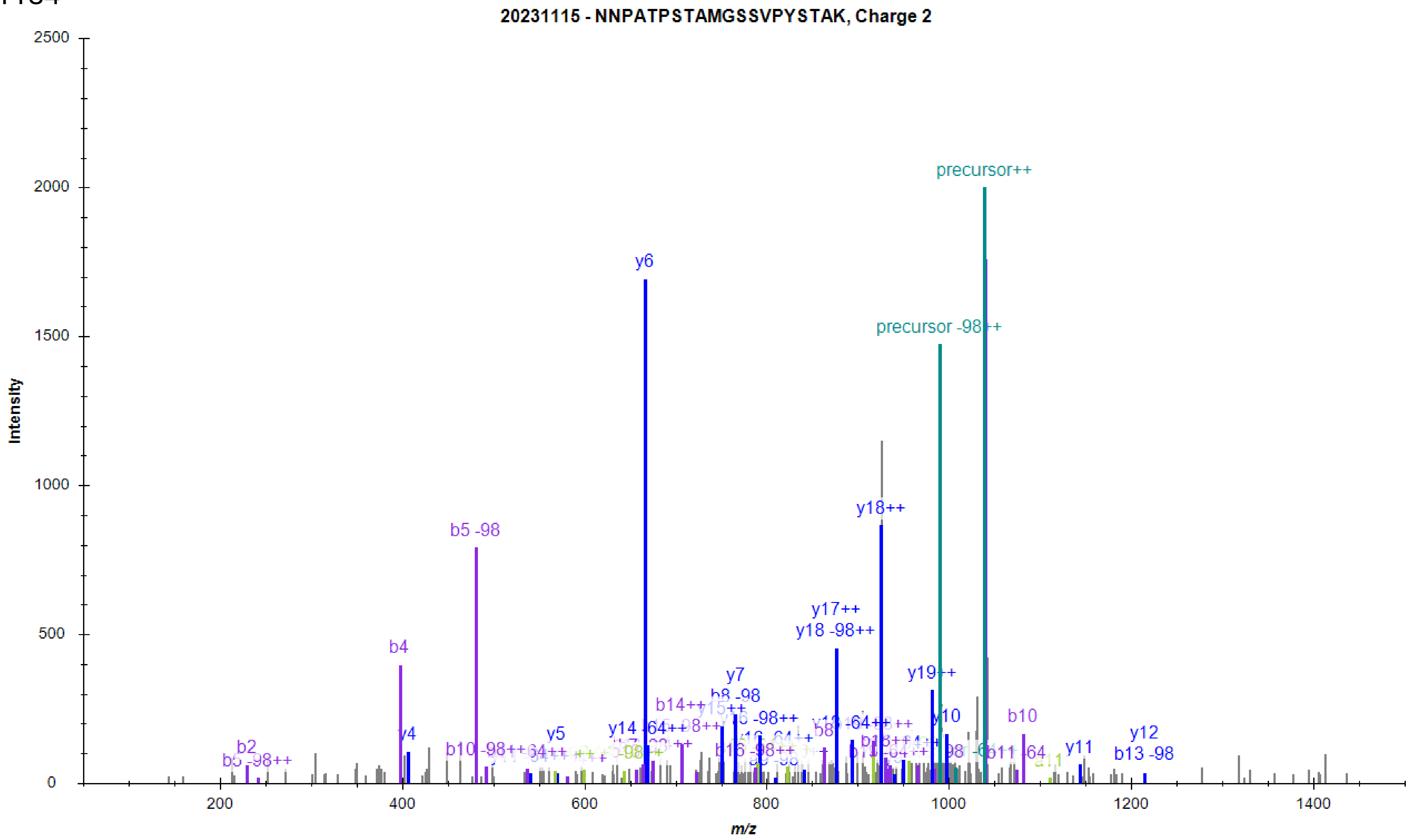

PBK_S59

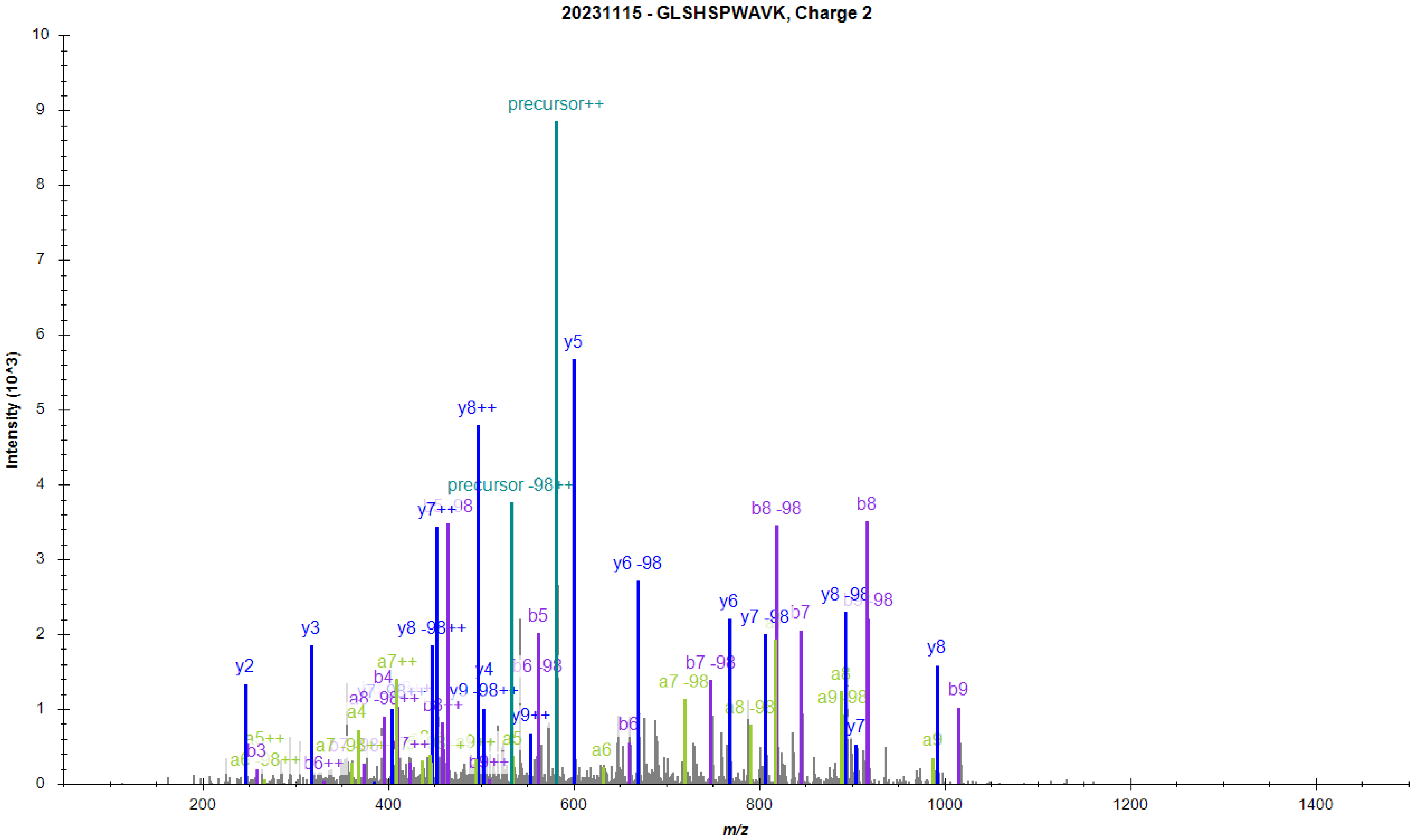
20231115 - GLSHSPWAVK, Charge 2
Intensity (10^3)
m/z
precursor++
y5
y8++
precursor -98++
y7++
b8 -98
b8
y6 -98
y6
y8 -98
b7
y7 -98
b5
y8 -98++
y3
y2
y8
b9
a7++
b4
y4
y9 -98++
b7 -98
a7 -98
a4
y9++
a8 -98
y7
a6
a9
b3
b6

PBK_T24-S32

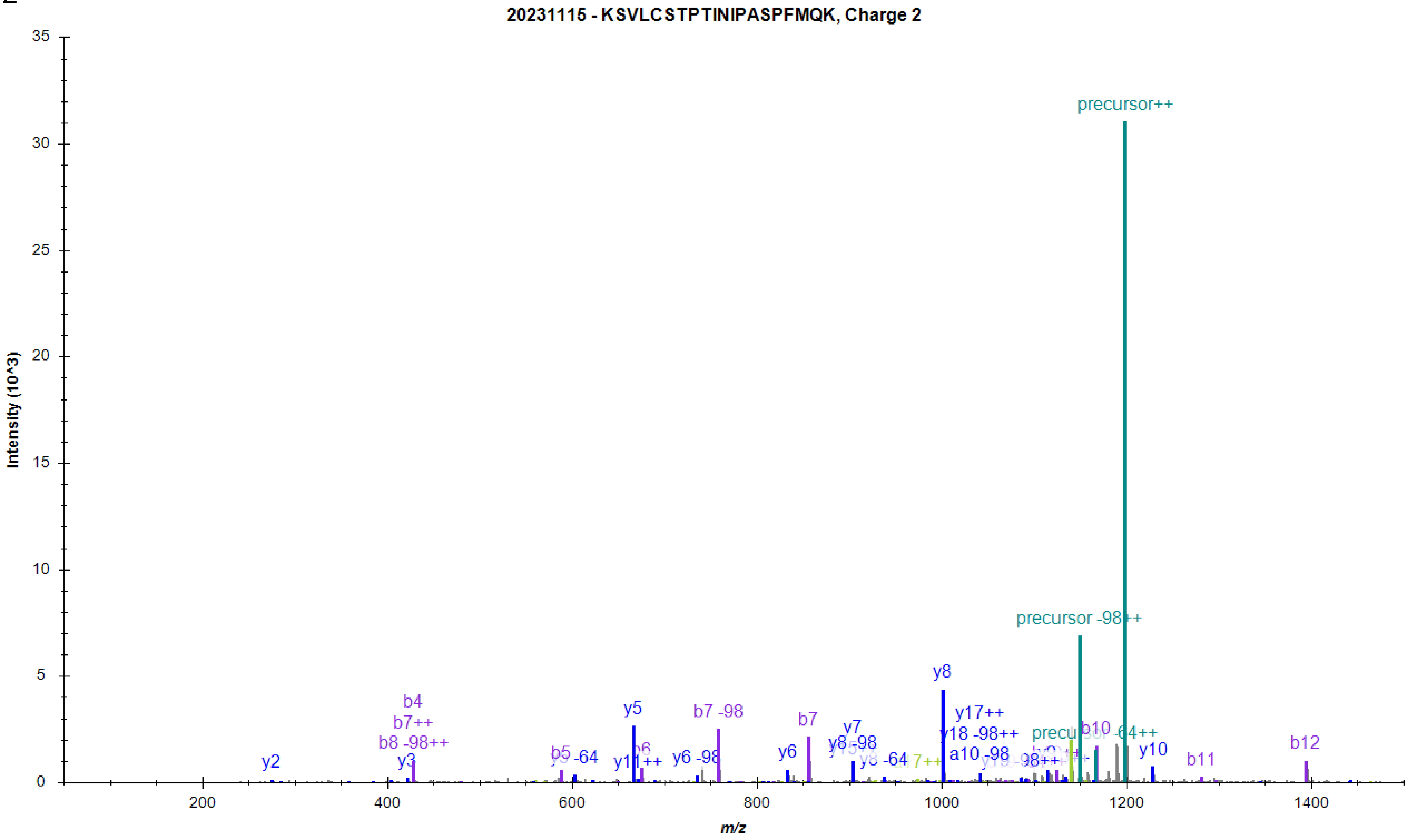
20231115 - KSVLCSTPTINIPASPFMQK, Charge 2
Intensity (10^3)
m/z
precursor++
precursor -98++
y8
y17++
y18 -98++
a10 -98
y10
b12
b11
b7
b7 -98
y5
y6
y6 -98
y7
y8 -98
y8 -64
y11++
b4
b7++
b8 -98++
y3
y2
b5
y5 -64
b10
0
5
10
15
20
25
30
35
200
400
600
800
1000
1200
1400

PBK_T24-S32_2

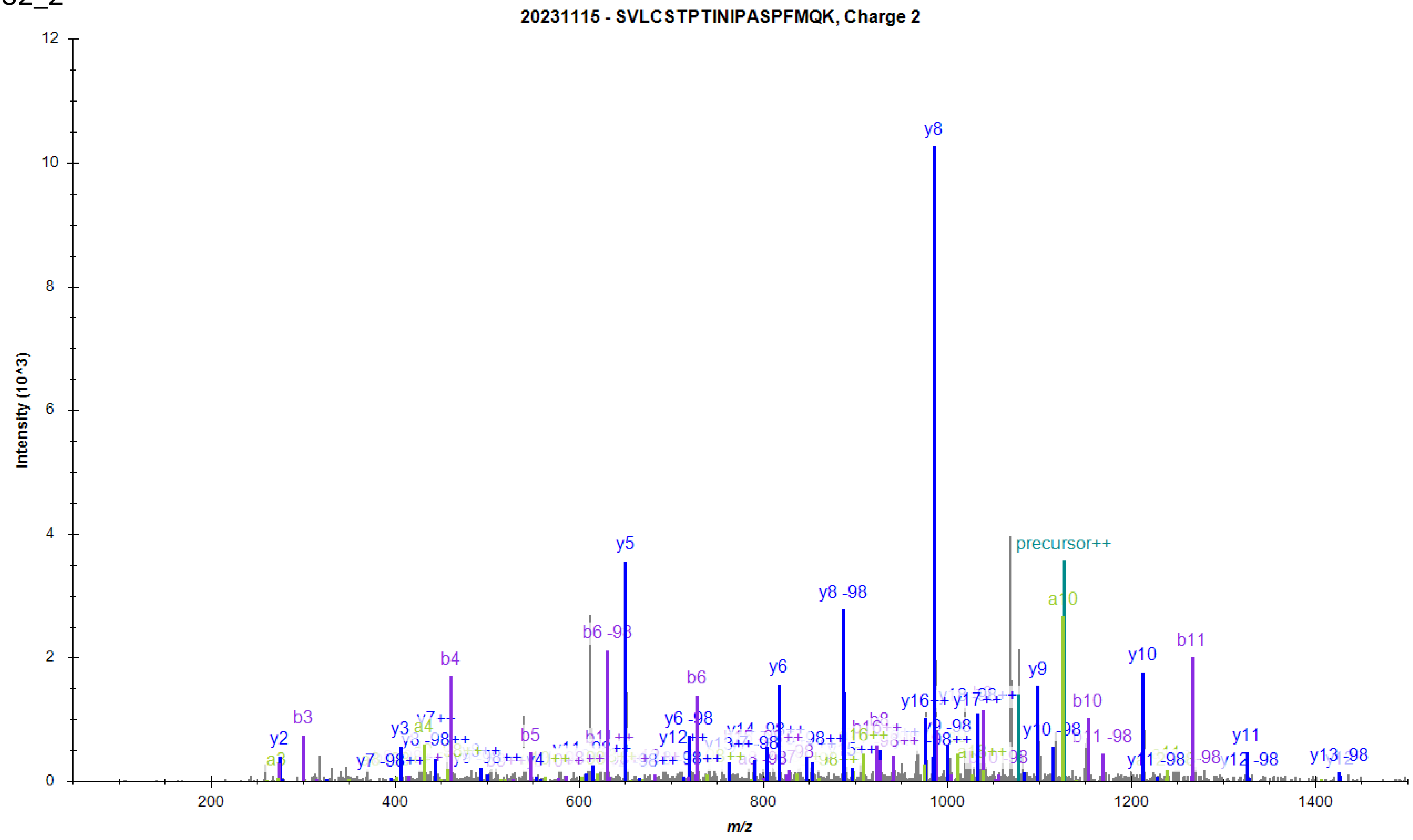

20231115 - SVLCSTPTINIPASPFMQK, Charge 2
Intensity (10^3)
m/z
y8
y5
y8 -98
precursor++
a10
b6 -98
b4
b11
y10
y9
y6
b6
b10
b3
y2
y11
y13 -98

PCM-1-S93

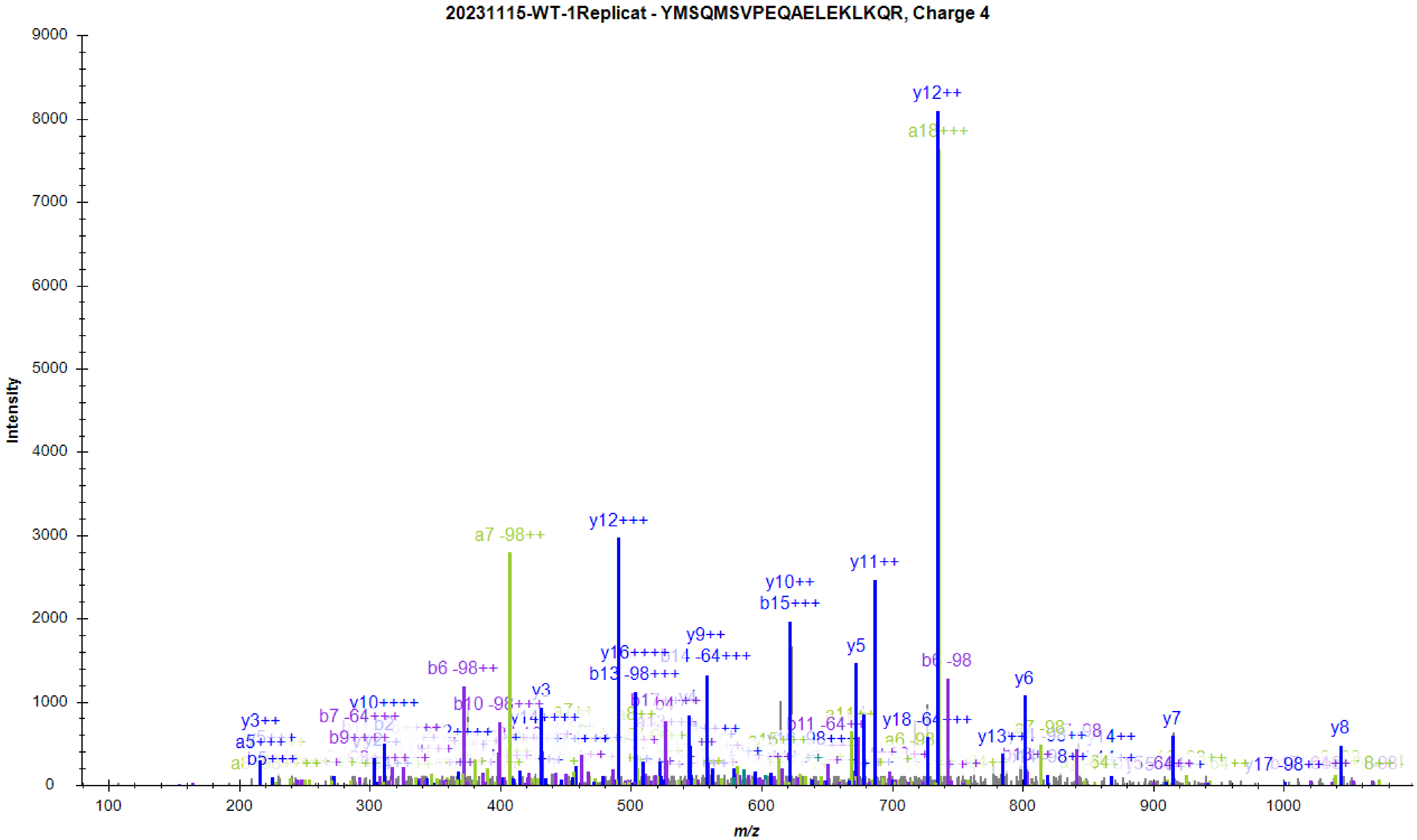

20231115-WT-1Replicat - YMSQMSVPEQAELEKLKQR, Charge 4
Intensity
m/z
y12++
a18+++
y12+++
a7 -98++
y11++
y10++
b15+++
y9++
y5
y6
y7
y8
y3
b6 -98++
b6 -98
y13++
y18 -64+++
b14 -64+++
b13 -98+++
b10 -98+++
b7 -64+++
y10++++
y3++
a5+++
b5+++
b9+++
b11 -64++

PCM-1-S65

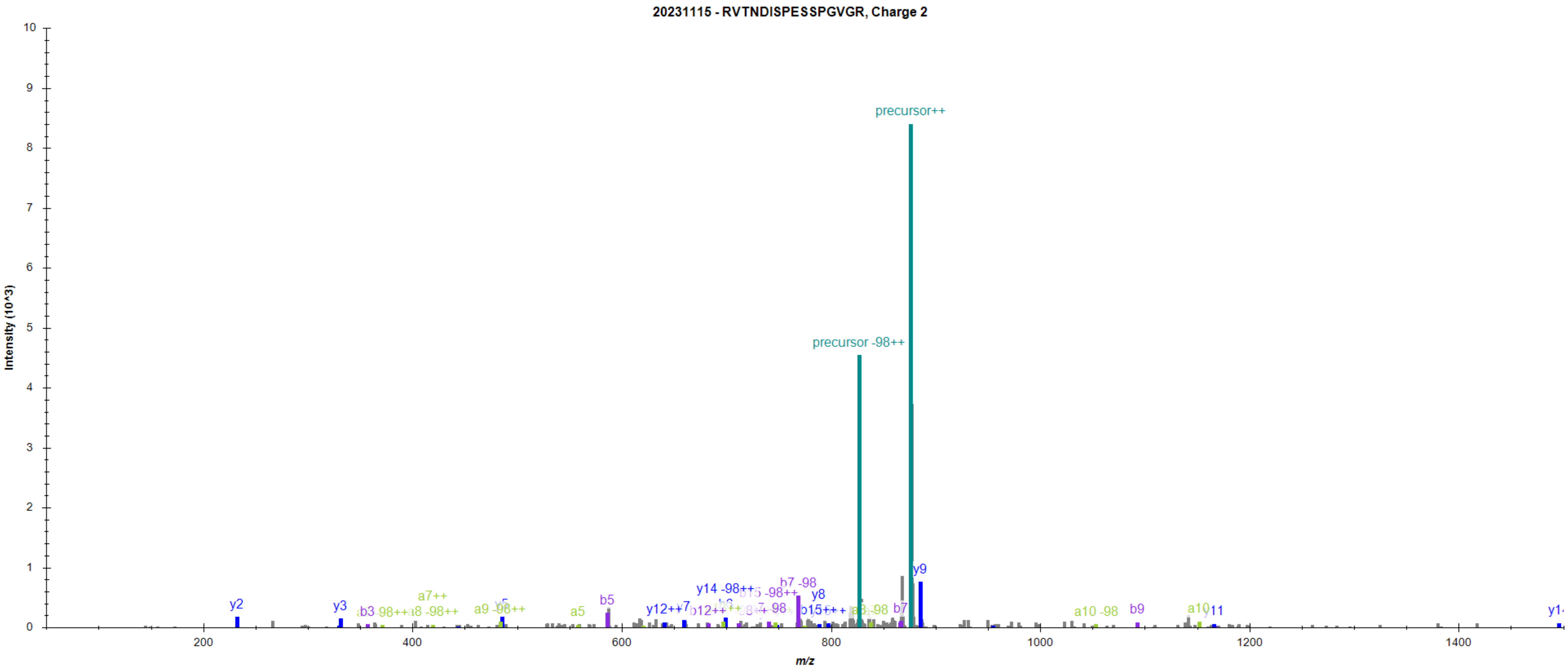

20231115 - RVTNDISPESSPGVGR, Charge 2
Intensity (10^3)
m/z
precursor++
precursor -98++
y2
y3
b3
a7++
a5
b5
y14 -98++
b7 -98
y8
y9
b7
a10 -98
b9
y11

PCM-1-S65-S68

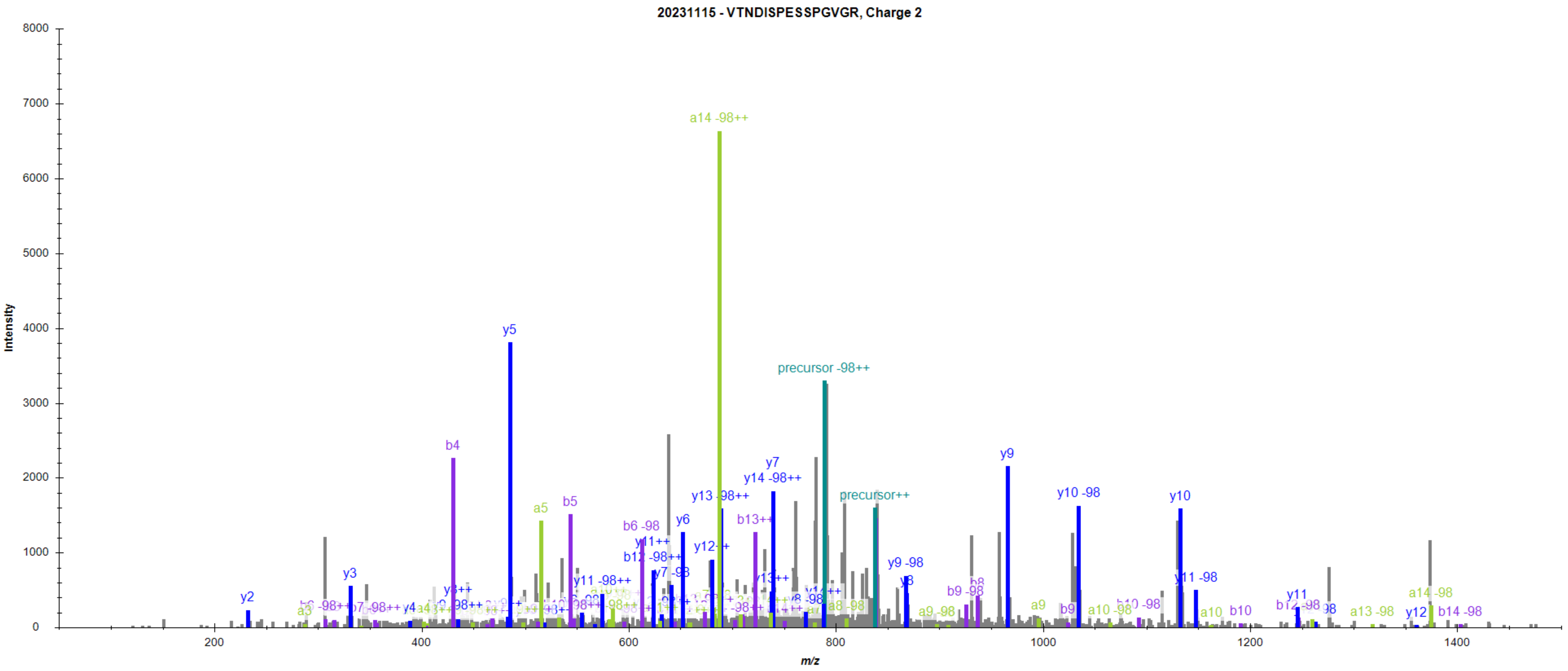
20231115 - VTNDISPESSPGVGR, Charge 2
Intensity
m/z
8000
7000
6000
5000
4000
3000
2000
1000
0
200
400
600
800
1000
1200
1400
a14 -98++
y5
precursor -98++
b4
y7
y14 -98++
y9
y10 -98
y10
y13 -98++
precursor++
a5
b5
b13++
b6 -98
y6
y12++
y11++
b12 -98++
y9 -98
y11 -98
y11 -98++
y3
y2
y11
b9 -98
a9
b9
a10 -98
a10
b10
a13 -98
y12
b14 -98
a14 -98
a9 -98
a8 -98
y4

PRKD1-S205

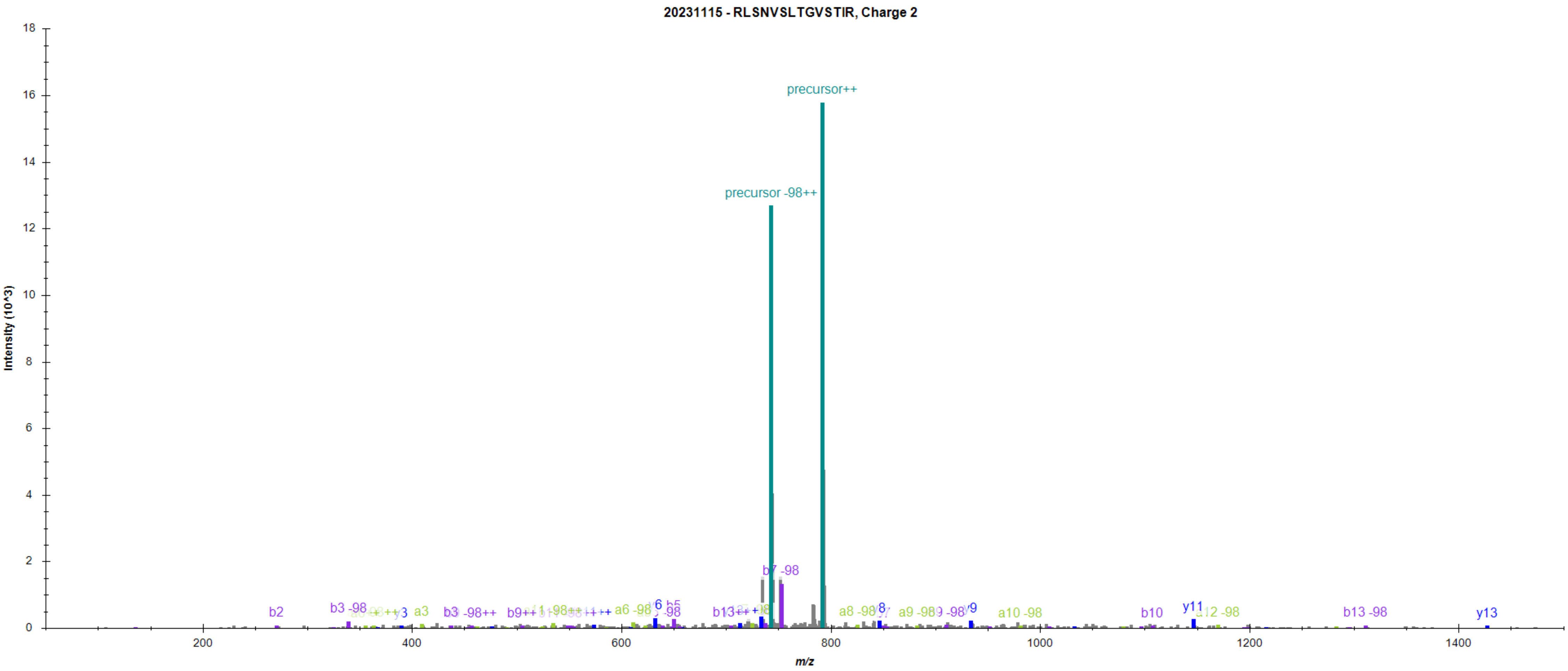

20231115 - RLSNVSLTGVSTIR, Charge 2
Intensity (10^3)
m/z
precursor++
precursor -98++
b7 -98
b2
b3 -98
a3
b3 -98++
b9++
a6 -98
b13++
a8 -98
a9 -98
a10 -98
b10
y11
b13 -98
y13

PRKD1-S205-S208

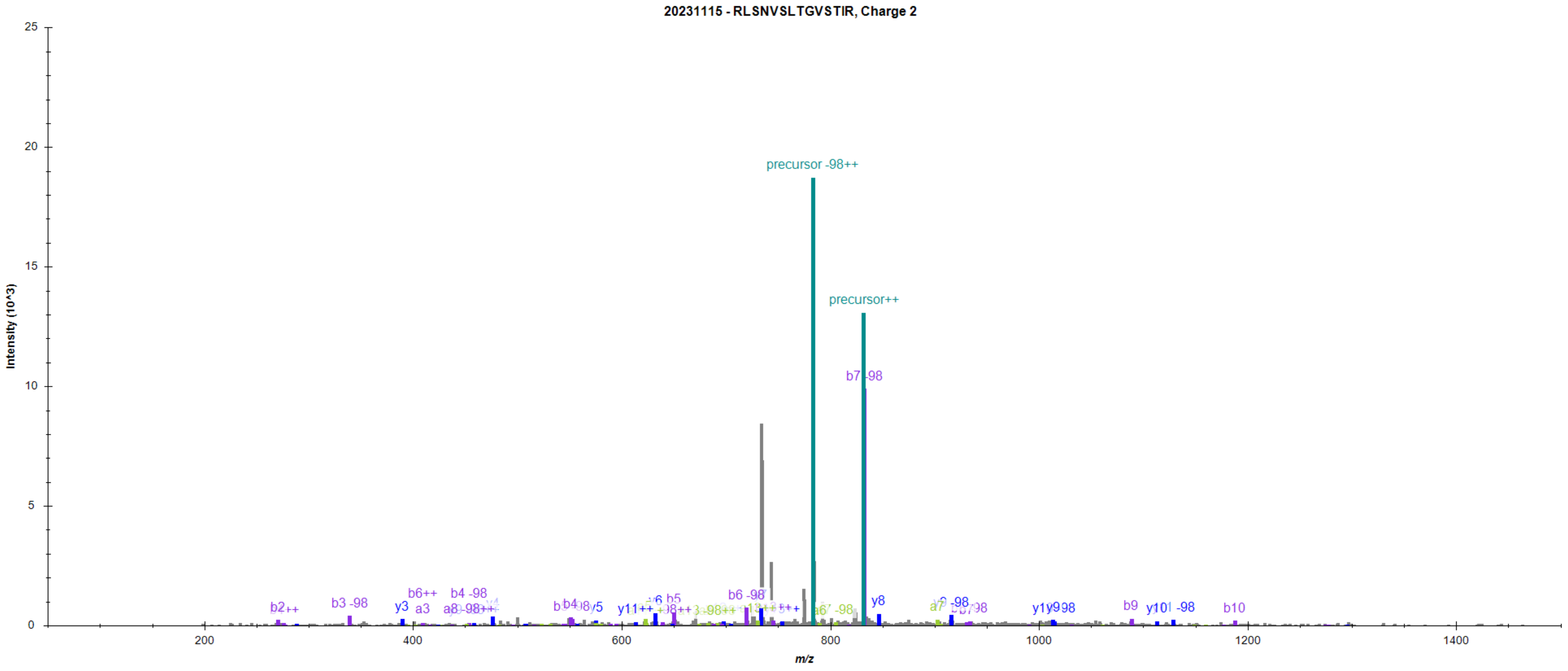

PRKD1-S397

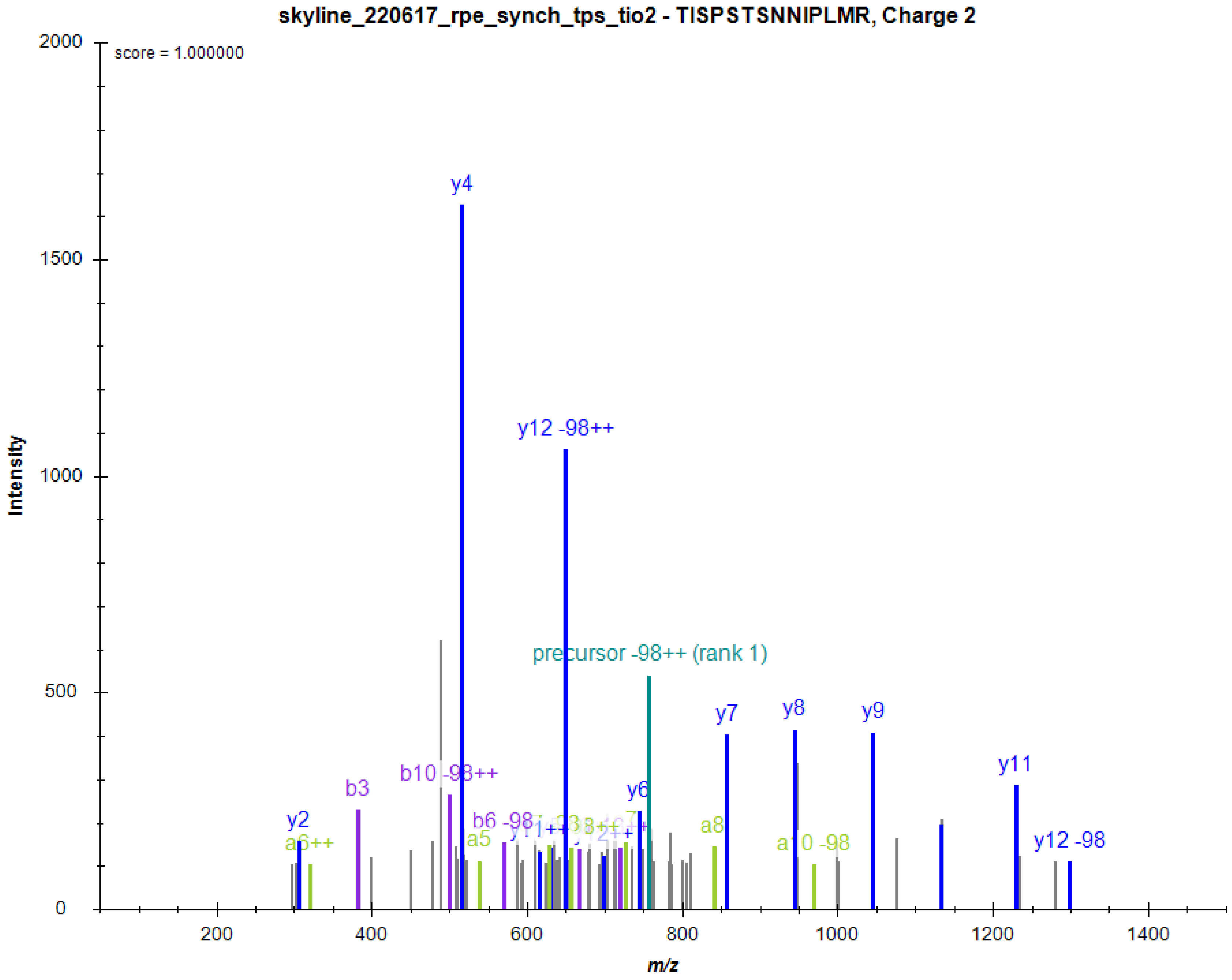
skyline_220617_rpe_synch_tps_tio2 - TISPSTSNNIPLMR, Charge 2
score = 1.000000
Intensity
m/z
y4
y12 -98++
precursor -98++ (rank 1)
y7
y8
y9
y11
y12 -98
y2
b3
b10 -98++
y6
a5
a8

PRKD1-S742

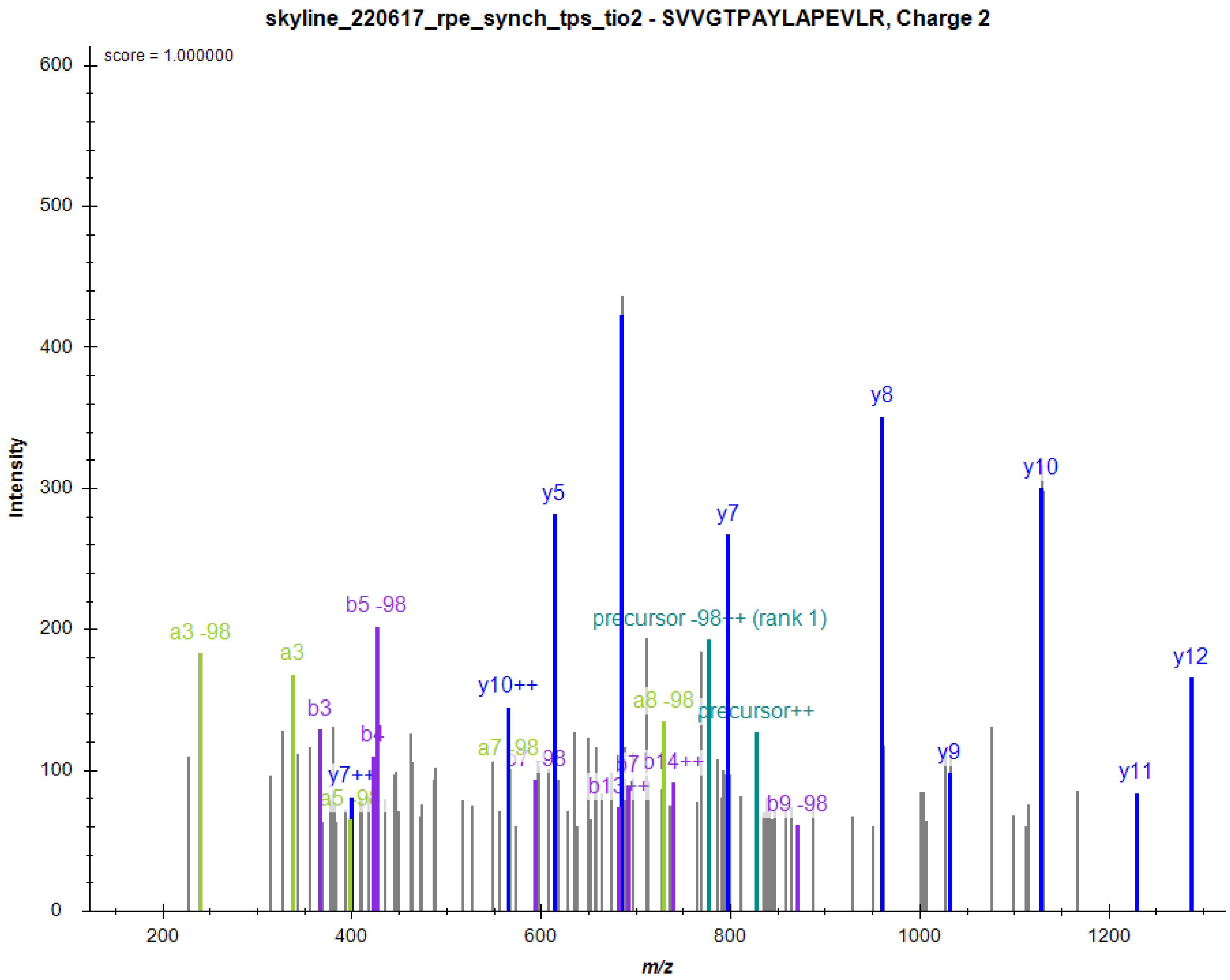

skyline_220617_rpe_synch_tps_tio2 - SVVGTPAYLAPEVLR, Charge 2
score = 1.000000
Intensity
m/z
y8
y10
y5
y7
precursor -98++ (rank 1)
b5 -98
a3 -98
a3
y12
y10++
a8 -98
precursor++
b3
b4
y9
y11
a7 -98
y7++
b7
b14++
b13++
b9 -98

PRKD2-S197

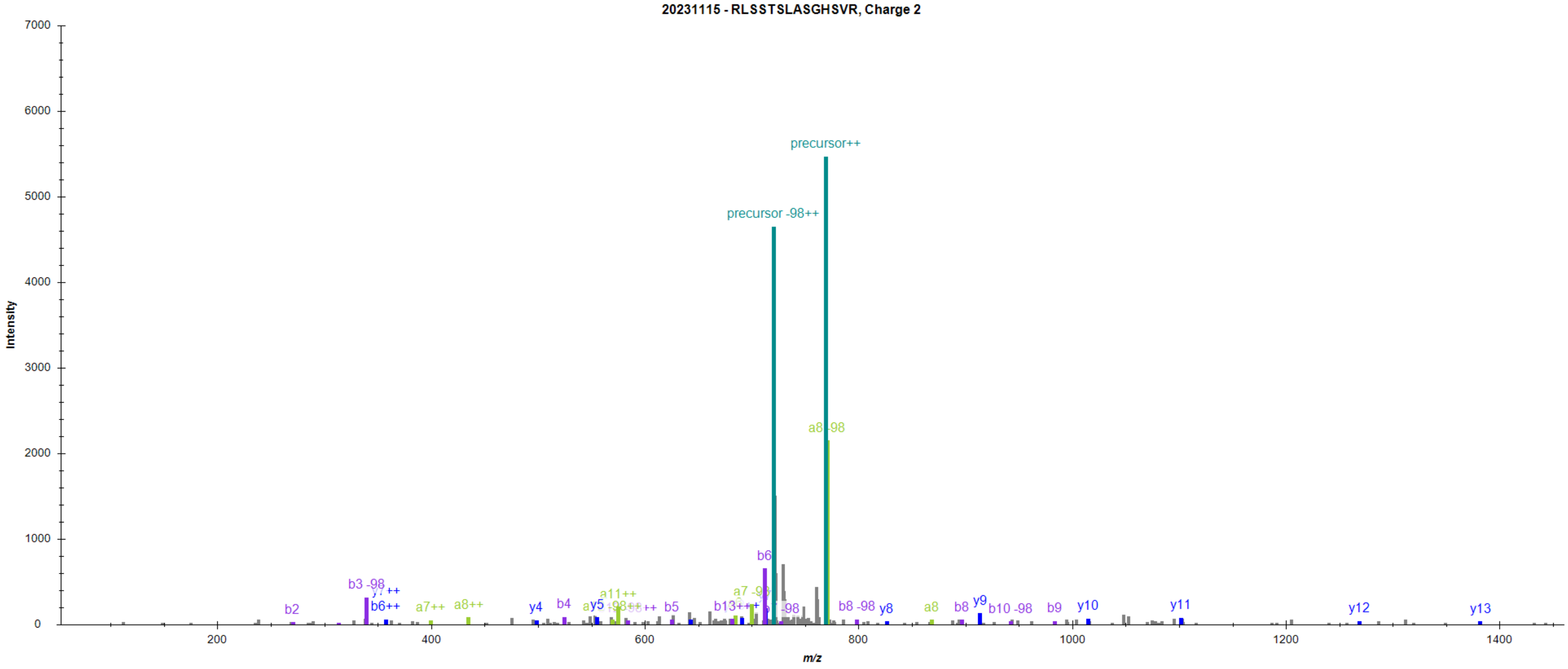
20231115 - RLSSTSLASGHSVR, Charge 2
Intensity
m/z
precursor++
precursor -98++
a8 -98
b6
b3 -98
b2
b6++
a7++
a8++
y4
b4
y5
a11++
b5
b13++
a7 -98
b8 -98
y8
a8
b8
y9
b10 -98
b9
y10
y11
y12
y13

PRKD2-S197-S198

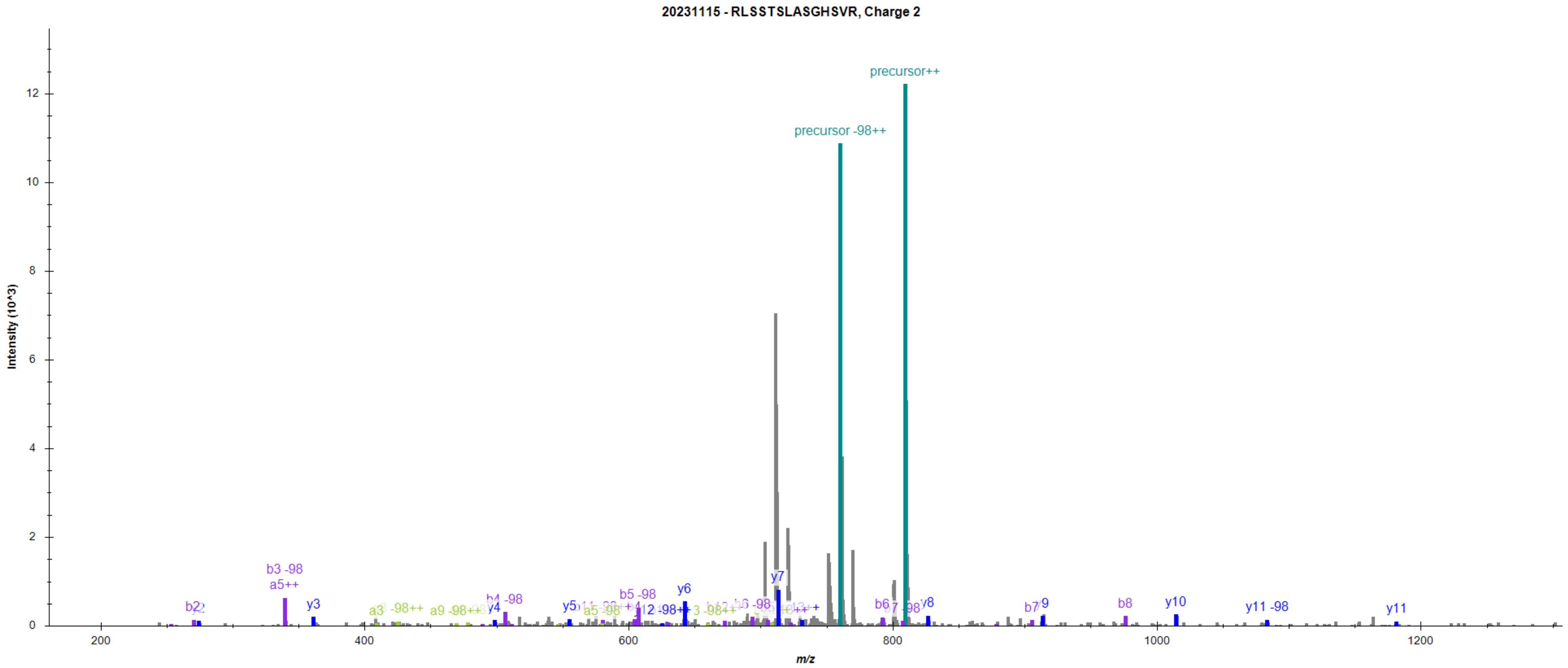

RAB34-S241

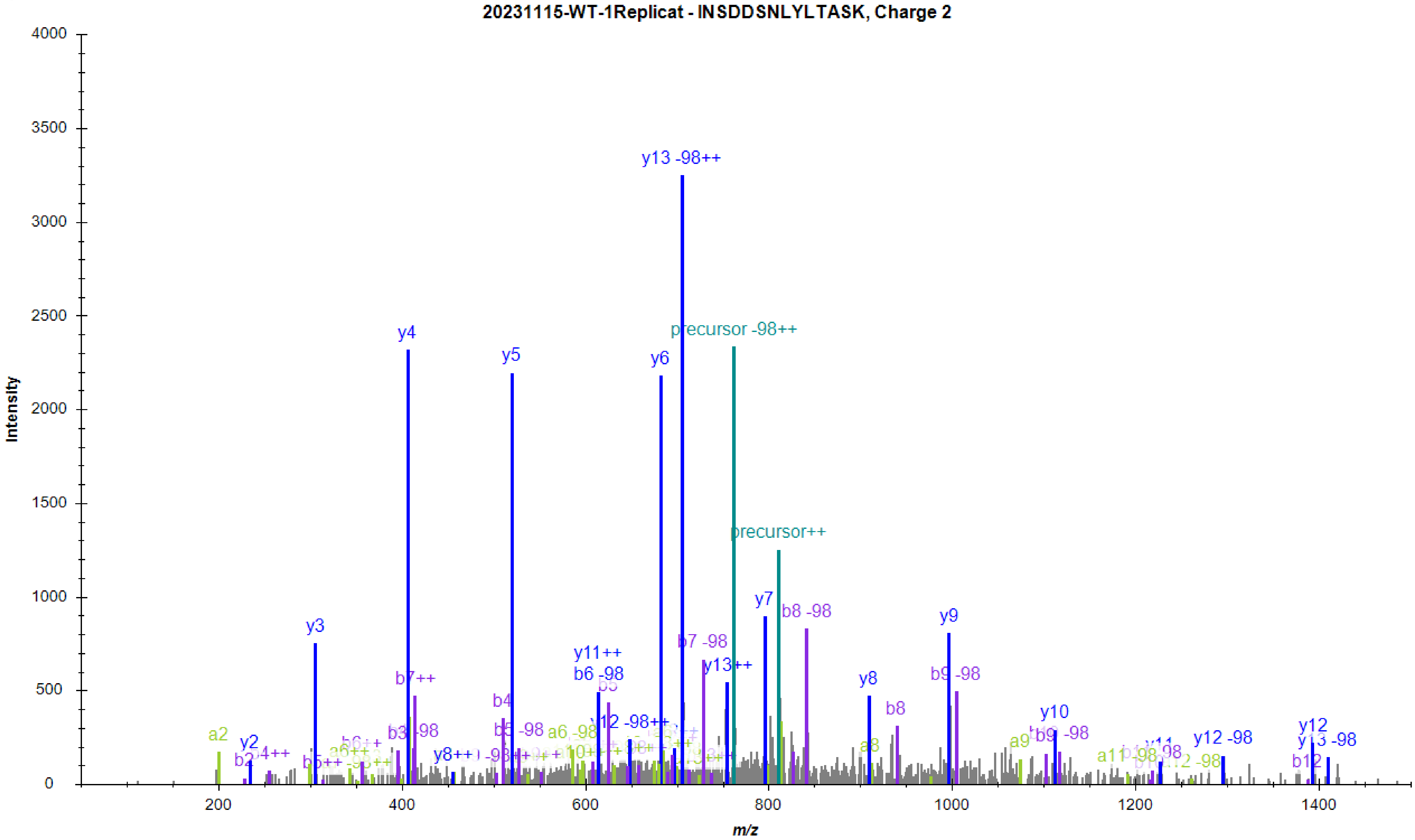

20231115-WT-1Replicat - INSDDSNLYLTASK, Charge 2
Intensity
m/z
y13 -98++
precursor -98++
precursor++
y4
y5
y6
y3
y7
b8 -98
y9
b7 -98
y11++
b6 -98
y13++
b7++
b9 -98
y8
b8
b4
y10
b3 -98
b5 -98
a6 -98
y12 -98
y12
y13 -98
a2
y2
b2
b5++
y8++
a8
a9
a11 -98
b12

RB1CC1_S243

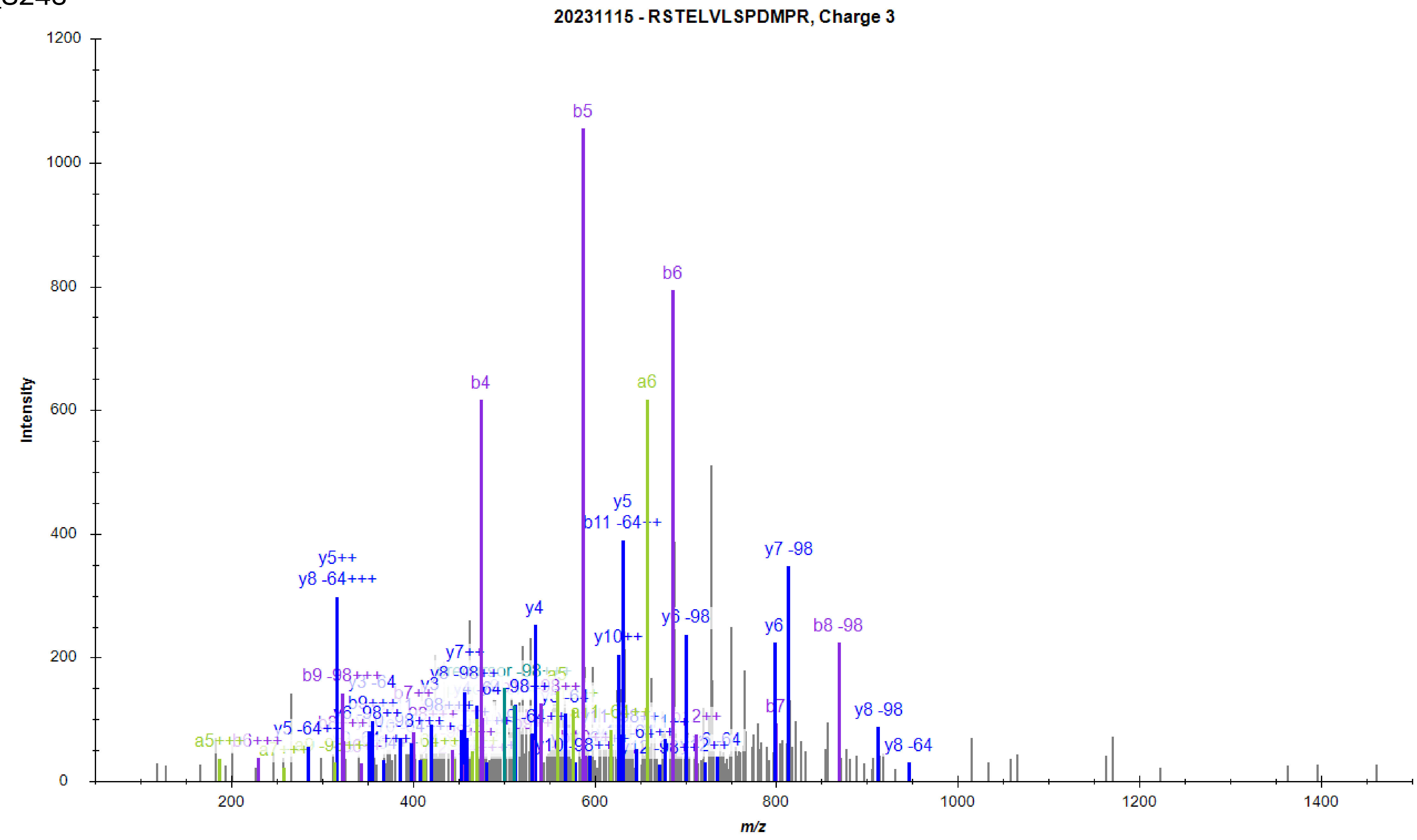
20231115 - RSTELVLSPDMPR, Charge 3
Intensity
m/z
b5
b6
b4
a6
y5
b11 -64++
y7 -98
y5++
y8 -64+++
y4
y6 -98
y6
b8 -98
y10++
y7++
b7
y8 -98
y8 -64

RB1CC1_S647

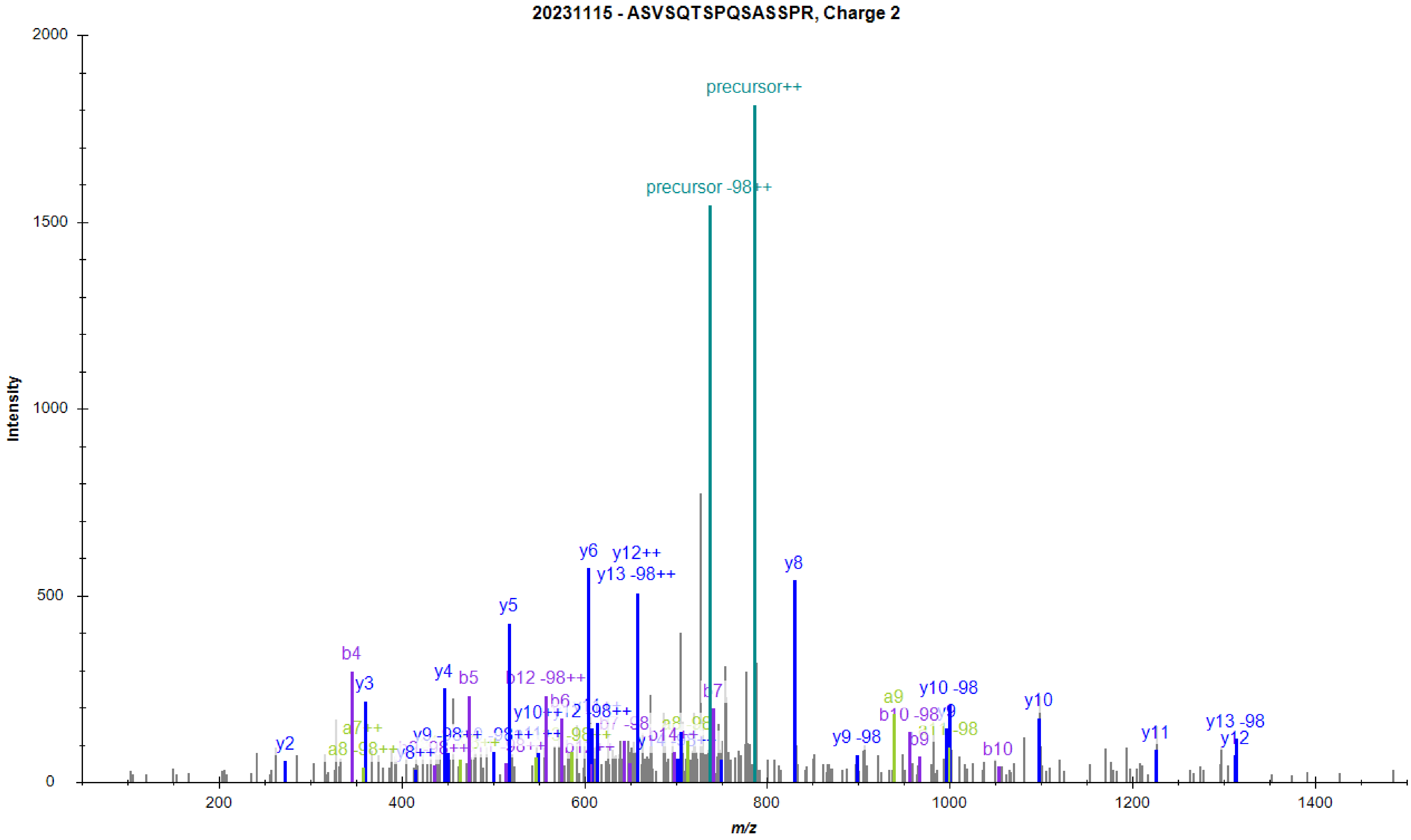
20231115 - ASVSQTSPQSASSPR, Charge 2
Intensity
m/z
precursor++
precursor -98++
y2
y3
b4
y4
b5
y5
b12 -98++
y6
y12++
y13 -98++
y8
a9
y10 -98
b10 -98
y10
b10
y11
y13 -98
y12
y9 -98
b9
b7
a7++
a8 -98++
0
500
1000
1500
2000
200
400
600
800
1000
1200
1400

RIPK1_S320

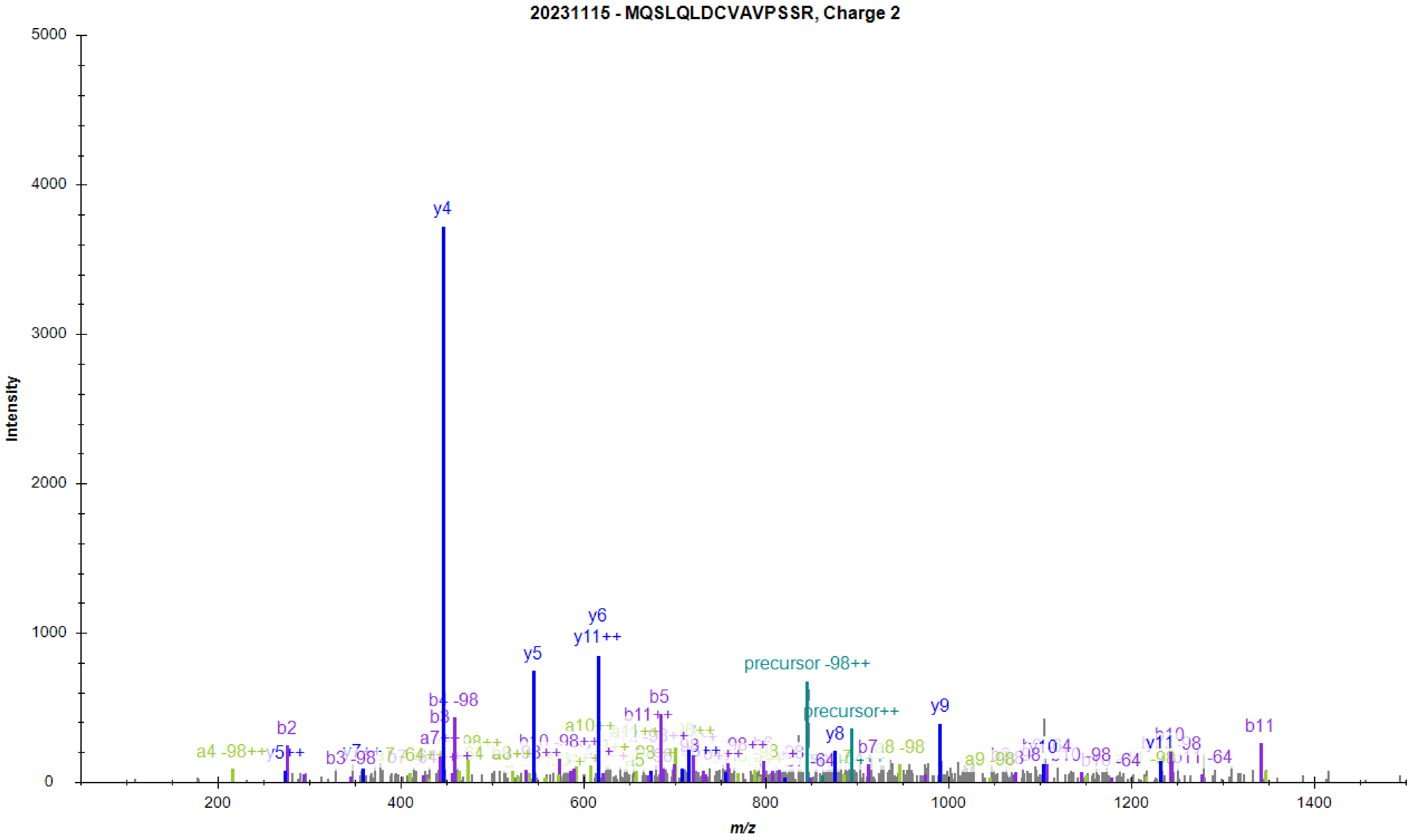

20231115 - MQSLQLDCVAVPSSR, Charge 2
Intensity
m/z
5000
4000
3000
2000
1000
0
200
400
600
800
1000
1200
1400
y4
y6
y11++
y5
precursor -98++
precursor++
y9
y8
b11
b5
b11++
b4 -98
b3
b2
a4 -98++
y5++
a10++
b7
a8 -98

RRAGC_S95

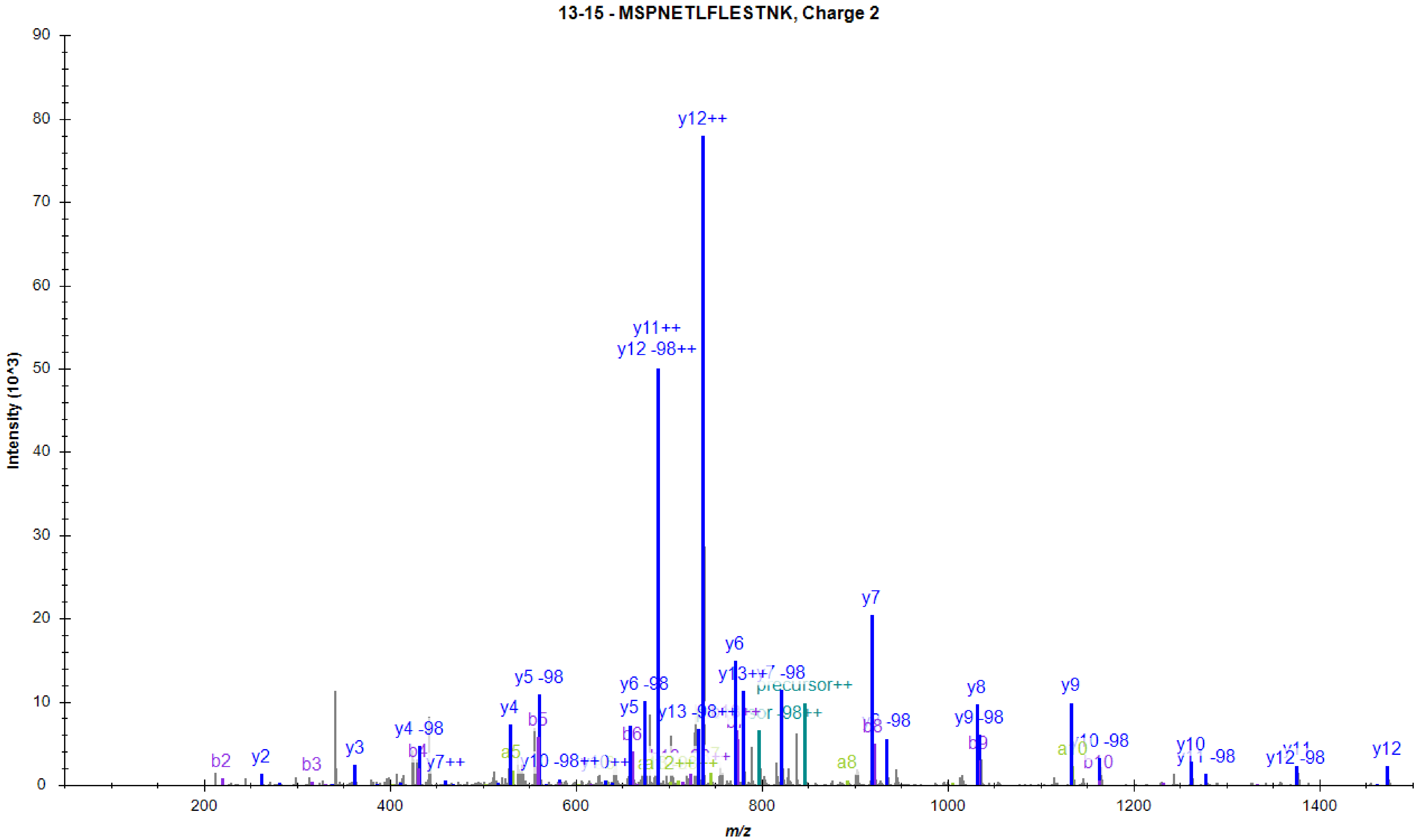
13-15 - MSPNETLFLESTNK, Charge 2
Intensity (10^3)
m/z
y12++
y11++
y12 -98++
y7
y6
y5 -98
y6 -98
y4
y5
y8
y9
y9 -98
y10
y12
y4 -98
y3
y2
b2
b3
b5
b6
b8
b9
b10
a5
a8
precursor++

Rubicon_S197

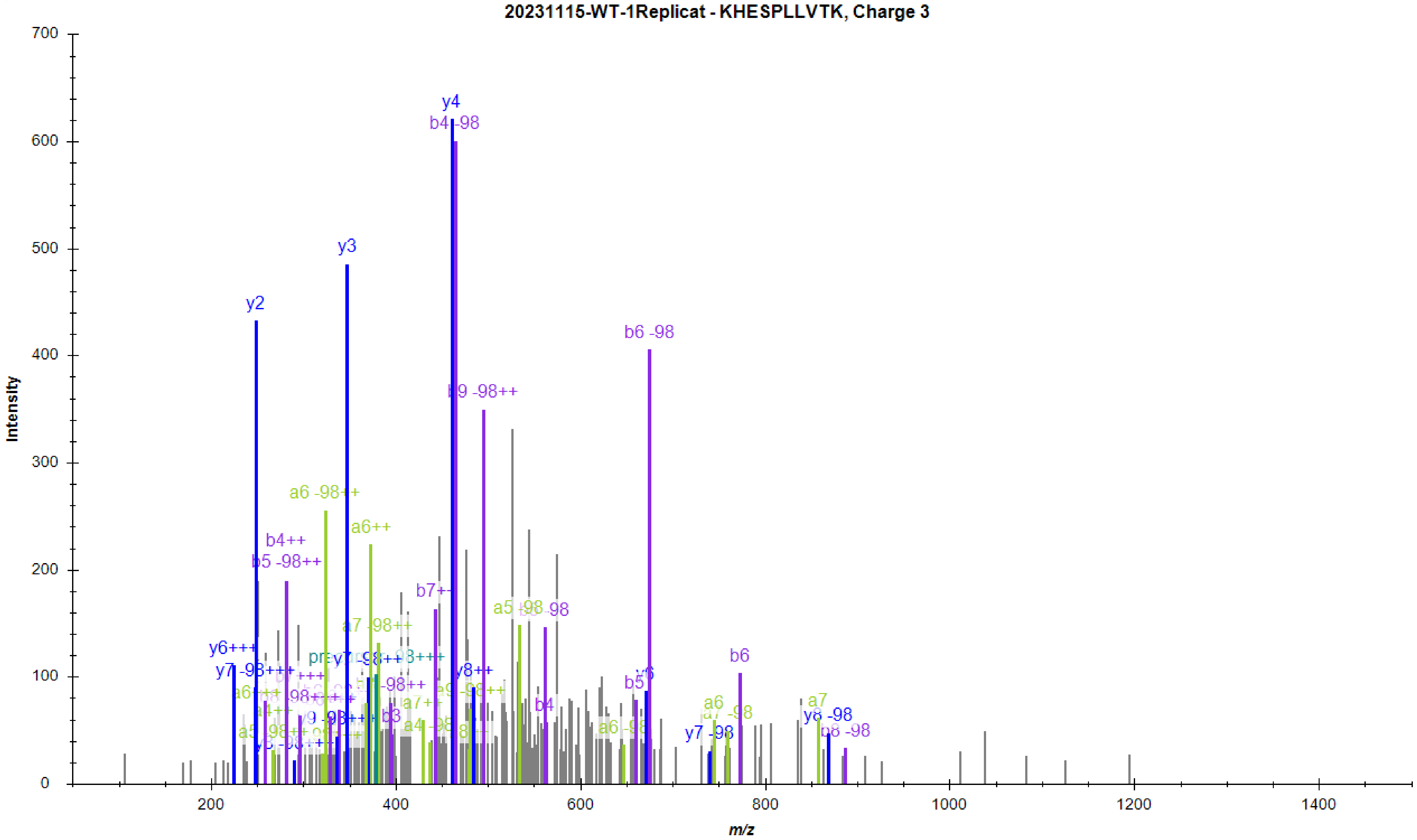

Rubicon_S388

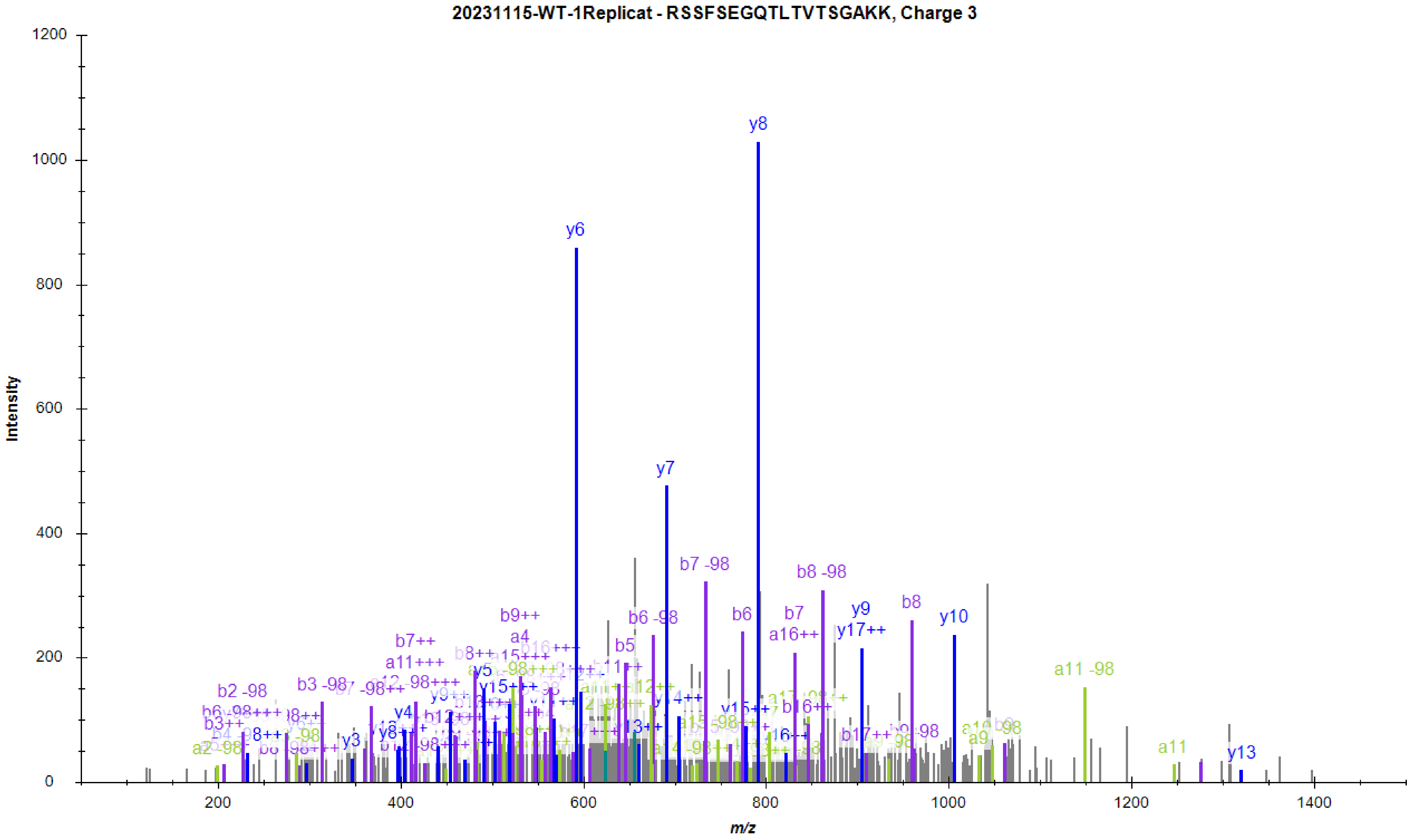

SQSTM1_T269_S269

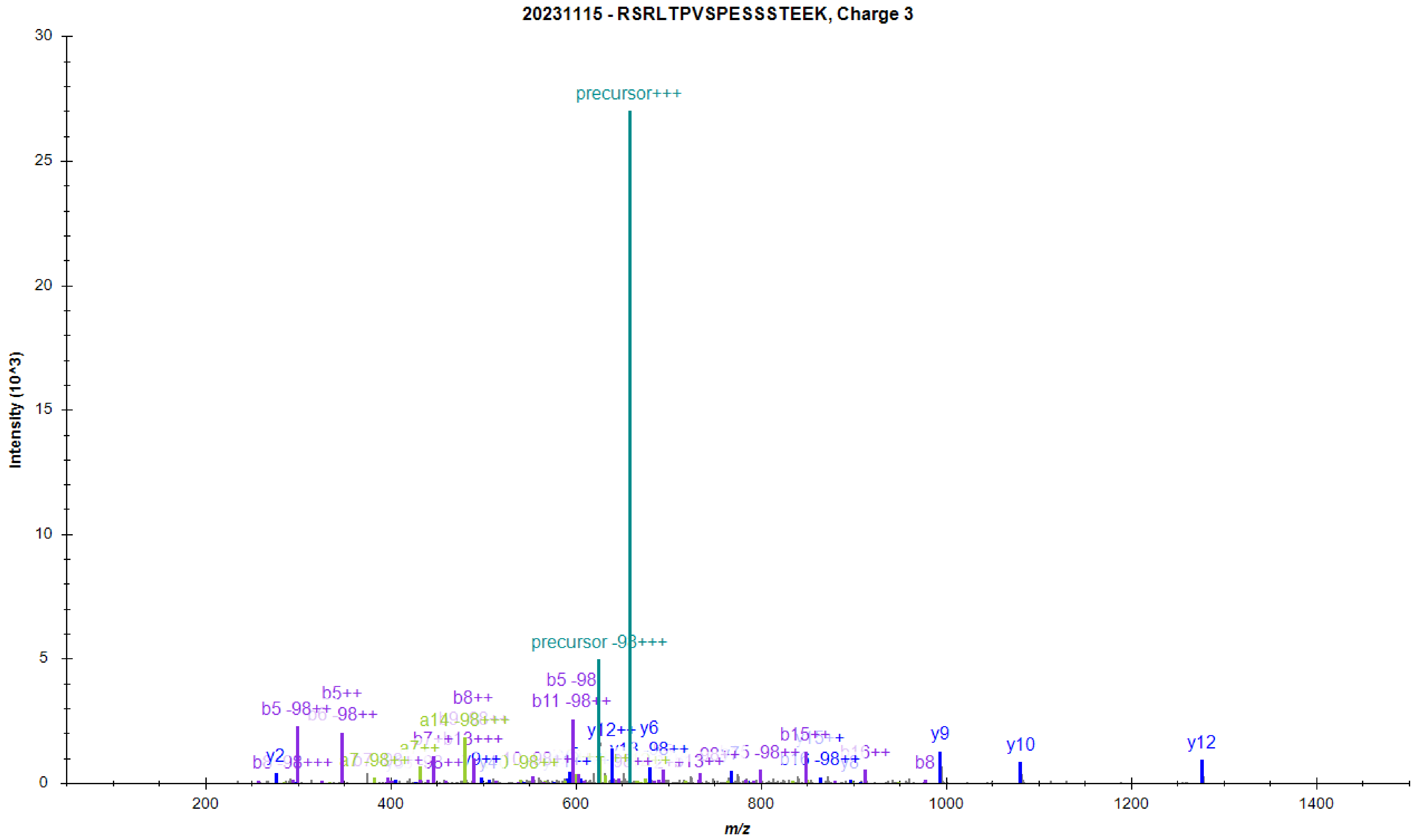

SQSTM1_T269_S272

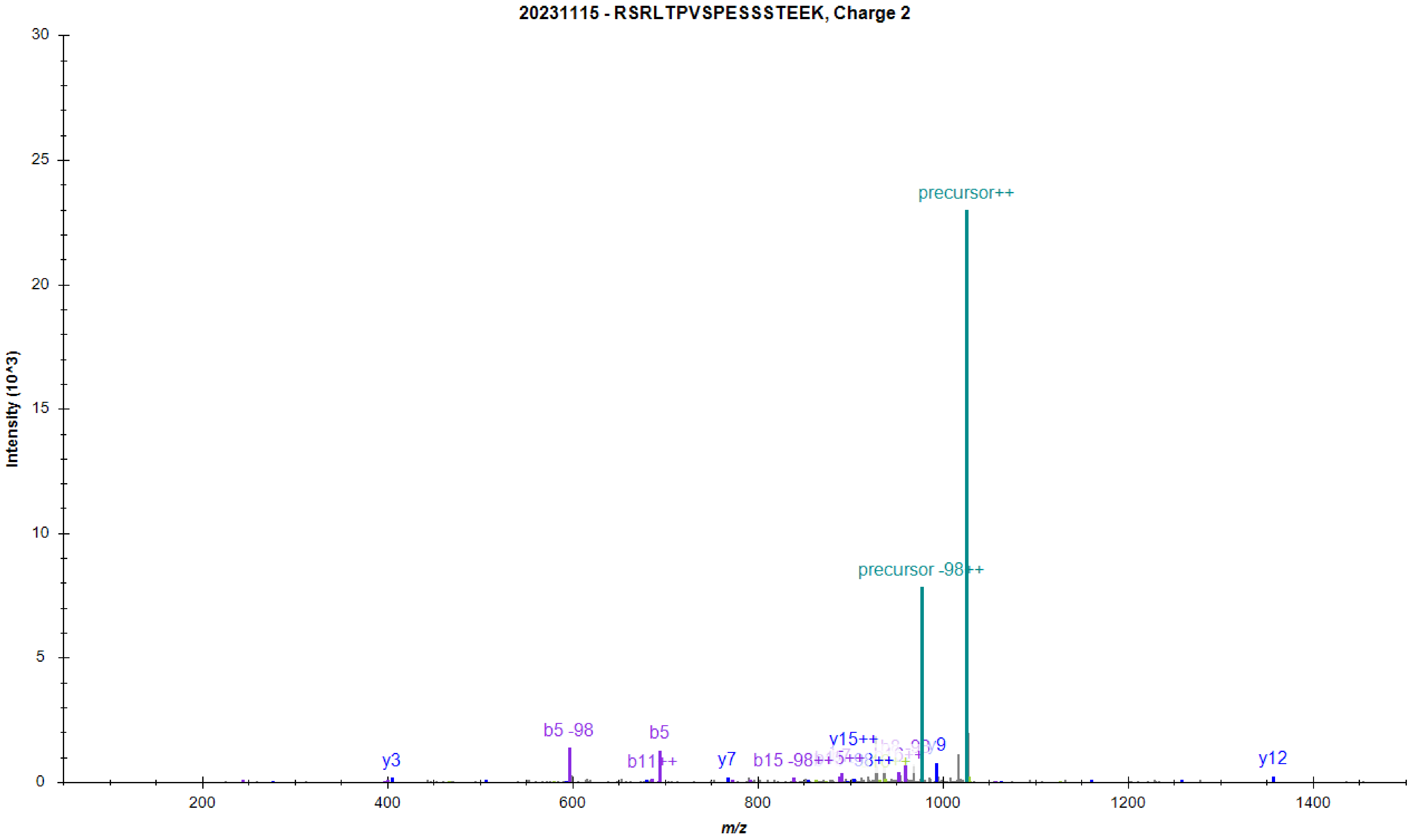

TMEM48_S406_T414

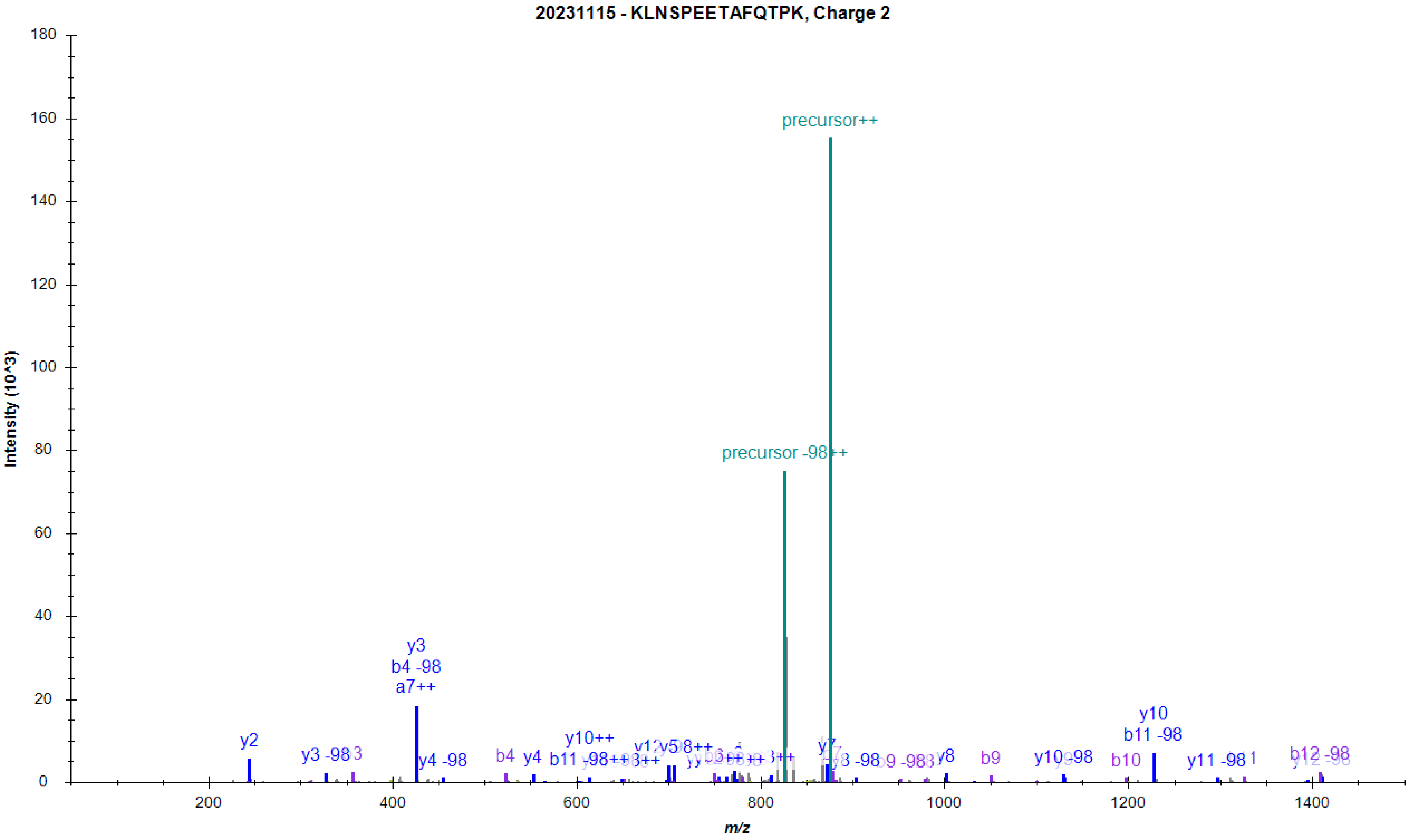

TMEM48_S471_S474

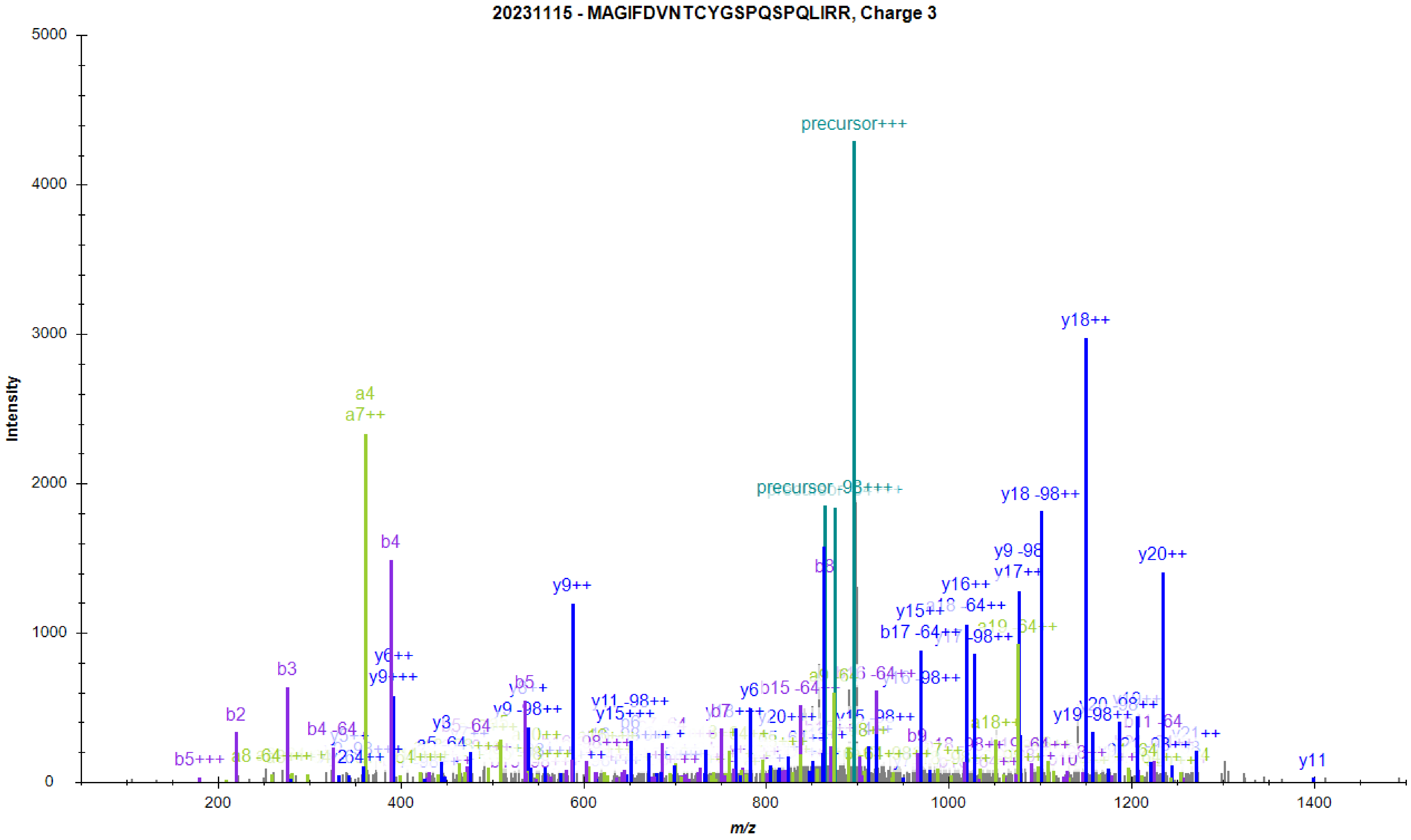

TPR_S1677

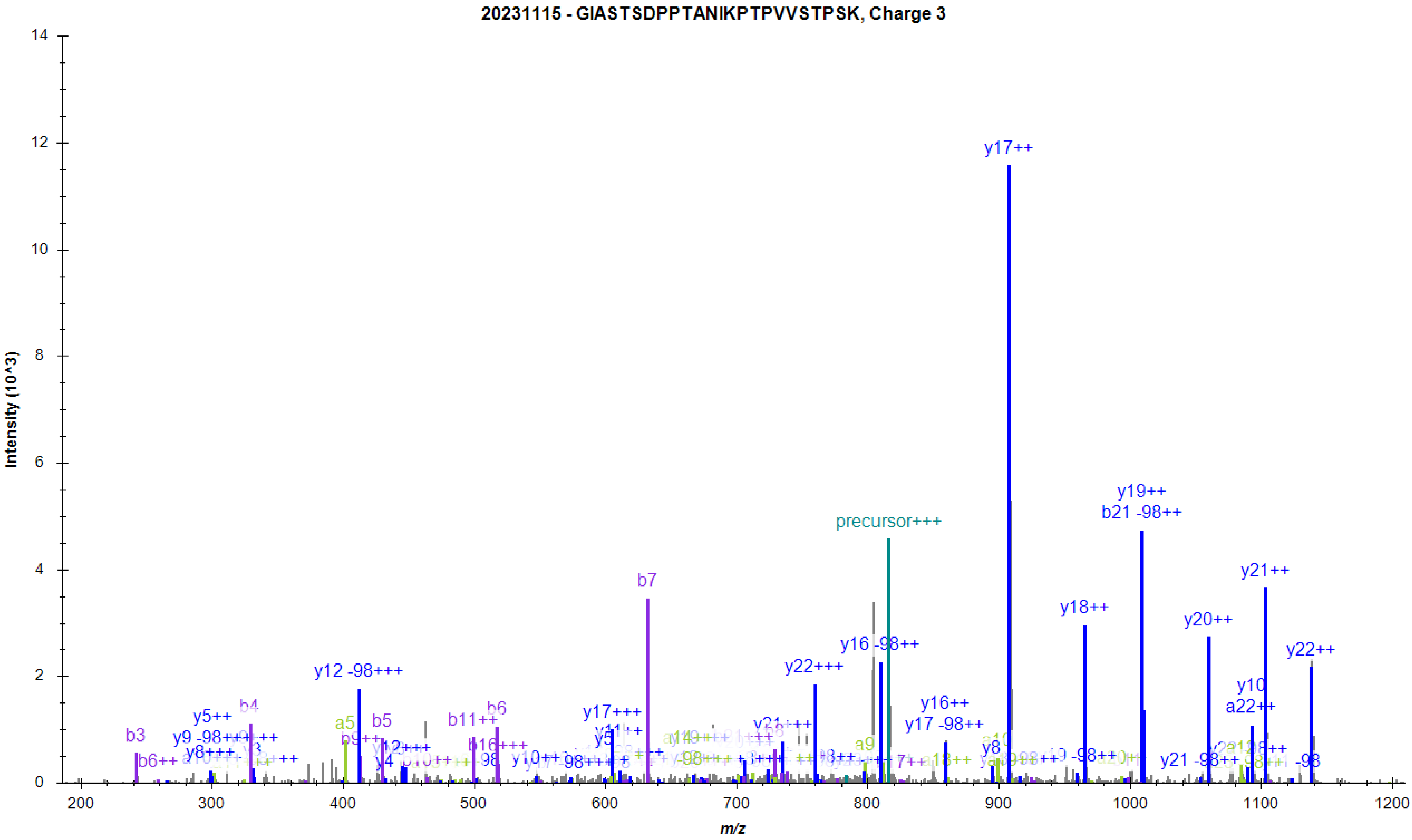
20231115 - GIASTSDPPTANIKPTPVVSTPSK, Charge 3
Intensity (10^3)
m/z
y17++
y19++
b21 -98++
precursor+++
y21++
y18++
y20++
y22++
b7
y16 -98++
y22+++
y12 -98+++
y16++
y17 -98++
y10
a22++
y17+++
b4
b5
a5
b6
b11++
b3
y5++
b6++
y8
a9

TPR_S2073

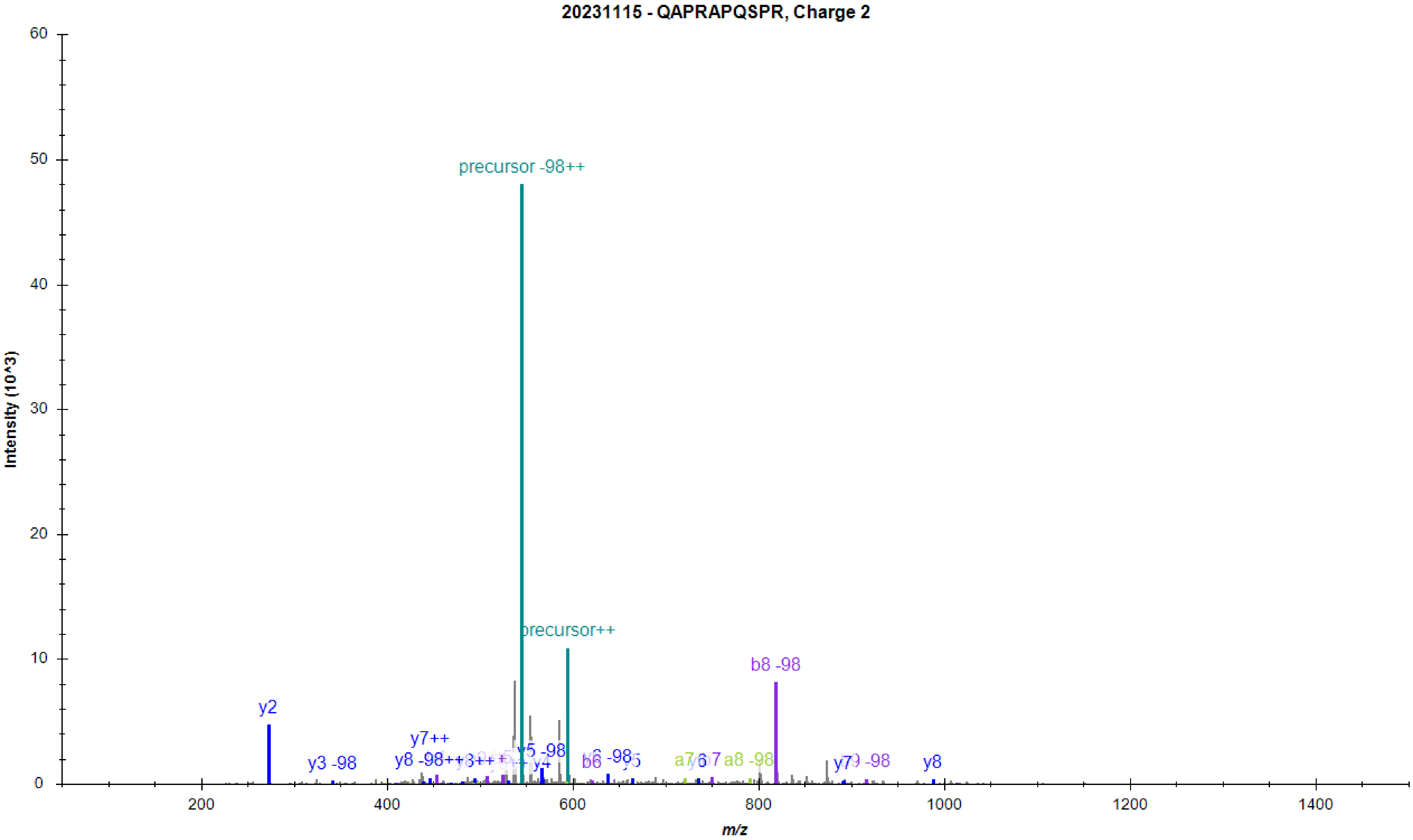

20231115 - QAPRAPQSPR, Charge 2
Intensity (10^3)
m/z
precursor -98++
precursor++
b8 -98
y2
y3 -98
y7++
y8 -98++
y5 -98
y4
b6
y6 -98
y5
y6
a8 -98
y7
y8

TRAPPC14-S517

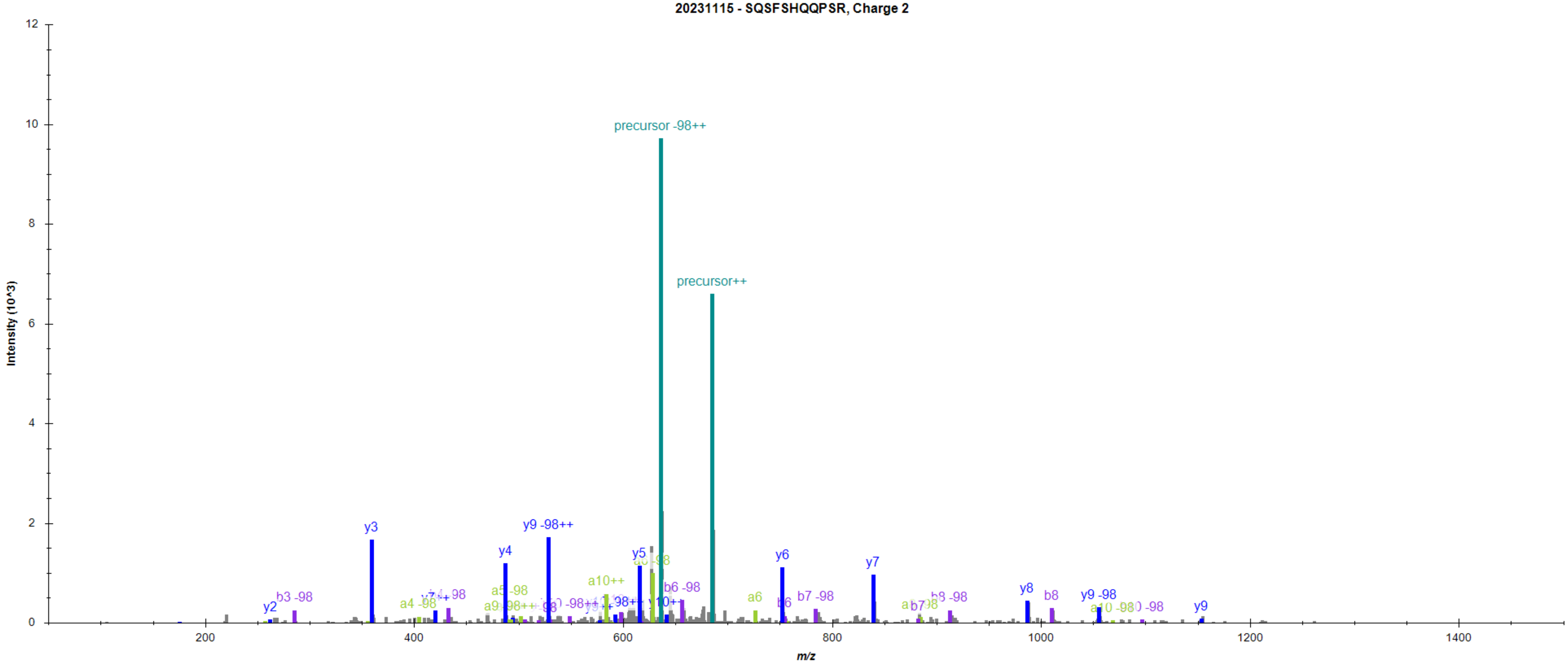

TSC2-S939

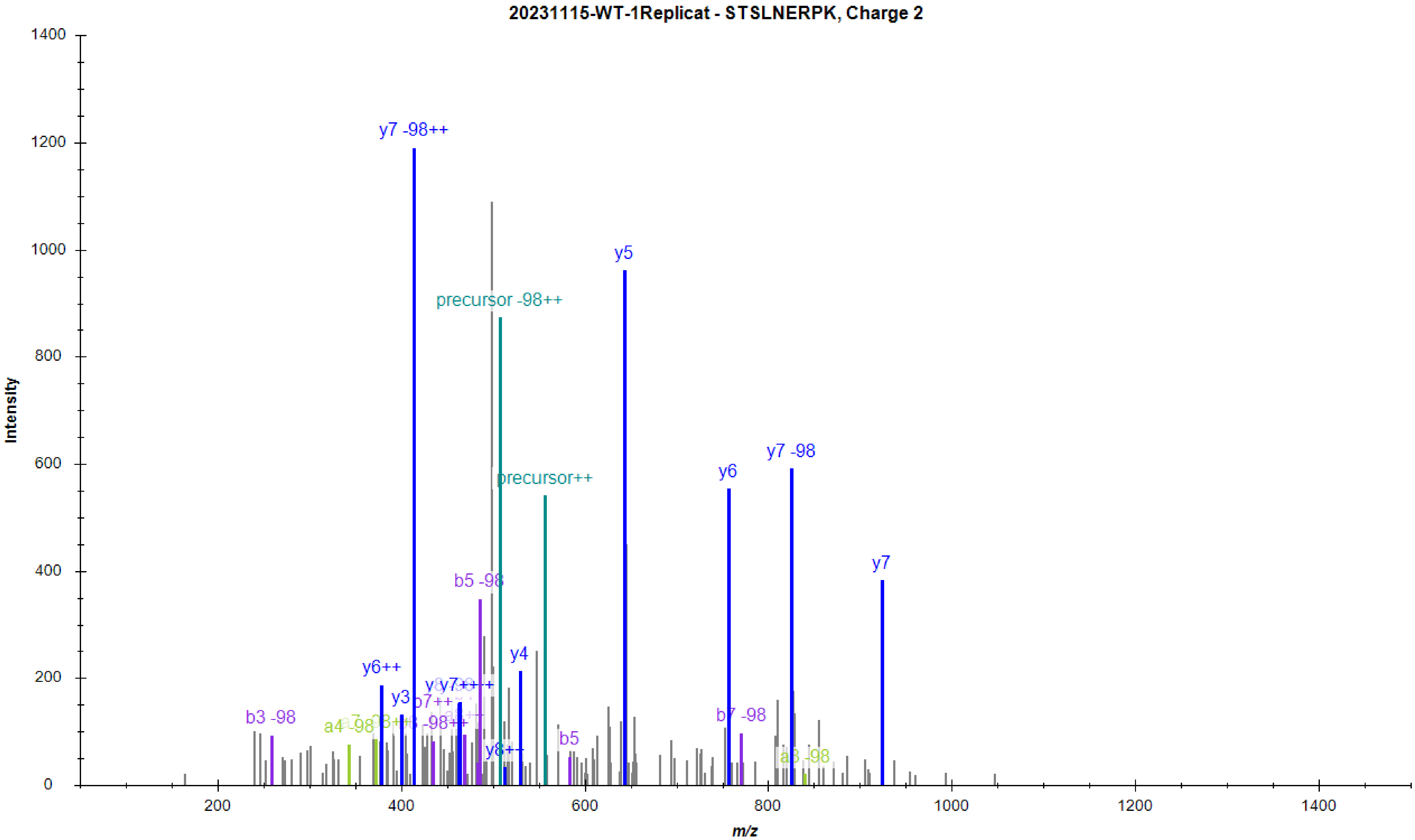
20231115-WT-1Replicat - STSLNERPK, Charge 2
Intensity
m/z
y7 -98++
precursor -98++
y5
precursor++
y6
y7 -98
y7
b5 -98
y4
y6++
y3
b3 -98
a4 -98
b5
b7 -98
y8++
a8 -98

TSC2-S1096

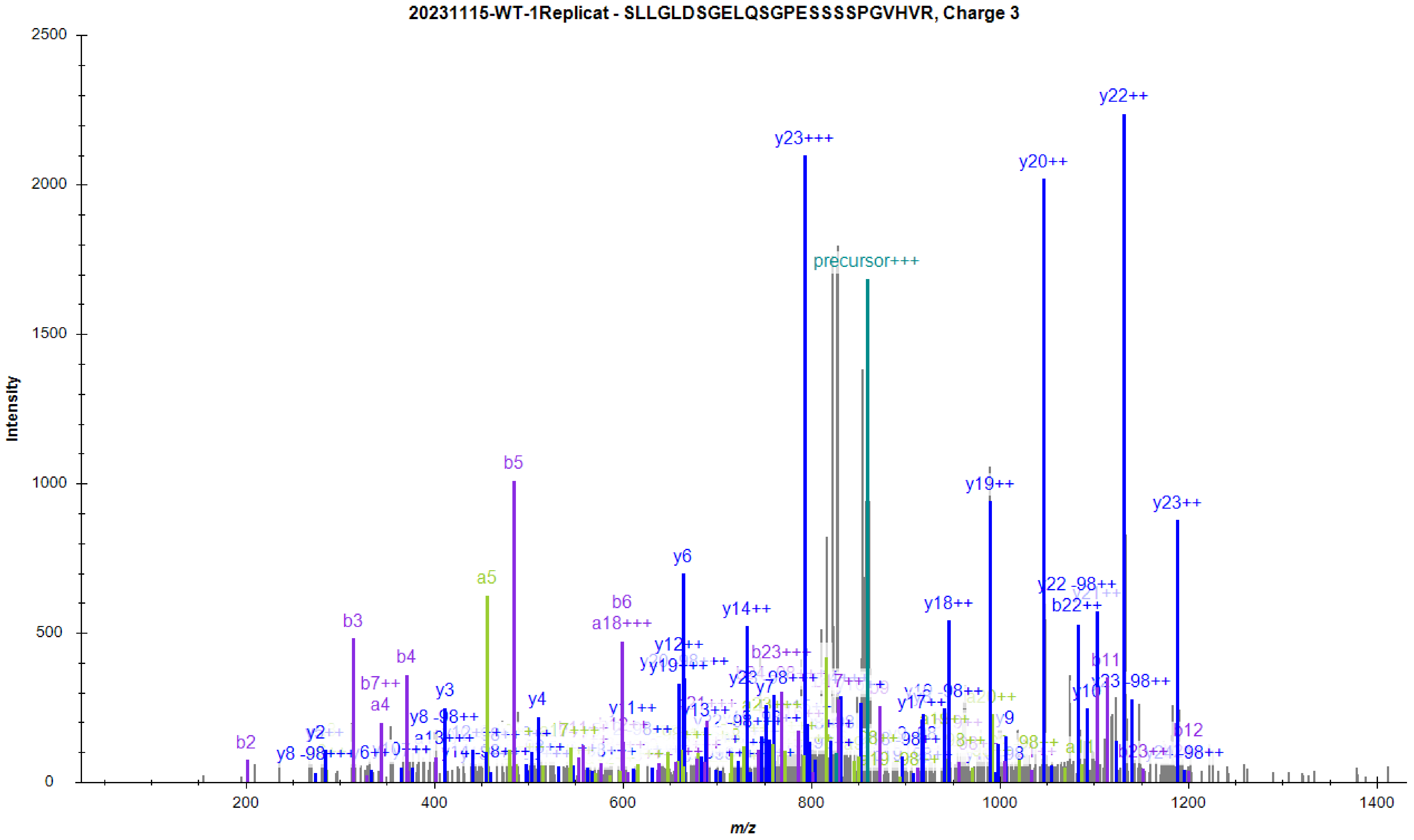
20231115-WT-1Replicat - SLLGLDSGELQSGPESSSSPGVHVR, Charge 3
Intensity
m/z
0
500
1000
1500
2000
2500
200
400
600
800
1000
1200
1400
y22++
y23+++
y20++
precursor+++
y19++
y23++
b5
y6
a5
y14++
y18++
b6
a18+++
b3
b4
y3
y4
b2
y2++
y12++
b23+++
y22 -98++
b22++
b11
b12
y9
y10

TSC2-S1155

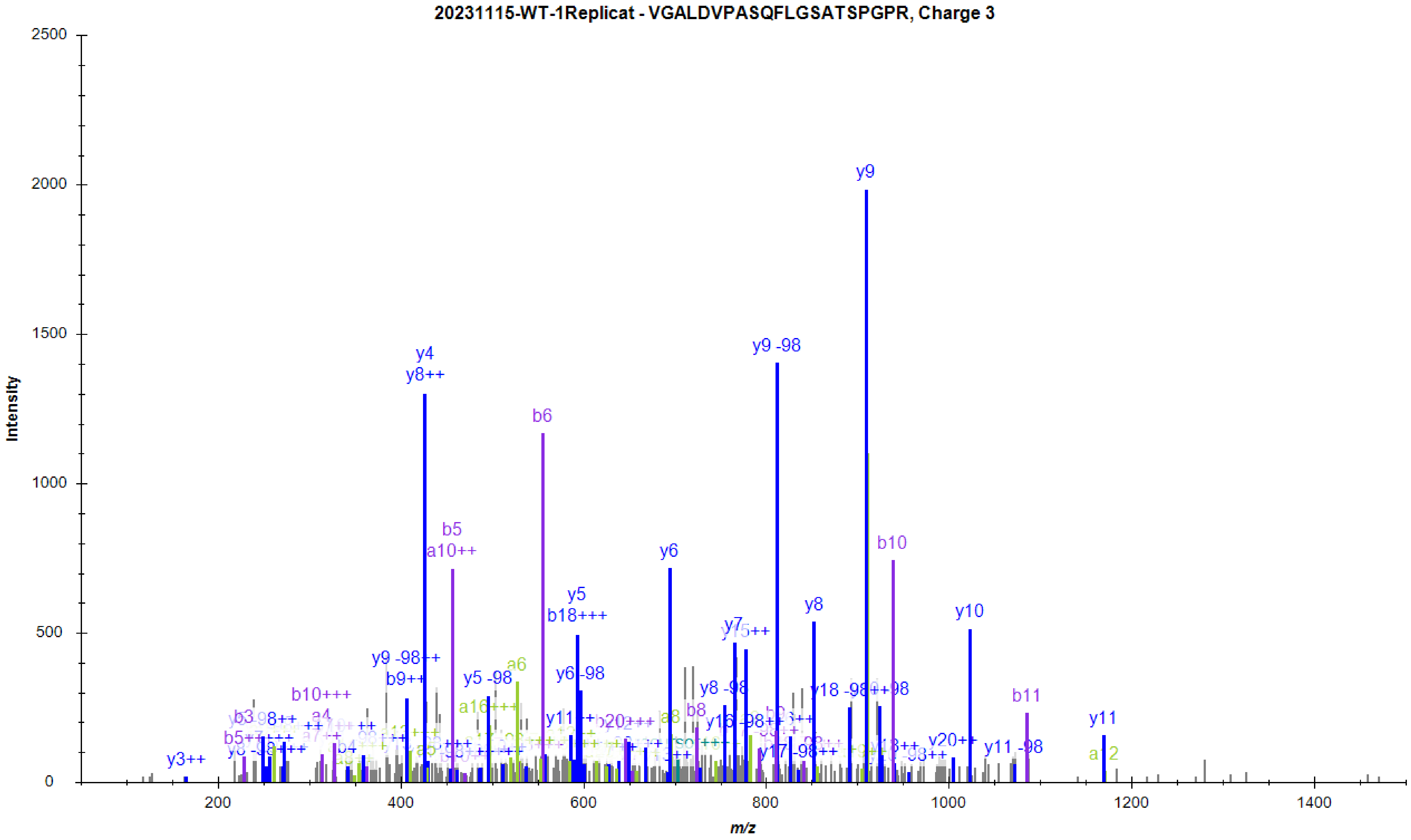
20231115-WT-1Replicat - VGALDVPASQFLGSATSPGPR, Charge 3
Intensity
m/z
y9
y9 -98
y4
y8++
b6
b5
a10++
b10
y6
y5
b18+++
y8
y10
y7
b11
y11
a12
y9 -98++
b9++
y5 -98
a6
y6 -98
a16+++
y8 -98
y18 -98++
b10+++
b3
a4
b5
y3++
b4
y11++
b20+++
a8
b8
y16 -98++
y17 -98++
v20++
y11 -98
0
500
1000
1500
2000
2500
200
400
600
800
1000
1200
1400

TSC2-S1155_2

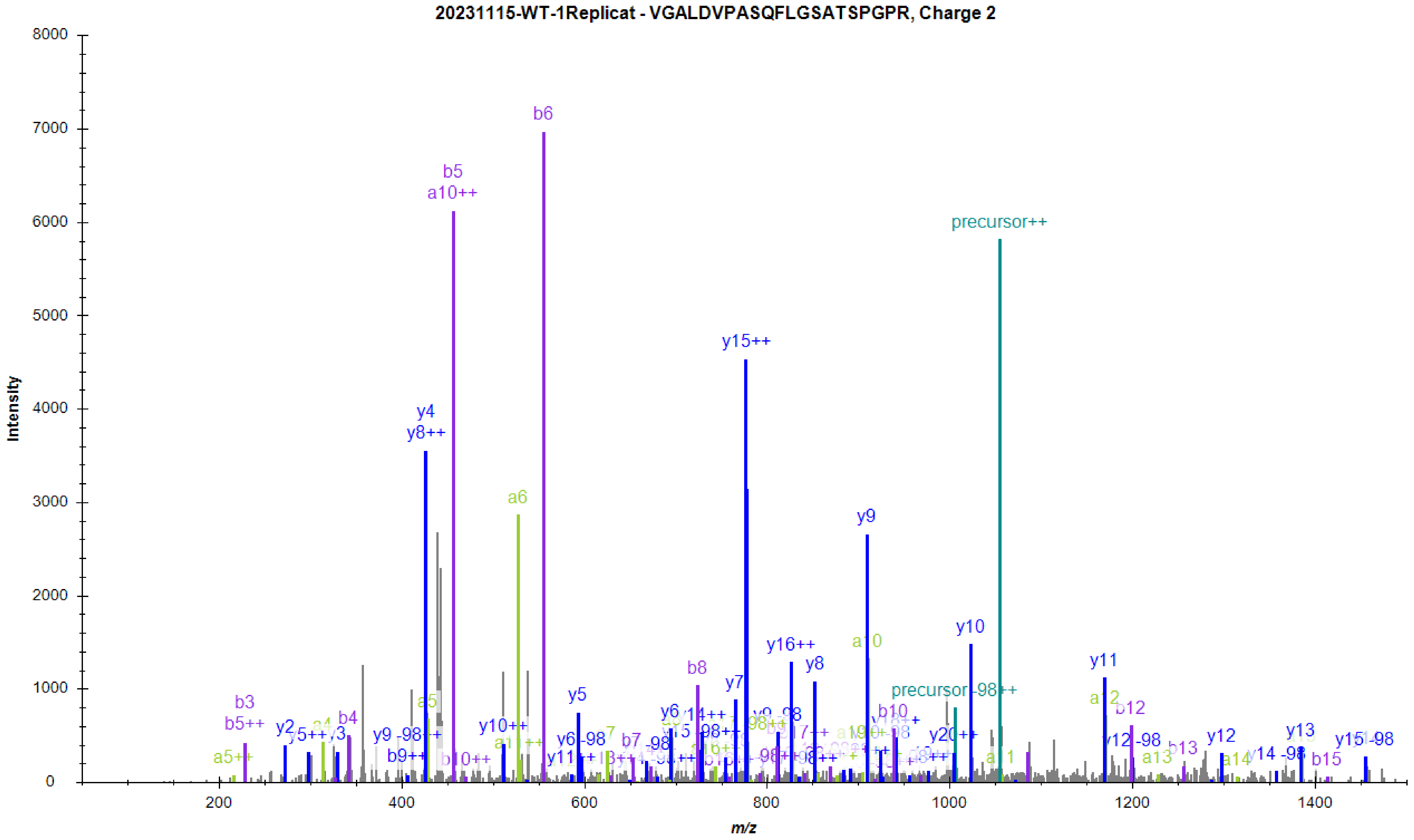
20231115-WT-1Replicat - VGALDVPASQFLGSATSPGPR, Charge 2
Intensity
m/z
0
1000
2000
3000
4000
5000
6000
7000
8000
200
400
600
800
1000
1200
1400
b6
b5
a10++
precursor++
y15++
y4
y8++
a6
y9
y16++
y8
y10
y11
b8
y7
y5
b3
b5++
b4
y2
a5
a5++
precursor -98++
b12
b10
y12
y13
y15 -98
b13
b15
a13
a14
y14 -98
y12 -98
y6 -98
y20++
y10++
y9 -98++
b9++
b10++
y11++
b7

ULK1-S423

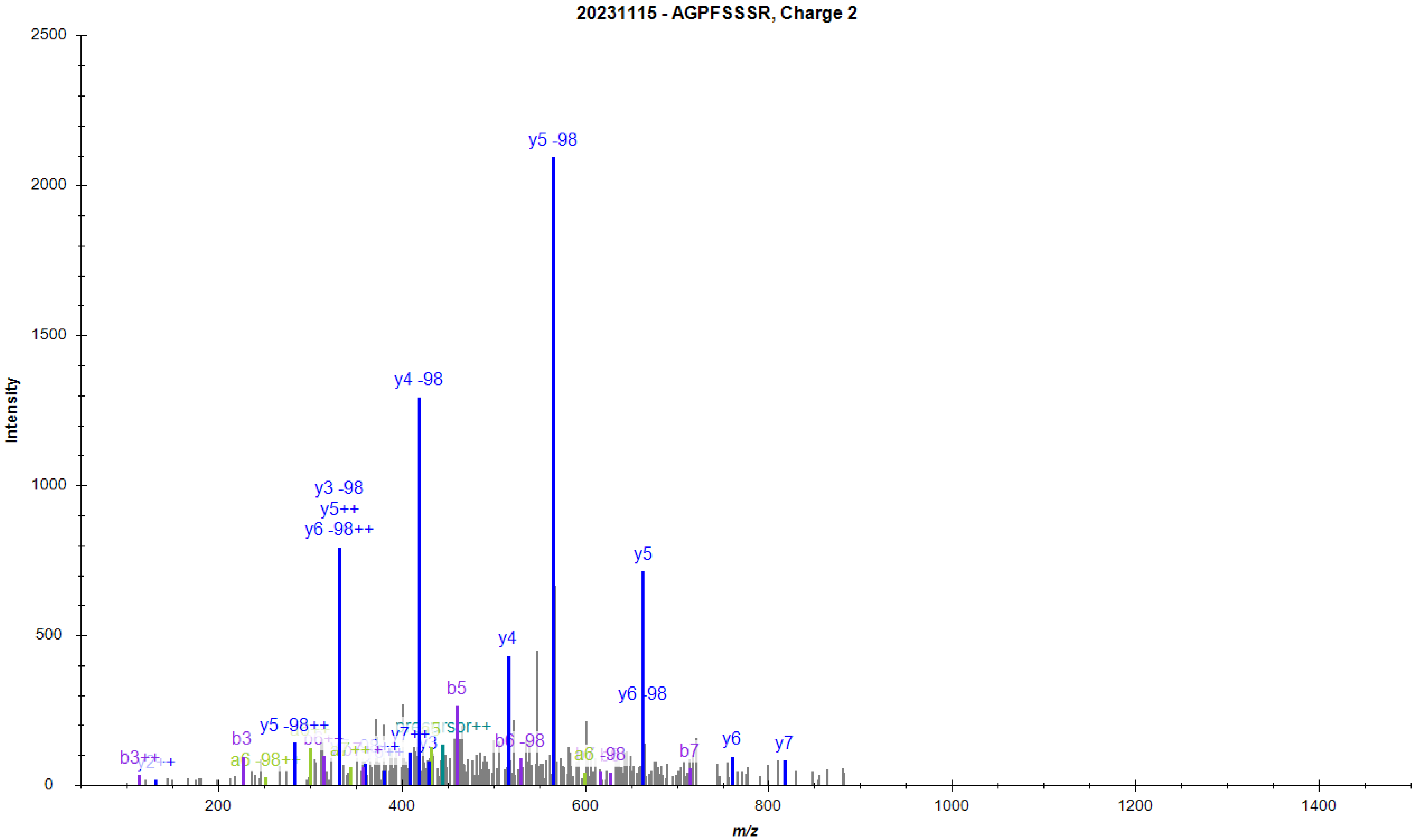
20231115 - AGPFSSSR, Charge 2
Intensity
m/z
y5 -98
y4 -98
y3 -98
y5++
y6 -98++
y5
y4
b5
y6 -98
y5 -98++
b3
b3++
b6 -98
b7
y6
y7
a6 -98
0
500
1000
1500
2000
2500
200
400
600
800
1000
1200
1400

ULK1-S450

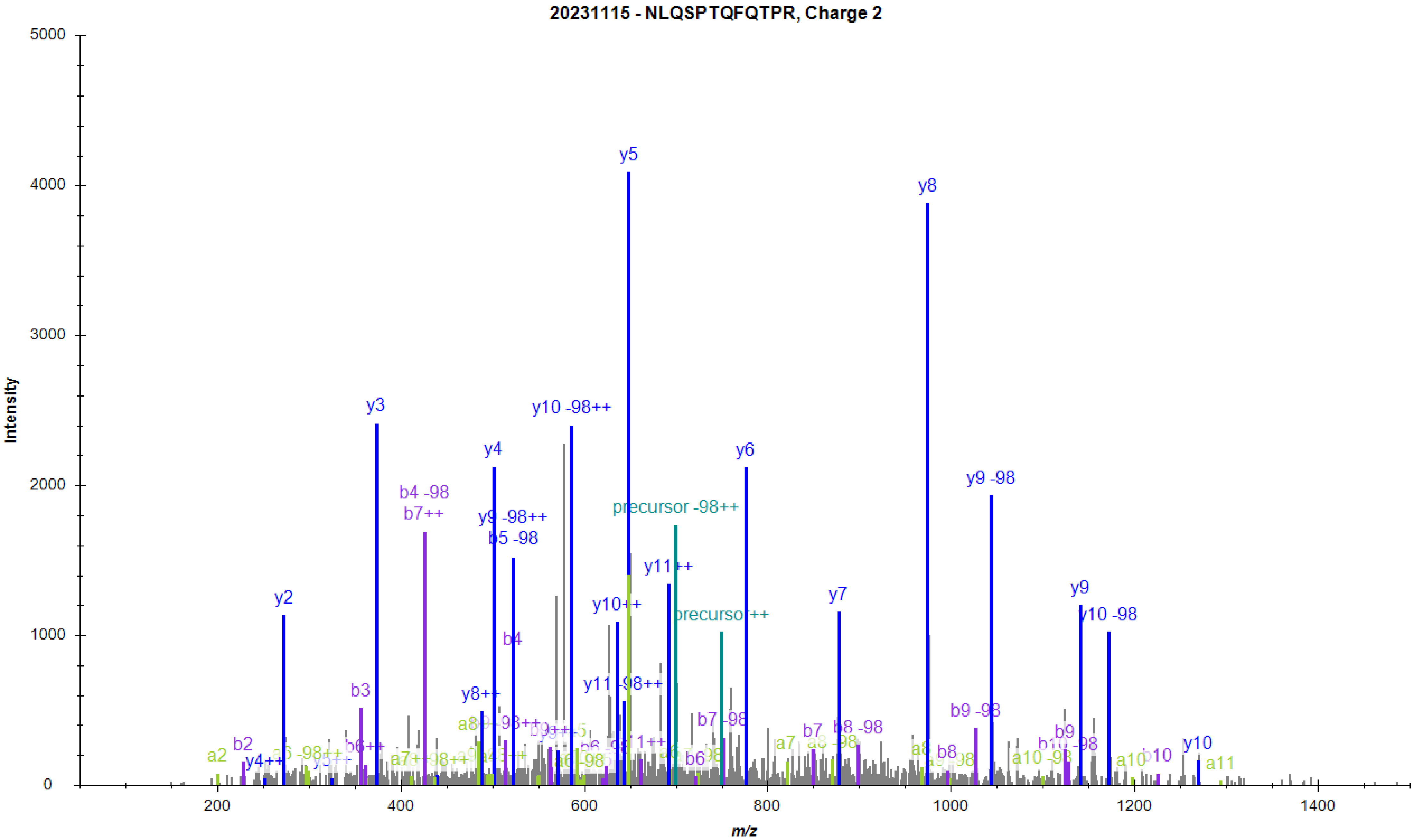

20231115 - NLQSPTQFQTPR, Charge 2
Intensity
m/z
y5
y8
y3
y10 -98++
y4
y6
y9 -98
b4 -98
b7++
y9 -98++
b5 -98
precursor -98++
y11++
y10++
precursor++
y7
y9
y10 -98
y2
b4
y11 -98++
y8++
b3
b7 -98
b9 -98
b7
b8 -98
b9
b10 -98
y10
b2
a2
y4++
a7
b8
a10 -98
a10
b10
a11

ULK1-S450-S456

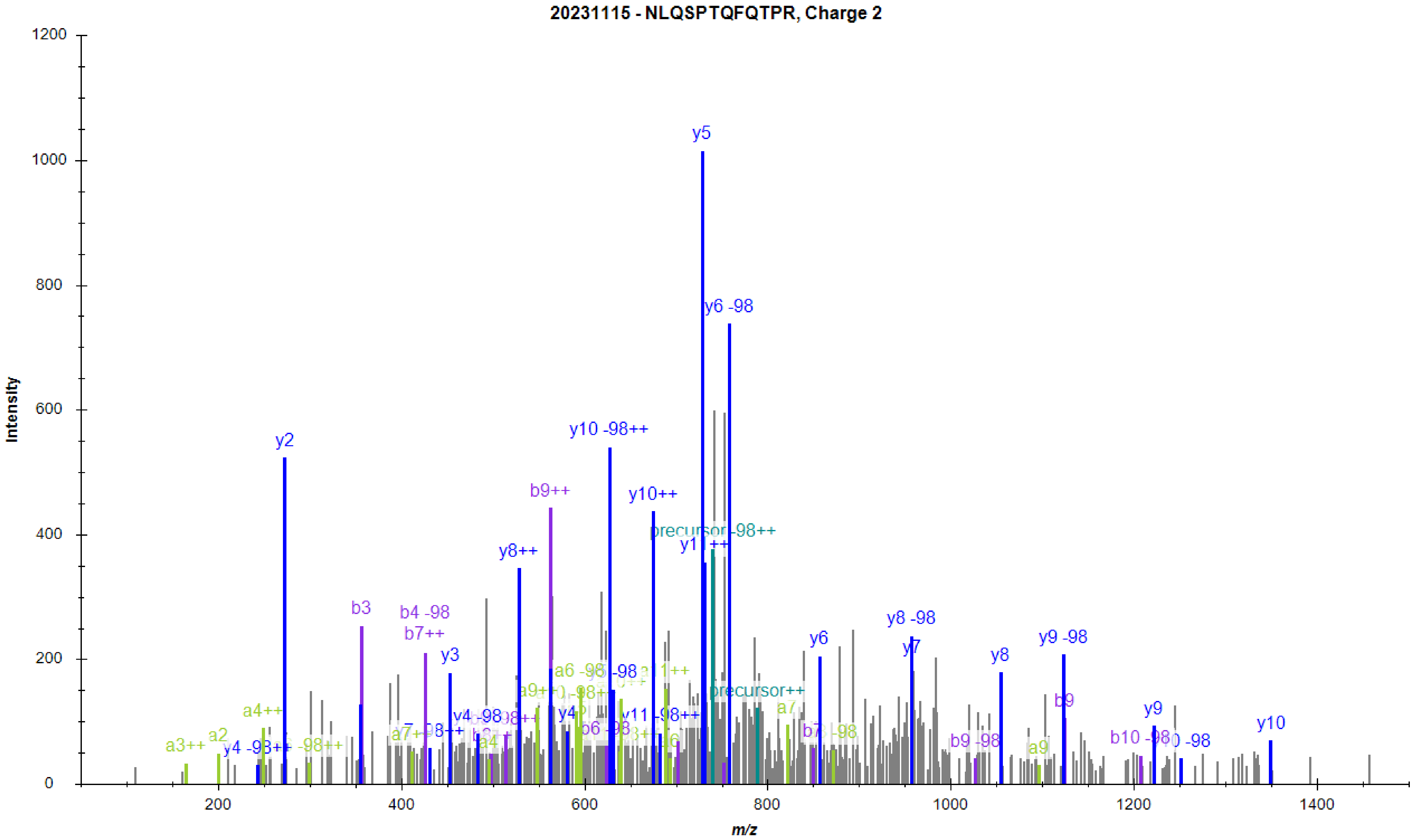

ULK1-S465-S469

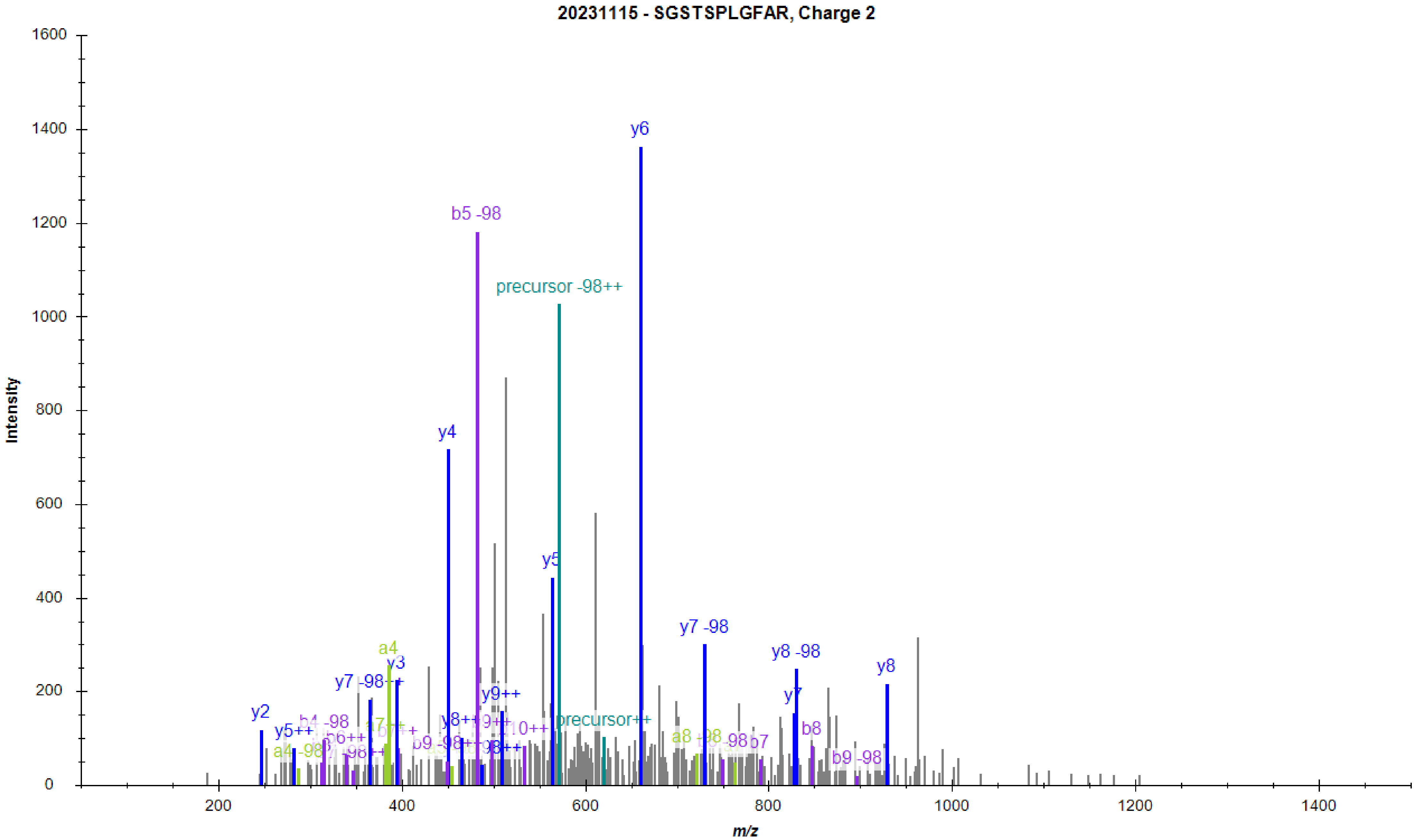

ULK1-S469

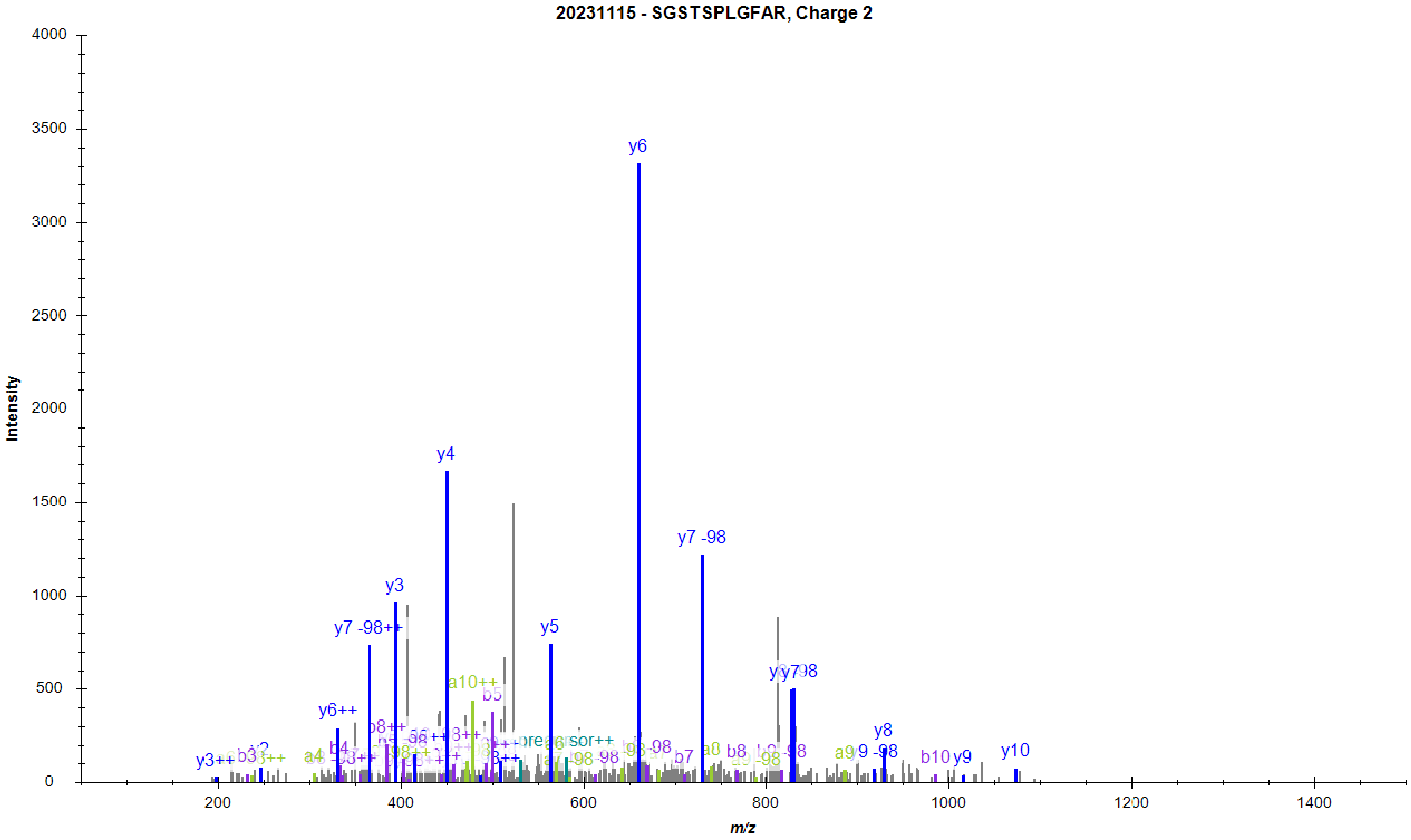
20231115 - SGSTSPLGFAR, Charge 2
Intensity
m/z
y6
y4
y7 -98
y3
y7 -98++
y5
y6++
a10++
y8
y10
b10
y9
b7
a8
b8
precursor++
y3++
b4
b3
b5

VPS26A-S315

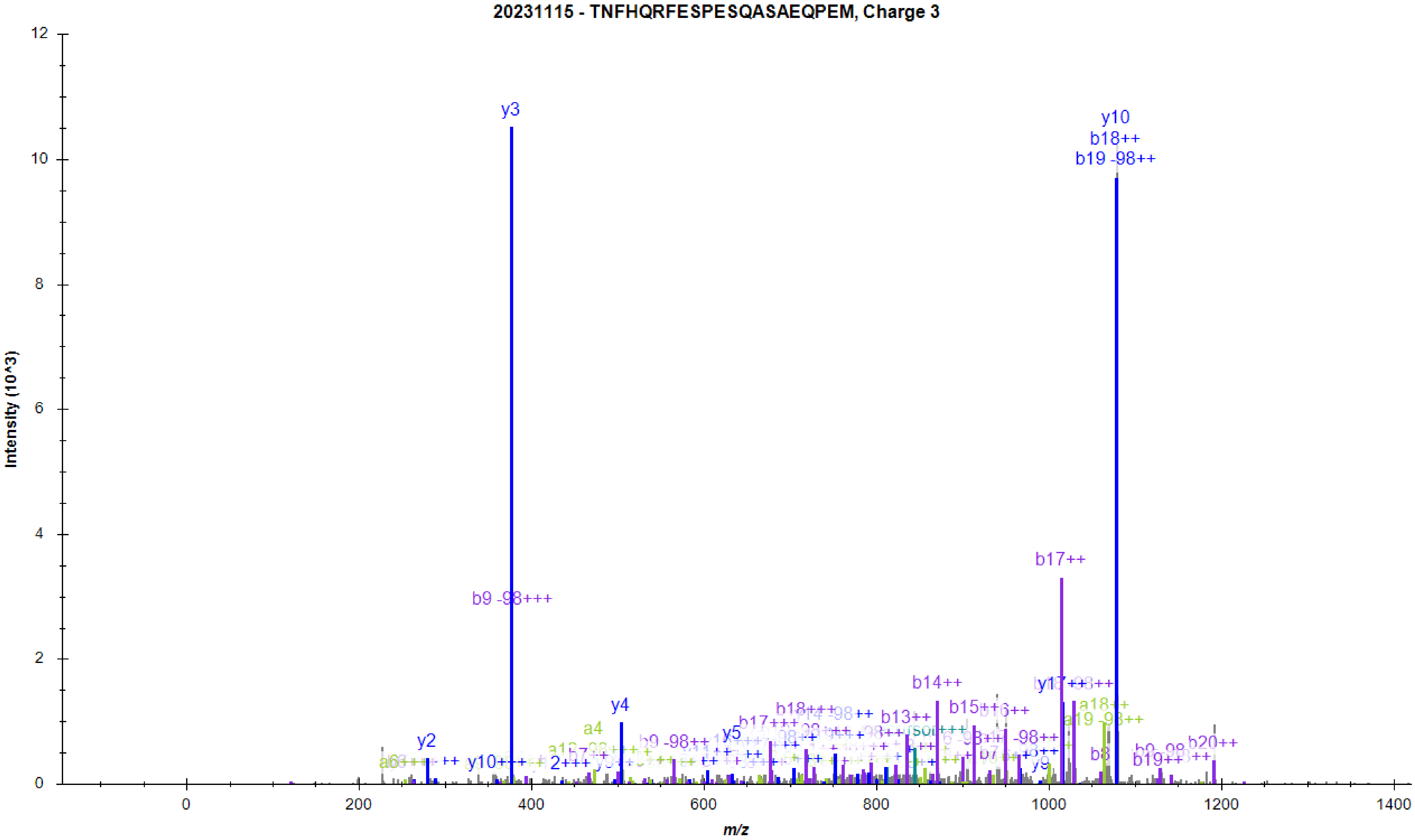
20231115 - TNFHQRFESPESQASAEQPEM, Charge 3
Intensity (10^3)
m/z
y3
y10
b18++
b19 -98++
b9 -98+++
b17++
b14++
y4
y2
a4
y5
b13++
b15++
a18++
b20++
b19++
b8
y9
0
2
4
6
8
10
12
0
200
400
600
800
1000
1200
1400

VPS26A-S315_2

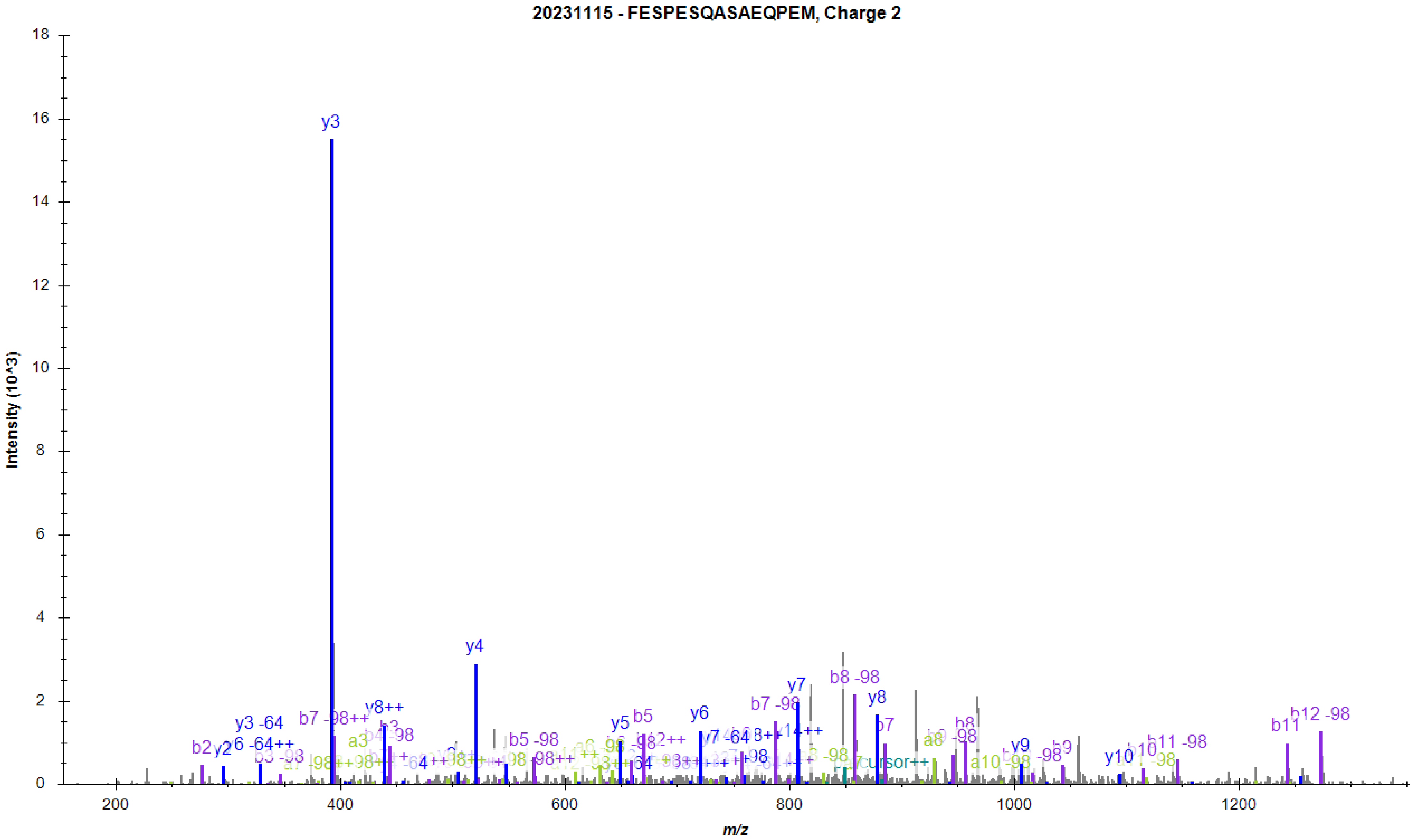

WDR62-S32

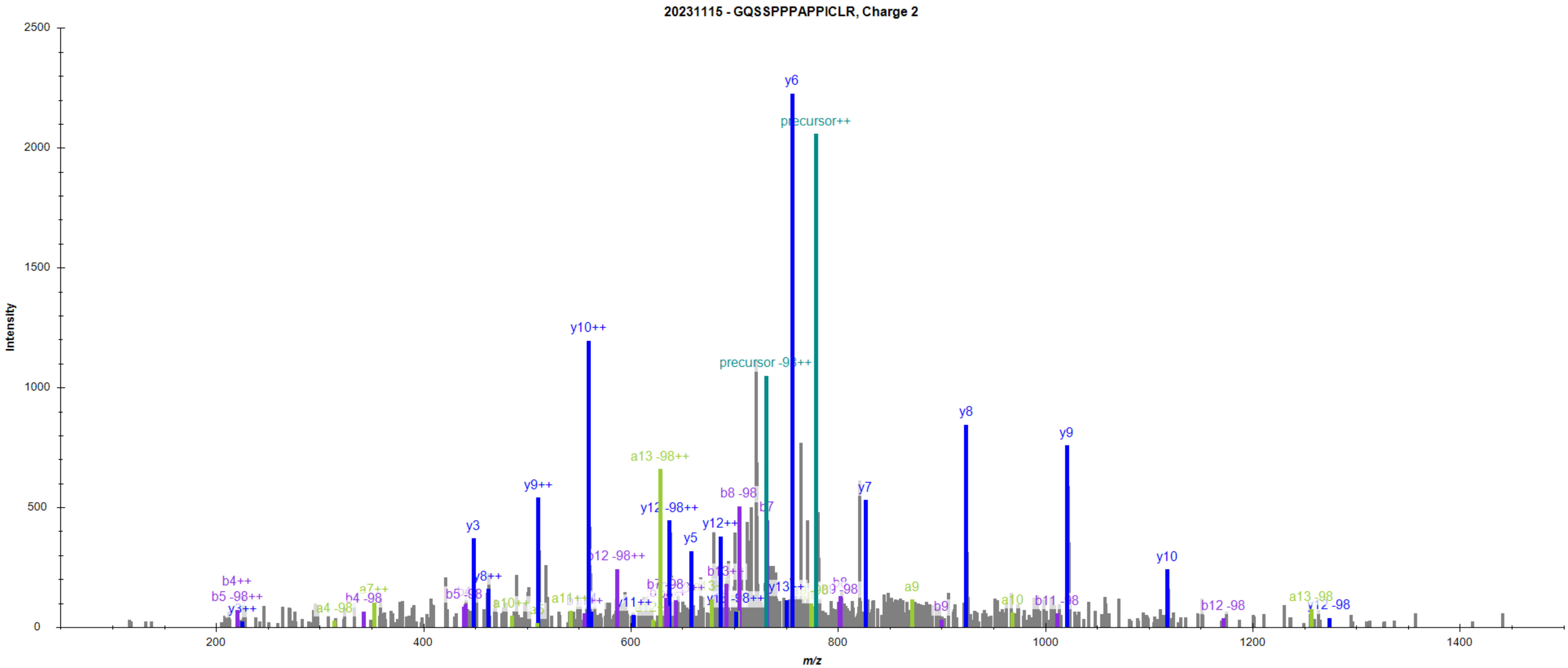

20231115 - GQSSPPPAPPICLR, Charge 2
Intensity
m/z
y6
precursor++
y10++
precursor -98++
y8
y9
a13 -98++
y9++
y7
b8 -98
y12 -98++
y3
y12++
y5
y10
